# Integrative biophysical illumination of the 3D GPCRome dynamics

**DOI:** 10.1038/s41594-026-01891-8

**Published:** 2026-10-27

**Authors:** Brian Medel-Lacruz, Adrián García-Recio, David Aranda-García, Aditya Prasad Patra, Tomasz Maciej Stepniewski, Mariona Torrens-Fontanals, Franz Hagn, Massimiliano Bonomi, Jiafei Mao, Jana Selent

**Affiliations:** 1 Research Programme on Biomedical Informatics (GRIB), Hospital del Mar Medical Research Institute (IMIM) and Department of Medicine and Life Sciences at Universitat Pompeu Fabra, Barcelona, Spain; 2 Pharmacelera, Parc Científic de Barcelona (PCB), Barcelona, Spain; 3 Department of Molecular Membrane Biology, Max Planck Institute of Biophysics, Frankfurt am Main, Germany; 4 Bavarian NMR Center (BNMRZ), Department of Bioscience, School of Natural Sciences, Technical University of Munich, Garching, Germany; 5 Molecular Targets and Therapeutics Center (MTTC), Institute of Structural Biology, Helmholtz Munich, Neuherberg, Germany; 6 Institut Pasteur, Computational Structural Biology Unit, Université Paris Cité, CNRS UMR 3528, Paris, France; 7 Beijing National Laboratory for Molecular Sciences (BNLMS), Institute of Chemistry Chinese Academy of Science (ICCAS), Beijing, China; 8 Institute for Biophysical Chemistry and Centre for Biomolecular Magnetic Resonance (BMRZ), Goethe University Frankfurt, Frankfurt am Main, Germany

**Keywords:** Molecular biophysics, Computational biology and bioinformatics, NMR spectroscopy

## Abstract

Insights into the dynamics of G-protein-coupled receptors (GPCRs) remain limited. Here we present an integrative strategy that bridges computational and experimental biophysical methods for probing protein dynamics. To illustrate its potential, we developed GPCRmd-NMR, a platform that synergistically combines molecular dynamics (MD) simulations with high-resolution nuclear magnetic resonance (NMR) spectroscopy across the three-dimensional GPCRome. The resulting dataset enables structure-resolved analysis by applying the strategy across multiple GPCRs. We provide access to underexplored functional GPCR dynamics, receptor states and sites, including microswitches, hidden ligand entrance gateways and intrinsically disordered regions. Our integrated framework offers a solution to challenges faced by both experimental and computational researchers including the MD–NMR timescale gap.

## Abstract (web summary)

Medel-Lacruz et al. introduce GPCRmd-NMR, an open platform that links molecular simulations with nuclear magnetic resonance (NMR) spectroscopy to uncover hidden conformational dynamics across G-protein-coupled receptors, improving the interpretation of experimental NMR data.

## Main

G-protein-coupled receptors (GPCRs) are an important class of cell surface receptors that sense a large variety of extracellular stimuli such as light, bitter taste, odor, hormones, neurotransmitters and mechanical stresses. The crucial roles of GPCRs in numerous physiological and pathological processes have made them popular drug targets^1^. Therefore, the molecular mechanisms of GPCR functions and regulations present a central scientific question in both academia and the health industry. During the last two decades, X-ray crystallography and, more recently, cryo-electron microscopy have provided valuable high-resolution insights into the distinct functional states of these proteins^2,3^. However, the protein structure data provided by these techniques only present certain particularly stable conformations, leaving many other potentially functional and druggable conformations of these inherently dynamic receptors underexplored. To access these ‘dark’ conformational spaces, spectroscopic and computational methods have had leading roles in mapping the structural dynamics governing GPCR signaling^4^.

Spectroscopic methods, including nuclear magnetic resonance (NMR)^5^, electron paramagnetic resonance^6^, single-molecule fluorescence resonance energy transfer (smFRET)^5^ and other optical spectroscopic approaches (for example, bimane-based fluorescence spectroscopy) map the structural changes of receptors at the atomic to nanometer scales through strategically placed reporters. Computational approaches, represented by molecular dynamics (MD) simulations, depict the structural evolution of proteins at the atomistic resolution in a full in silico setting. Both methods have provided unique structural information on ligand binding^7^, ligand-induced receptor activation^8,9^, recognition of intracellular coupling partners^10–12^ or lipid and cholesterol interactions^13,14^. Despite the great success of these methods in resolving GPCR dynamics and dynamics-governed signaling, major gaps remain in both method domains. The spectroscopic methods are restricted to a limited number of detectable sites and the types of readouts, hampering the comprehensive high-resolution mapping of GPCR dynamics. Additionally, practical challenges, including the selection of suitable reporters and readouts, data interpretation and extrapolation of results to full receptor dynamics, have restricted the accessibility of these powerful techniques. MD simulations, in contrast, while providing a fully atomistic view of proteins, are highly dependent on expertise and computational resources to reach meaningful timescales.

Over the past years, we have tackled the challenges associated with MD simulations by the development of a community-based MD approach, namely GPCRmd, which has democratized the MD simulations in the GPCR and related fields through a low-barrier, self-customizable and resource-shared open-access platform^15,16^. This landmark has paved the way for accessing receptor dynamics by integrating computational and spectroscopic approaches with unprecedented spatiotemporal resolution. Here, we showcase the power of this approach on GPCRs with a widely used, high-resolution spectroscopic technique, namely NMR^17–19^ in both solution and solid state. Our GPCRmd-NMR platform streams in silico MD simulation to spectroscopic features, unveiling previously hidden structural dynamics of GPCRs across functional states. It unlocks the potential of spectroscopic and computational biophysical methods in depicting and deciphering functionally relevant dynamics at the three-dimensional (3D) GPCRome level with over 38 billion NMR signals assigned to more than 7 million GPCR conformations.

Our work represents an innovative framework that can be expanded feasibly to other spectroscopic and computational methods, presenting a gateway to data science-driven biophysical and structural biology method integration. Ultimately, the established GPCRmd-NMR platform can also serve as a template for large-scale integrative interrogation of function-relevant protein dynamics of other important protein families (for example, kinome).

## Results

### Chemical shift (CS) streaming for the dynamic 3D GPCRome

**Fig. 1:**
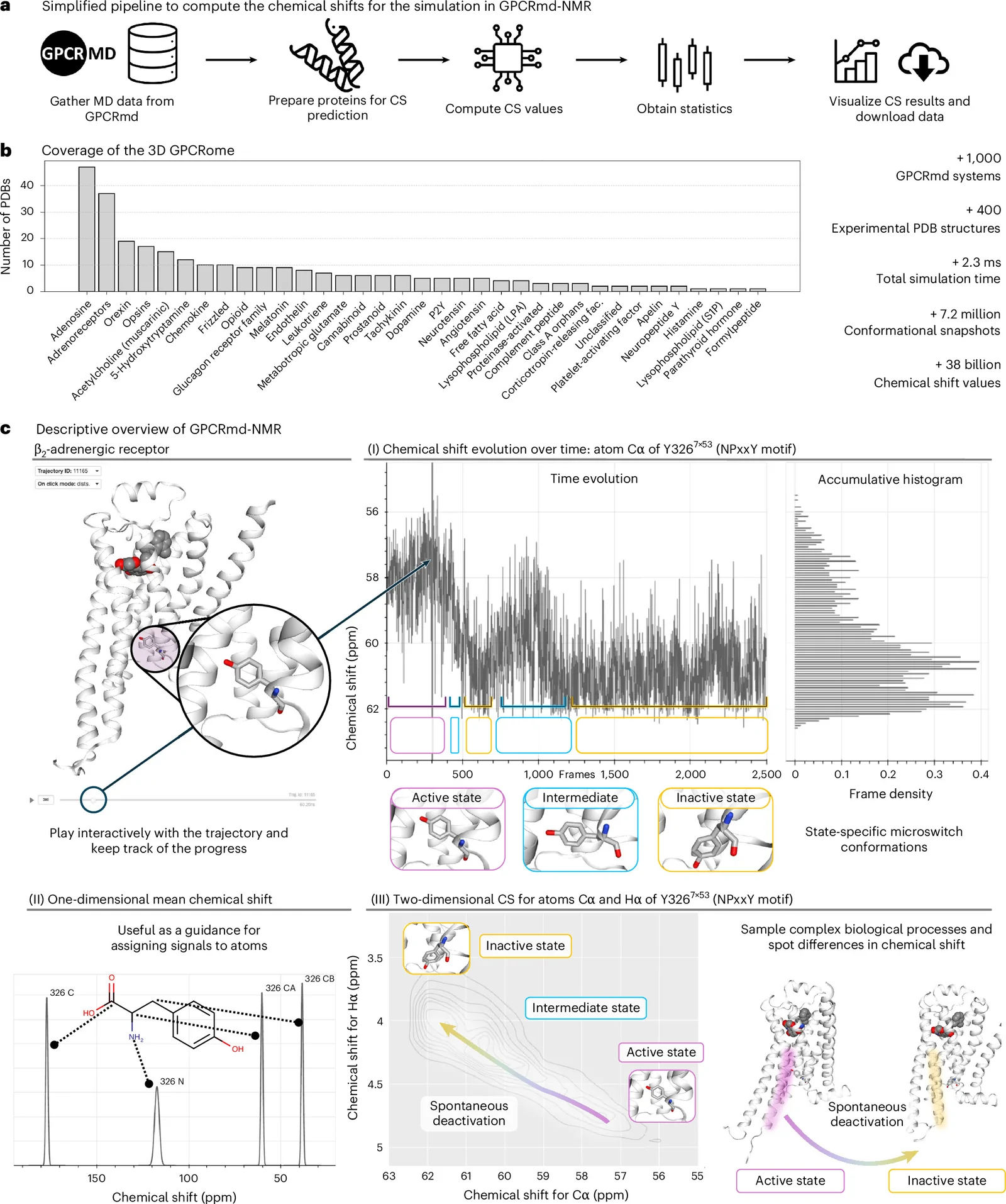
Streaming CSs for GPCRs. **a**, Pipeline for computing CSs from GPCR MD simulations, with data available as downloadable CSV files. **b**, Coverage of the 3D GPCRome. Summary of GPCR families and the collected simulation and CS data. **c**, Overview of the GPCRmd-NMR platform. β2AR (GPCRmd 121, PDB 4LDE) is used as a case, highlighting Y326^7×53^ from the NPxxY motif during receptor inactivation. Three visualization modes are shown: (I) a time series of the Cα CS, revealing active, intermediate and inactive states associated with Y326^7×53^ side-chain reorientation; (II) a 1D CS view showing the mean predicted side-chain atom signals as a simulated 1D NMR spectrum; and (III) a 2D CS view displaying the Cα–Hα of Y326^7×53^ CS distribution as a simulated 2D NMR spectrum, where distinct predicted signals correspond to different conformational states of Y326^7×53^ during GPCR activation and inactivation.

We focused on the CS, a site-specific NMR parameter that is highly sensitive to protein structure, molecular interaction and local environment. In the endeavor of forward computational prediction of CSs from protein structures, a number of high-performance predictors have been developed^20^. Some representative and widely used predictors are SHIFTX2 (ref. ^21^), SPARTA+^22^ and the latest UCBShift (version 2.0)^23^. These predictors primarily rely on machine learning and are trained on experimental protein NMR data, capturing the sophisticated relationship between protein structures and CSs. Taking these well-established CS predictors, we integrated the in-frame CS prediction with MD simulations (Fig. 1a) across the 3D GPCRome.

Built on our GPCRmd database, a large-scale collection of high-quality and unified GPCR MD data, we computed the CS evolution over 1,000 GPCR MD systems belonging to more than 400 experimentally solved structures of GPCRs in different functional states (for example, inactive, intermediate and active; Fig. 1b). This dataset illuminates the CS fluctuations along with the structural dynamics at a 0.2-ns resolution. In total, our CS exploration of over 2.3 ms of accumulative MD simulation time has yielded 7.2 million conformational snapshots with assigned CS profiles and 38 billion CS values (Fig. 1b). These data are made freely accessible through GPCRmd-NMR (https://www.gpcrmd.org/gpcrmd-nmr-tool), which is equipped with tailored web-based streaming, visualization and data analysis tools (Fig. 1c).

### GPCRmd-NMR aligns with experimental NMR observations

**Fig. 2:**
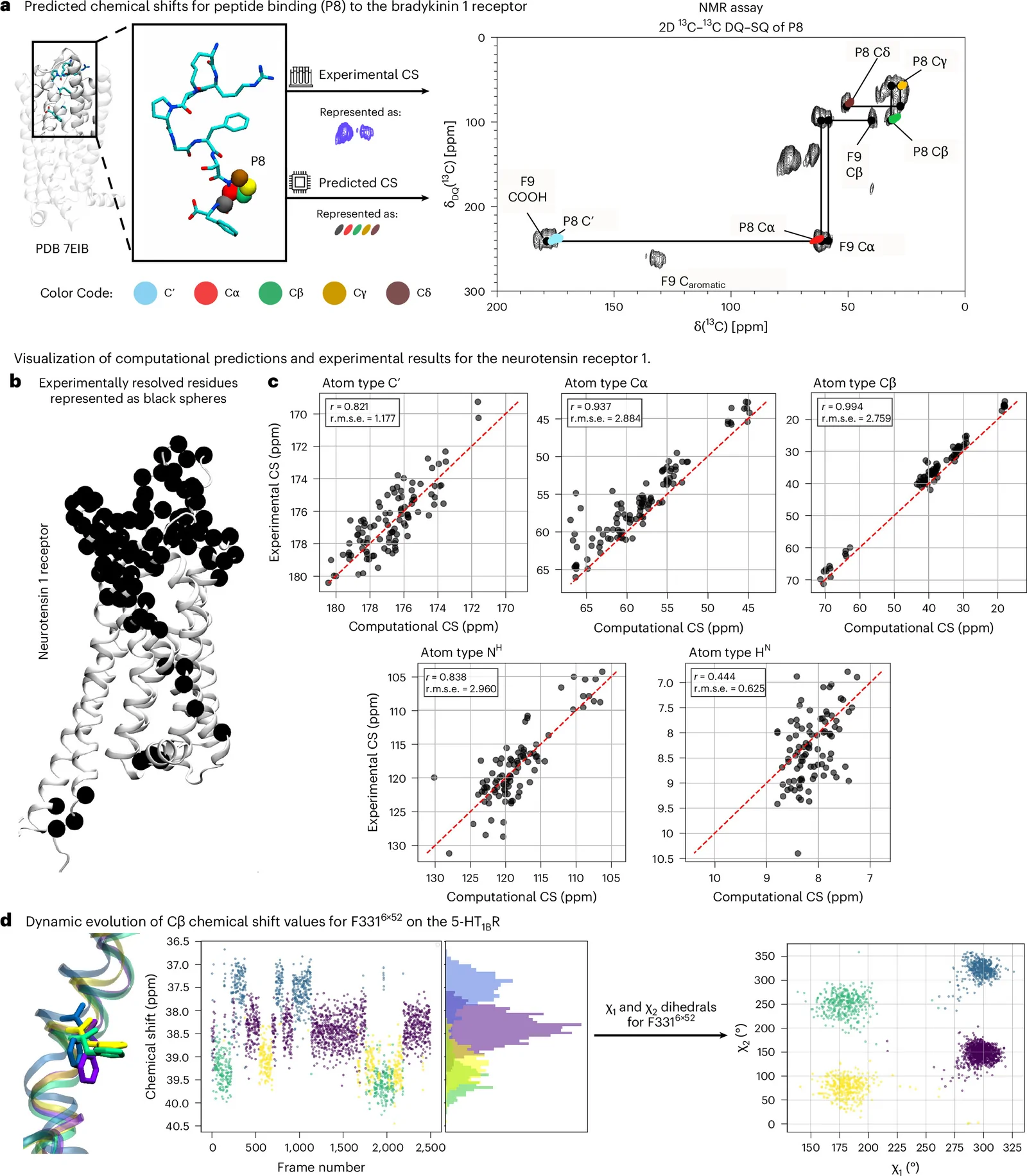
Benchmarking computational and experimental NMR CSs. **a**, Predicted CSs for peptide P8 bound to the B1R (GPCRmd 2095, PDB 7EIB) overlaid on the experimental ^13^C–^13^C DQ–SQ spectrum, showing notable agreement for C′ (light blue), Cɑ (red), Cβ (green), Cɣ (orange) and Cδ (brown) atoms. **b**, NTS_1_R residues with experimental backbone CS assignments (black spheres), taken from Mohamadi et al.^26^ (BMRB 51907, GPCRmd 2097, PDB 4BWB). **c**, Correlation between predicted (SHIFTX2) and experimental CSs for replica 1, showing strong agreement for heavy atoms and lower correlation for hydrogen atoms (replica plots 1–10 in Supplementary Figs. 2–6). r.m.s.e., root-mean-square error. **d**, Dynamic evolution of Cβ CS values for F331^6×52^ on the 5-HT_1B_R (GPCRmd 85, PDB 4IAQ). This residue exhibits four distinct conformational states over the MD, differentiated by the *Χ*_1_ and *Χ*_2_ dihedral angles (color coded). Source data

We challenged the real-life performance of GPCRmd-NMR in the context of solid-state and solution NMR spectroscopy. For this, we picked up two GPCR systems for which high-quality NMR data were available^24^. First, we validated the performance of our approach on peptide binding to the human bradykinin 1 receptor (B1R)^25^, a peptide receptor with crucial roles in chronic pain and inflammation. In the first step, we performed MD simulations of the B1R–peptide agonist complex (three replicates of 500 ns). During MD simulations, the P8 residue of the peptide ligand adopts a fully embedded configuration as confined by the peptide binding cavity during the MD simulation (GPCRmd 2095, PDB 7EIB). Using GPCRmd-NMR, we then reconstructed the two-dimensional (2D) ^13^C–^13^C double-quantum single-quantum (DQ–SQ) spectral pattern for all carbon atoms of the P8 residue of the peptide ligand (Fig. 2a). Previously, the CS data of the peptide agonist DAKD in complex with B1R were obtained using DNP-enhanced solid-state NMR spectroscopy^24^, which were collected at low temperature (~100 K) and, therefore, present the conformational distribution of the bound ligand. Remarkably, we found that both the predicted (multicolored) and experimental (dark blue) NMR signals of P8 exhibit substantial overlap (Fig. 2a, right), indicating that our method aligns the structure plasticity with NMR observations. Additional 500-ns replicate simulations reproduced an identical 2D ^13^C–^13^C DQ–SQ spectrum (Supplementary Fig. 1). The success of reproducing a small-sized low-temperature NMR dataset encouraged us to explore more comprehensive NMR data streaming at more physiological temperatures.

As an example, we used the solution NMR spectroscopic study on *Rattus* *norvegicus* (rat) neurotensin receptor 1 (NTS_1_R) with data retrieved from the Biological Magnetic Resonance Data Bank (BMRB). The NTS_1_R senses and responds to the neuropeptide neurotensin, which modulates dopamine signaling, regulates body temperature and influences pain perception. Mohamadi and coworkers^26^ performed extensive backbone CS assignment on this receptor bound to the natural agonist neurotensin 1. The dataset contains CS information on backbone signals, including the atom types C′ (carboxyl), Cɑ, Cβ, N^H^ and H^N^ (BMRB 51907). We used this rich dataset to evaluate systematically the performance of different CS predictors in GPCRs (SHIFTX2, SPARTA+ and UCBShift version 2.0). We conducted MD simulations (ten replicas of 1 μs, GPCRmd 2097, PDB 4BWB) mirroring the experimental conditions used for recording the NMR data^26^.

SHIFTX2-based predictions show excellent agreement between computational predictions and experimentally determined CSs for the targeted residues (Fig. 2b,c), with strong correlations observed for heavy atoms (*r* = 0.80 to 0.99) (Supplementary Table 1). In contrast, predictions for H^N^ atoms show lower correlations (*r* = 0.42–0.46), which is expected given that they are more sensitive to subtle inaccuracies in structural or electronic modeling^27^. Notably, the consistent correlation across replicates further underscores the robustness of integrating CS predictors with MD simulations (Supplementary Figs. 2–6). We also evaluated the performance of SPARTA+ and the recently published UCBShift (version 2.0) predictor, both of which showed comparable results to SHIFTX2 for heavy atoms (Supplementary Figs. 7–16 and Supplementary Table 1), demonstrating a general consistency among the predictors.

To further support our findings, we expanded the benchmark by including four additional NMR datasets from the BMRB covering various GPCR systems, including the β_1_-adrenergic receptor (β_1_AR)^28,29^, β_2_AR^30^ and adenosine A_2__A_ receptor^31^ (Supplementary Table 2). These five datasets represent all currently available GPCR CS entries deposited in the BMRB, comprising a total of 679 experimental CS measurements. Remarkably, this extended analysis confirms the robust performance of our approach across different receptors and heavy atom types (Supplementary Fig. 17; *r* = 0.82–0.99).

### Illuminating underexplored GPCR dynamics through GPCRmd CS streaming

Beyond conventional averaged biophysical parameters or their distributions from spectroscopic studies, our computational framework enables the continuous tracking of spectroscopic parameters throughout the fully atomistic structural evolution (Fig. 1c, top). This approach is particularly valuable in challenging cases where CS values arise from highly heterogeneous structural ensembles. Here, we demonstrate this capability for the 5-hydroxytryptamine 2B receptor (5-HT_1B_R)—a promising drug target for pulmonary arterial hypertension, valvular heart disease and related cardiopathies^32^. Monitoring the Cβ CS evolution of the F331^6×52^ (Fig. 2d), a functionally relevant residue in the ligand binding pocket, we find that it samples four distinct conformational states in the apo 5-HT_1B_R over time (GPCRmd 85, PDB 4IAQ)^33^. The conformational exchange over these four microstates occurs at the nanosecond scale and would typically result in one averaged signal when studied by NMR spectroscopy. Such a case suggests that a computational framework like GPCRmd-NMR can reveal underexplored structural dynamics by integrating MD simulations with the computation of relevant biophysical readouts. This is demonstrated in a real case below.

**Fig. 3:**
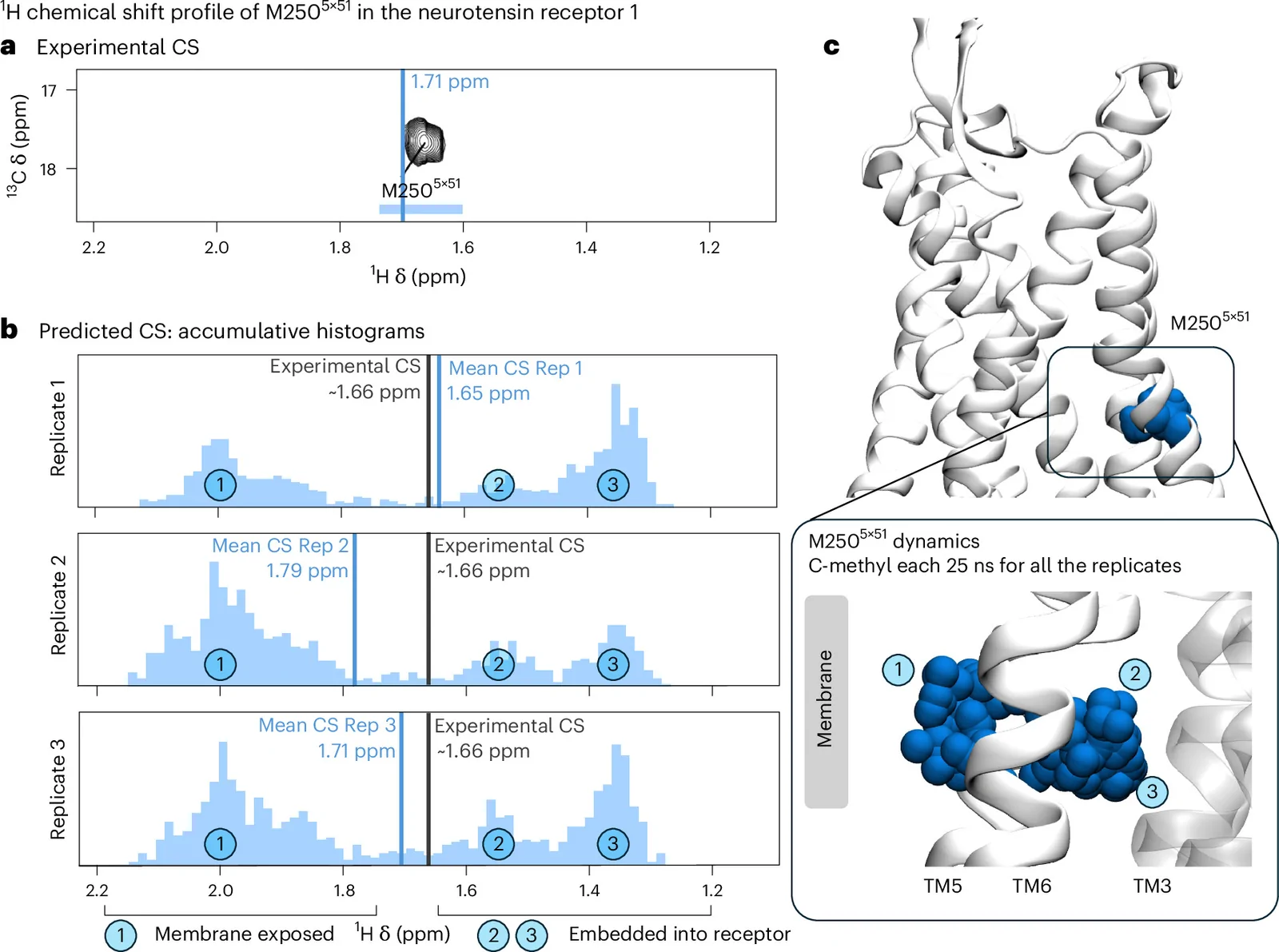
Linking conformational states and CS variability of methionines in the NTS_1_R and GPCRmd-NMR potential applications. **a**, ^1^H CS profile of M250^5×51^ in the NTS_1_. Experimental CS data for M250^5×51^ extracted from Bumbak et al.^34^ (Fig. 2). The predicted average of 1.71 ppm, obtained from three MD replicates, is indicated in blue. **b**, Predicted CS data for M250^5×51^ over a distribution of different methyl conformations based on three 1-μs simulations. The average predicted CS (1.71 ppm) matches the axis of the experimental CS (1.66 ppm). Such averaging indicates the fast exchange of these side-chain conformations, in line with the MD findings. The blue circled numbers 1–3 represent the three different CS distributions of M250^5×51^ that can be seen for each replicate, with their respective structural depiction in **c**. **c**, Structural depiction of the NTS_1_R and the location of M250^5×51^ with different side-chain conformation. Source data

Taking the example of NTS_1_R as studied by Bumbak and coworkers using solution NMR spectroscopy^34^, we reconstructed the ^1^H NMR spectroscopy of methionine methyl groups in this receptor using our GPCRmd-NMR approach (Fig. 3). To explore how changes in receptor structure induce CS alterations in the NTS_1_R, we performed MD simulations (three replicas of 1 μs) for the apo-NTS_1_R (GPCRmd 2099, PDB 6Z66) and computed the CS profile. Specifically, we focused on the M250^5×51^ residue in the transmembrane helix TM5, which is located next to the P5.50 kink in a functionally highly relevant region. Intriguingly, GPCRmd-NMR revealed that the experimentally observed average CS (~1.66 ppm) of the M250^5×51^ methyl group (Fig. 3a) emerges from three different CS populations (Fig. 3b). CS population 1 (~2.00 ppm) corresponds to M250^5×51^ oriented toward the membrane. In contrast, populations 2 (~1.55 ppm) and 3 (~1.35 ppm) correspond to M250^5×51^ facing the receptor interior. An interesting observation is that the observed CS populations arise from a heterogeneous set of M250^5×51^ side-chain conformations. For example, although the membrane-embedded M250^5×51^ displays two distinct CS populations (Fig. 3), only one conformational cluster (cluster 3; Supplementary Fig. 18) is detected. This finding indicates that CSs and conventional conformational descriptors are not directly correlated, as CSs reflect not only side-chain conformational changes but also variations in the local environment.

Importantly, the close agreement of the average CS across the different CS populations with the experimental NMR signal supports the relevance of the sampled conformational space. This case study also confirms that methyl protons can serve as reliable predictors of fast side-chain exchange. This is consistent with previous reports showing that protons not influenced by electronic shielding carry less predictive errors in contrast to donor protons (for example, NH)^23,35^. In summary, this finding uncovers a previously unrecognized rapid conformational switching that occurs near key receptor activation sites, highlighting dynamic features that are critical for receptor function yet elusive to conventional experimental approaches.

### Exploring intrinsically disordered protein regions (IDRs) and interface contacts

**Fig. 4:**
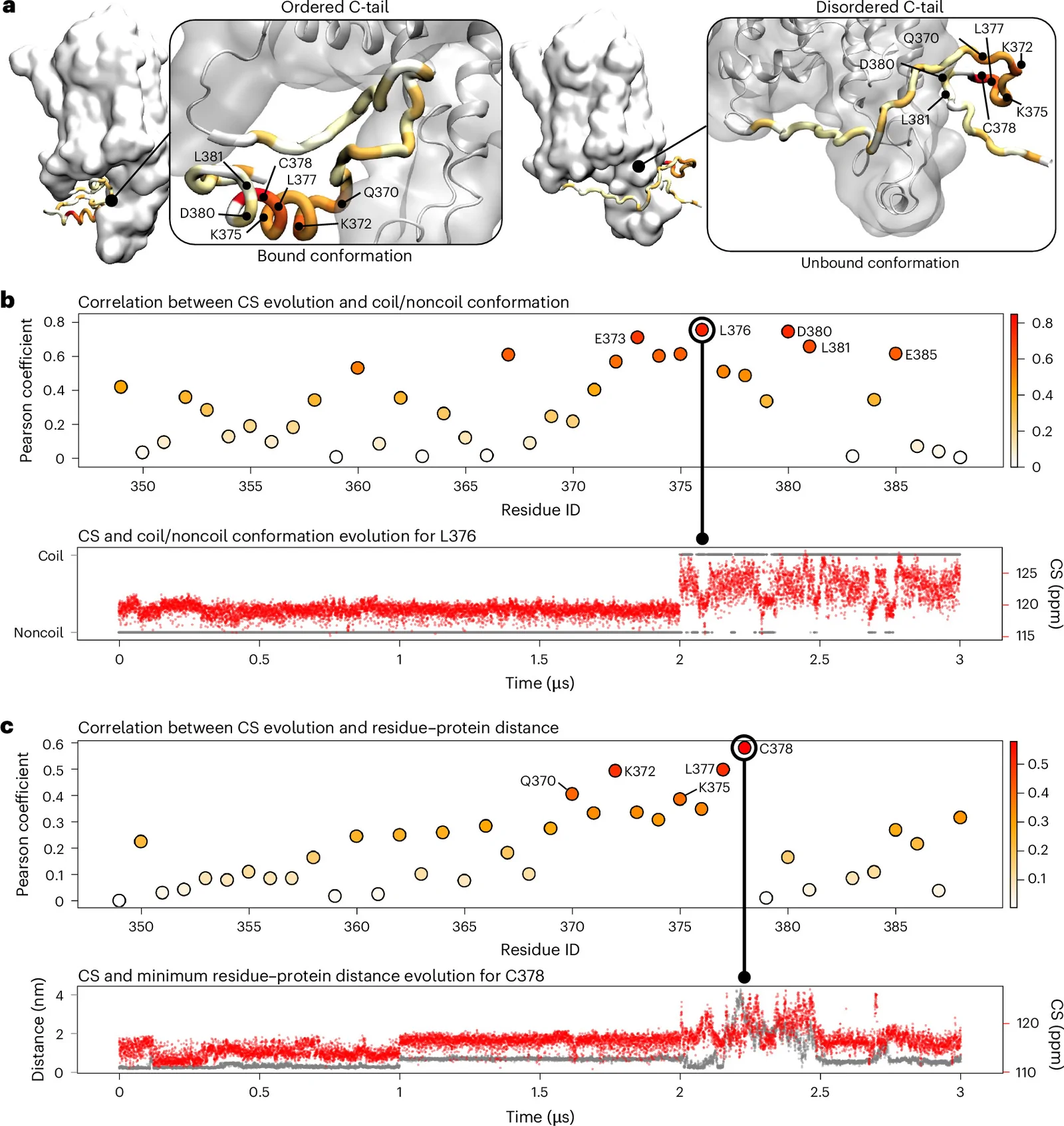
CS correlations reveal disorder, ordering and transient receptor contacts in the β_2_AR CT. **a**, Representative conformations of β_2_AR CT MD simulations from Heng et al. for the ordered CT and the disordered CT. **b**, Pearson correlation between the time evolution of predicted NH CSs for each CT residue and its coil versus noncoil classification. Residues within the helix-forming segment (368–378) show the strongest correlations (L376 shown as an example). **c**, Correlation between NH CS evolution and residue–receptor proximity, highlighting residues whose CS values are strongly modulated by transient contacts with the intracellular receptor core (C378 shown as an example). Source data

We also evaluated the ability of the GPCRmd-NMR framework to differentiate IDRs from ordered regions. For this analysis, we used the public dataset from Heng et al.^30^, who performed MD simulations of the β_2_AR and deeply analyzed the behavior of the carboxyl terminus (CT) region as an IDR. Using their MD data, we computed CS values for three independent replicates highlighted by the authors, each simulated for 1 μs. These replicates exhibit distinct CT conformations (Fig. 4a); two display a predominantly ordered (noncoiled) CT, while the third adopts a disordered coil conformation.

To assess whether CS values correlate with the disorder–order transition of the CT, we computed Pearson correlation between the temporal evolution of the NH CS values and the corresponding coil versus noncoil state of the CT (Fig. 4b). Notably, residues involved in helix formation (368–378) exhibited strong CS correlations, which is further supported by the secondary structure evolution observed for the top-correlated residues (that is, L376). These results indicate that structural ordering within the CT can be effectively captured through the combination of CS predictions and MD simulations.

In addition to these conformational changes, we observe that the CT interacts with the receptor core at the intracellular side. This interaction likely contributes to alterations in CS values for the bound CT relative to the unbound state because of shielding effects exerted by the receptor core. This hypothesis is supported by the strong Pearson correlation observed between the temporal evolution of NH CS values (for each CT residue) and their closeness to the receptor core at the intracellular side (Fig. 4c).

In summary, these results demonstrate that the GPCRmd-NMR framework sensitively captures both disorder–order transitions in IDRs and their transient interface contacts with the receptor core, highlighting its capacity to link dynamic structural ensembles with experimentally relevant CS signatures.

### Integrative enhancement of spectroscopic pipelines through GPCRmd-NMR

Though being pivotal to accessing GPCR dynamics, the current spectroscopic approaches to GPCRs suffer from several key bottlenecks spanning over their full pipeline, namely the (1) signal assignment including higher spectroscopic dimensions; (2) reporter selection; and (3) integrated hypothesis generation and spectroscopic solutions. Encouraged by the abovementioned performance of GPCRmd-NMR, we envision a number of computation-driven approaches that could immediately boost these spectroscopic efforts on GPCRs.

**Fig. 5:**
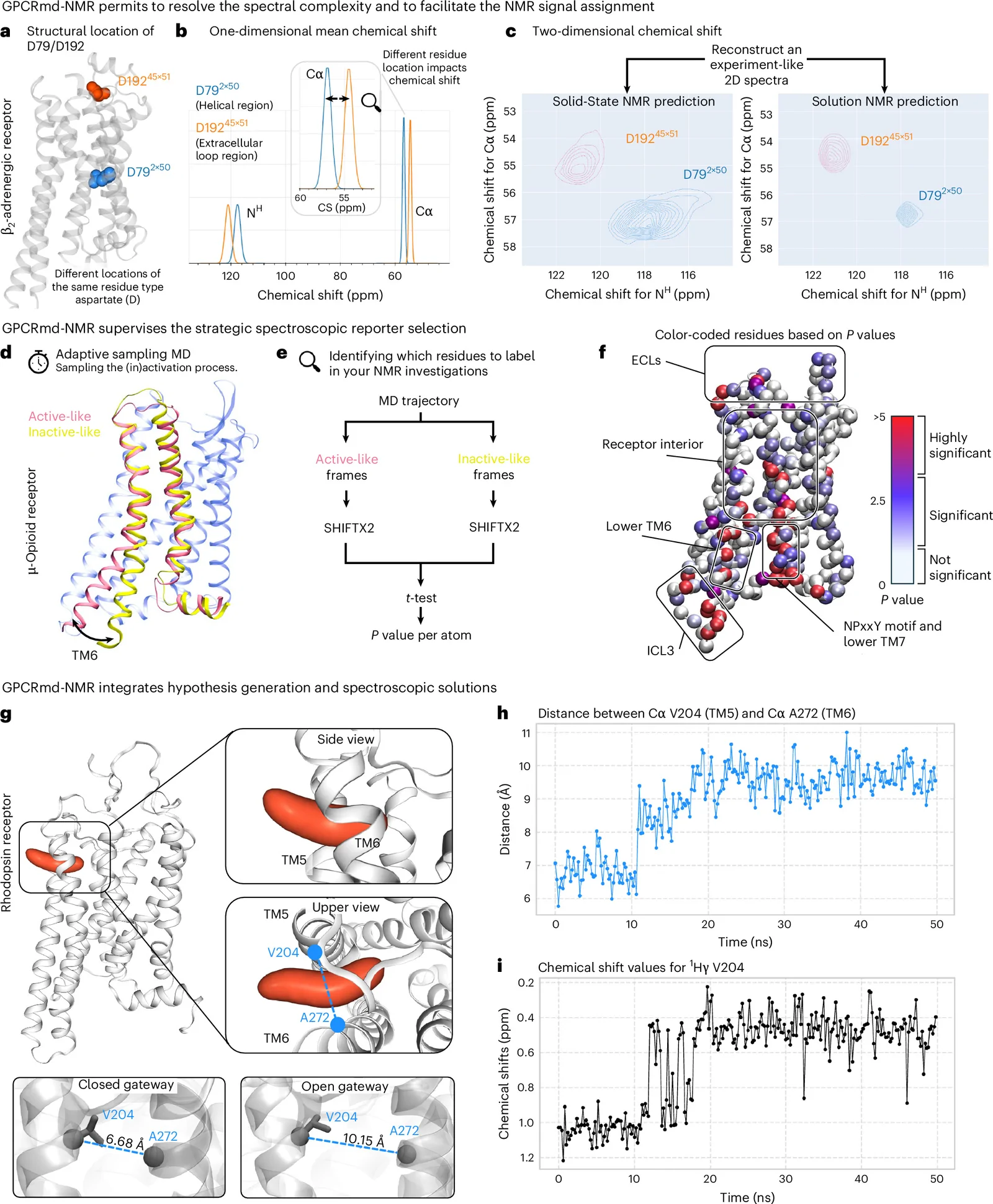
Integrative enhancement of spectroscopic pipelines with GPCRmd-NMR. **a**, Structural locations of D79^2×50^ and D192^45×51^ in the β_2_AR. **b**, One-dimensional CS predictions distinguish D79^2×50^ and D192^45×51^ indicating that the two signals are significantly different. **c**, Two-dimensional CS predictions for solid-state NMR (distributions) and solution NMR (mean values) for the two aspartates. **d**, Classification of active-like (pink) and inactive-like (yellow) μOR conformations based on TM6 displacement. **e**, Schematic workflow of CS computation using SHIFTX2. **f**, μOR colored by *P* values of backbone ^15^N CSs. Red indicates highly significant changes, blue indicates significant changes and white indicates residues with no significant differences between states. **g**, Density map (orange) of a lipid tail in the rhodopsin cryptic pocket (GPCRmd 872, PDB 5DYS). **h**, Cα distance between V204 and A272 over time (blue). **i**, ^1^Hγ CS of V204 (black), correlated (*r* = 0.83) with the V204–A272 distance.

An important strength of GPCRmd-NMR is its ability to simplify NMR signal assignment by resolving the inherent spectral complexity of GPCR dynamics. Here, we provide a user-friendly solution based on the developed CS predictor framework. Aiming to facilitate this task, we mimic the traditional one-dimensional (1D) and 2D NMR spectra plots, which allow users to examine and differentiate signals within the same plot. To illustrate their utility, we focus on the β_2_AR, a GPCR with a critical role in managing asthma and respiratory disorders through bronchodilation, mucociliary clearance and anti-inflammatory effects^36^. Specifically, we examine the Cα and NH atoms of the backbone of two distinct aspartates (Fig. 5a). One is situated in the helical region of TM2 at the functionally highly relevant allosteric sodium binding site (D79^2×50^) and the other is situated in extracellular loop 2 (D192^45×51^), known to be involved in ligand binding in the β_2_AR^37^. The differences in location and chemical environment between these aspartate residues is reflected in different CS values (Fig. 5b,c). A more detailed explanation of these visualization modes, including their importance and interpretation, is provided in Supplementary Note 1. GPCRmd-NMR also permits resolving such dynamic conformational plasticity on higher spectroscopic dimensions. Our portal is highly flexible for constructing any of such spectroscopic representations. The most commonly studied atoms in NMR protocols are available as predefined selections, including methionines (^13^Cε^1^Hε), isoleucines (^13^Cδ^1^Hδ), alanine (^13^Cβ^1^Hβ), leucine (^13^Cδ^1^Hδ) and valine (^13^Cγ^1^Hγ). Ultimately, the 2D CS plots are interactive, allowing users to select specific residues directly within the plot. Upon selection, the chosen residues are highlighted and zoomed in the GPCRmd viewer. In the streaming mode, users can track the simulation frame as it progresses, facilitating the correlation of CSs with specific frames of the MD simulation. In Supplementary Figs. 19–23, we provide an overview of the CS tool and the streaming CS mode.

**Extended Data Fig. 1:**
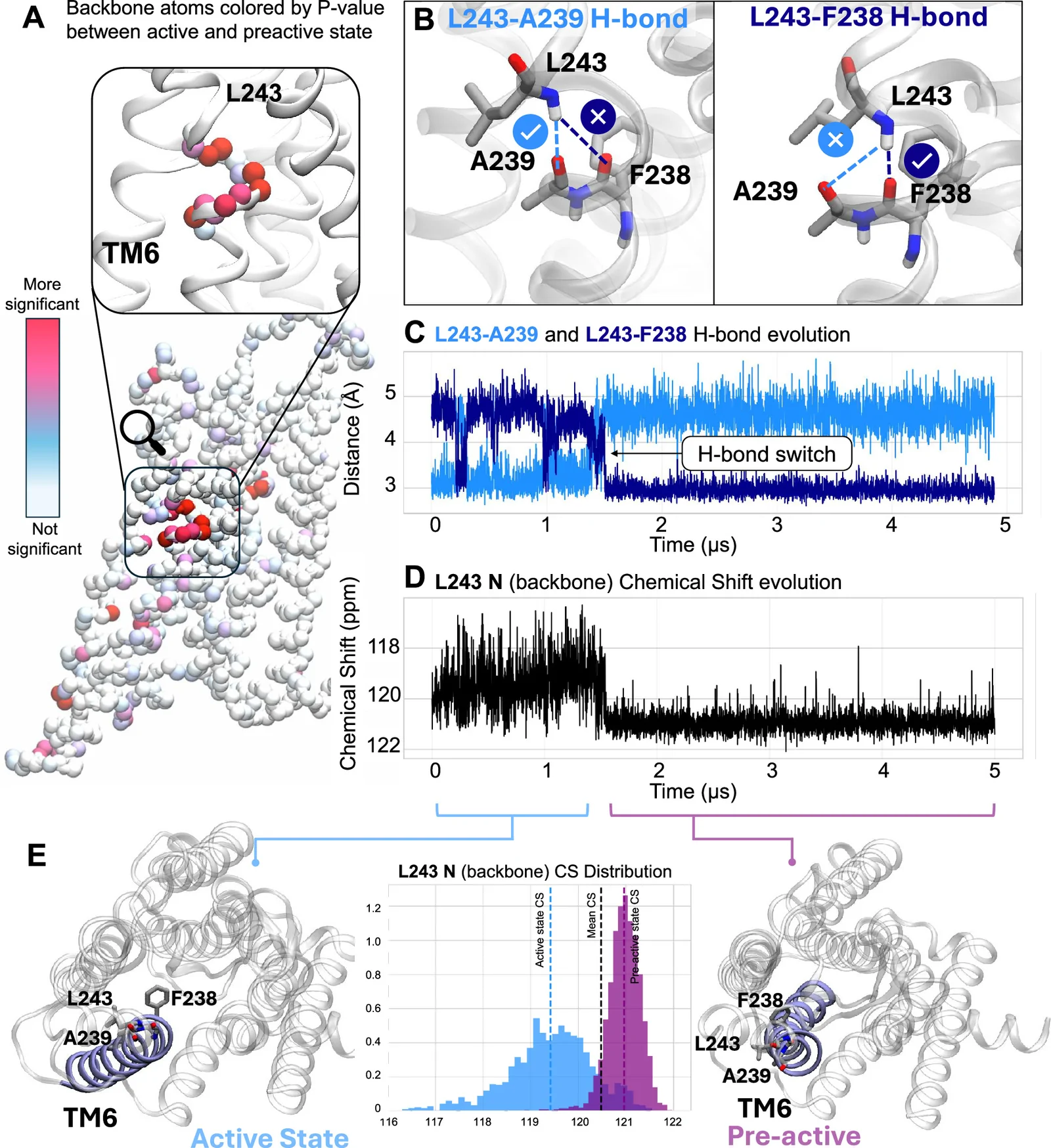
TM6 Hydrogen-Bond rearrangement from active to preactive transition for the A2A receptor. (**A**) C’ CA and N backbone atoms colored by the statistical significance (P value) of CS differences between active (first 1 µs) and preactive ensemble (last 3 µs), highlighting a strong correlation at the middle of TM6. (**B**) Structural snapshots illustrate the rearrangement of the TM6 hydrogen-bond network: in the active conformation, L243 forms a hydrogen bond with A239, whereas in the preactive state this interaction is replaced by a L243–F238 hydrogen bond. (**C**) Time evolution of the L243–A239 and L243–F238 hydrogen-bond distances (switch happens at ~1.5 μs). (**D**) Predicted backbone-NH chemical shift of L243, showing a marked change coincident with the hydrogen-bond transition. The RMSE of the CS predictor for the NH atom is 1.11ppm, lower than the change occurring at 1.4µs. (**E**) Representative active and preactive receptor conformations with TM6 highlighted. MD simulations were taken from D’Amore et al.^40^. The chemical shift (CS) distribution of the L243 backbone N atom is shown in blue for the first 1.4 μs of simulation and in purple for the 1.8–5 μs interval. The mean values for the Active State (119.41 ppm), Pre-active state (120.96 ppm) and the overall distribution (120.43 ppm) which corresponds to a population mixture of 30% active and 70% preactive states.

Furthermore, GPCRmd-NMR supervises the strategic spectroscopic reporter selection. In any spectroscopic studies of protein dynamics, the selection of suitable reporters is of crucial importance. However, this is often achieved through human experiences obtained using costly trial-and-error processes. GPCRmd-NMR could explore a large set of spectroscopic reporters and provide a systematic evaluation over the inexhaustible options in the real-life setting. Taking the μ-opioid receptor (μOR), a target for most clinically and recreationally used opioids^38^, as an example (GPCRmd 199, PDB 5C1M, submitted to GPCRmd by Kapoor et al.^39^), we aimed to identify reporter positions with significant readouts for studying receptor (in)activation. Convergence of the (in)activation process for the μOR was achieved through adaptive sampling, as discussed below. In a first step, MD frames were categorized into active and inactive states on the basis of the receptor conformation (Methods and Fig. 5d) and backbone NH CS values were obtained for both states (Fig. 5e). As expected, we observe that large CS changes occur mainly in regions of known functional relevance (Fig. 5f). For example, alterations occur around the NPxxY motif in the lower TM7 (L331^7×48^, L335^7×52^, Y336^7×53^) (blue to red) because of (in)activation motions. This is also evident for residues in TM6 with pronounced differences in V285^6×40^ and I278^6×33^ or the intracellular loop 3 (ICL3) as a consequence of the TM6 inward and outward movement. Most importantly, GPCRmd-NMR also reveals additional regions within the receptor interior that exhibit substantial backbone CS changes (for example in the β_2_AR, Y326^7×53^), which are not apparent in static structures and may point to previously unexplored functionally relevant receptor areas. Our approach can be extended feasibly to other receptors, functional states, sites and spectroscopic reporters, thus paving the way for rational design of biophysical approaches for studying GPCR dynamics. In addition to capturing full inactivation events, GPCRmd-NMR is sensitive to partial inactivation transitions, further supporting its utility for reporter selection. Analysis of unbiased adenosine A_2A_ receptor simulations from D’Amore et al.^40^ revealed localized predicted CS differences between active and preactive states, with the strongest changes in TM6. Along this pathway, a rearrangement of the TM6 hydrogen-bonding network centered on L243 coincides with a marked shift in its predicted NH CS (Extended Data Fig. 1). This coupling between structural transitions and spectroscopic readouts illustrates how GPCRmd-NMR can pinpoint residues that serve as sensitive reporters of intermediate conformational states.

Lastly, GPCRmd-NMR integrates hypothesis generation and spectroscopic solutions. Recent computational studies demonstrated that we can monitor the behavior of entrance gateways with MD simulations in GPCRs^16^. These transient pockets form and disappear dynamically. In this case study, we explore the formation of entrance gateways by using predicted CS values on the rhodopsin receptor, a key receptor for dim-light vision^41^. To this end, we analyze an MD simulation (GPCRmd 872, PDB 5DYS) where we observe the spontaneous insertion of a membrane lipid’s aliphatic tail into the receptor, specifically between the upper regions of TM5 and TM6. This event occurs during the first 50 ns of the simulation, capturing the transition from an unbound lipid state to the moment when the lipid tail fully penetrates the receptor. We evaluated the correlation between the predicted CS of the residues near the entrance gateway and the distance between the Cα atoms of residues V204 and A272, defining the opening of the cavity (Fig. 5g). As observed in Fig. 5h,i, V204 displayed a strong correlation (*r* = 0.83) for its ^1^Hγ CS, an atom that can be easily labeled experimentally. The ^1^Hγ CS evolved from approximately 1.2 ppm in the initial simulation steps (cavity closed) to 0.4 ppm once the lipid was fully inserted. While the gateways frequently open and close, this finding could be a starting point for exploring potential applications of NMR experiments to detect and localize the formation of entrance gateways in protein structures.

### Bridging the MD–NMR gap at distinct timescales

**Fig. 6:**
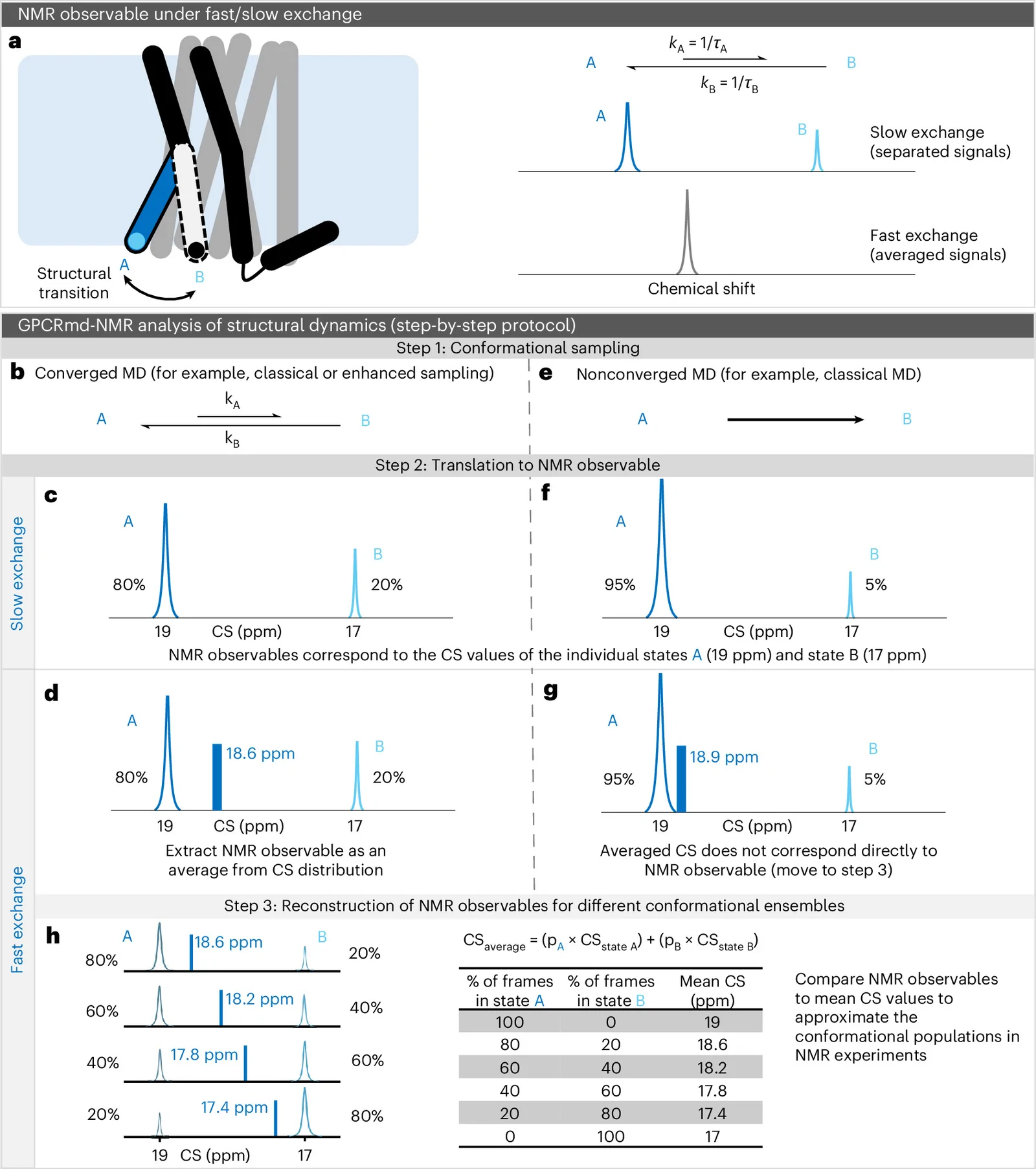
Bridging MD simulations and NMR observables across different timescales. **a**, The NMR signal depends on the rate of conformational exchange; slow exchange produces separate peaks for each state, whereas fast exchange yields a single population-weighted averaged signal. **b**, Converged MD achieves equilibrium sampling between conformational states A and B. **c**, NMR detects separate peaks corresponding to state-specific CSs. **d**, NMR observable approximated by averaging CS values across the equilibrated ensemble. **e**, Nonconverged MD between states A and B. **f**, Distinct NMR peaks still reflect individual state CS values. **g**, Averaged CS does not match the experimental NMR observable. **h**, Population-weighted reconstruction of NMR observables using state-specific CS values and estimated conformational populations.

It is well known that the timescale of conformation interconversion of proteins determines the NMR observable; when exchange occurs on a slow timescale, NMR spectra present individual signals for each state, whereas fast exchange results in a single population-weighted averaged signal^42^ (Fig. 6a and Supplementary Note 2). Beyond these complex spectroscopic behaviors, achieving equilibrium between conformational states, such as the inactive–active GPCR transition, on the millisecond-to-second timescales relevant to NMR experiments remains a major challenge for classical microsecond-scale MD simulations. Below, we outline complementary strategies using equilibrated and nonequilibrated MD simulations to address these challenges. Our approach follows a stepwise protocol (Fig. 6b–h): sampling transitions between states (step 1), translating computed CS values into NMR observables (step 2) and ultimately reconstructing NMR observables for distinct conformational ensembles (step 3).

#### Approximating NMR observables from converged MD simulations

Efficient conformational exploration is essential to capture the equilibrium distribution of GPCR states and their associated CS signals (step 1, Fig. 6b). When conformational transitions occur on timescales accessible to classical MD simulations, unbiased simulations are sufficient to approximate equilibrium conditions, as illustrated by the methionine side-chain switching observed in NTS_1_R (Fig. 3). However, some functionally relevant GPCR transitions, such as interconversion between inactive and active receptor states, require enhanced sampling strategies to achieve equilibrium sampling. A wide range of such methods have been developed by the MD community^43^. Here, we highlight adaptive sampling as a particularly effective approach. Adaptive sampling launches successive simulations on the basis of prior runs to enhance exploration of undersampled inactive and active conformations. Using the μOR and MD data generated by the Filizola lab^39^ as an example, this strategy enables the capture of full activation transitions and the separation of well-defined inactive and active conformational ensembles (Fig. 5d–f). Once equilibrium sampling is achieved, equilibrated MD simulations provide a direct route to approximating experimentally observable NMR signals, as state populations emerge naturally from the trajectory. The appropriate mapping of computed CS values to NMR observables depends on the rate of interconversion between conformational states on the NMR timescale. When interconversion occurs in the slow-exchange regime (for example, frozen samples), CS values computed for each individual conformational state can be directly assigned to distinct spectral peaks (step 2, Fig. 6c). This is also exemplified in our μOR case study, where CS values computed separately for inactive and active ensembles reveal residues with pronounced state-dependent CS differences (Fig. 5d–f). When interconversion is fast on the NMR timescale (for example, under solution NMR conditions), the observed signal corresponds to a single population-weighted average. In this case, equilibrated MD simulations allow CS values to be computed directly as ensemble averages over the full conformational distribution (step 2, Fig. 6d). This is also illustrated by the presented methionine switching in NTS_1_R (Fig. 3), where the averaged CS derived from equilibrated MD closely matches the experimentally observed single resonance.

#### Approximating NMR observables from nonconverged MD simulations

This requires capturing state transitions in nonconverged MD trajectories (step 1, Fig. 6e). NMR studies have reported that TM rearrangements associated with preactivation and activation occur on submillisecond timescales^34,44^. Of note, it has been shown that the actual transitions among the inactive, preactive and active conformational states themselves occur within the nanosecond-to-microsecond regime and are, therefore, well within the reach of classical MD simulations^16,40,45,46^ (Extended Data Fig. 1c,d and Supplementary Fig. 24). One commonly used approach to sample such rare but fast transitions is to initiate multiple short MD simulations from one starting conformation, enabling spontaneous GPCR activation or inactivation events to occur in some of the trajectories. This strategy, applied in our recent publication^16^ (Supplementary Fig. 24c), forms part of the dataset for which CS signals have been computed in this study. In step 2, the calculated CSs are then mapped onto experimentally detectable NMR observables. When transitions between states are slow on the NMR timescale, spectra display distinct peaks corresponding to each conformational state (Fig. 6f). In this case, each peak can be directly associated with the CS values computed separately for the inactive and active ensembles. When the state exchange is fast, NMR detects a single population-averaged signal. However, the average CS signal from unconverged MD will not correspond to an NMR observable in most cases (Fig. 6g). In this case, the user can move to step 3 and construct the average CS for different conformational distributions (Fig. 6h).

#### NMR-guided reconstruction of conformational space using (un)converged MD simulations

In step 3 (Fig. 6h), the user can approximate the NMR observable for fast-exchange processes as a population-weighted mean of the state-specific CS values obtained in step 2 from either converged or unconverged MD simulations according to the following equation:

1 $${\mathrm{CS}}_{\mathrm{average}}={p}_{\mathrm{state\; A}}\times {\mathrm{CS}}_{\mathrm{state\; A}}+{p}_{\mathrm{state\; B}}\times {\mathrm{CS}}_{\mathrm{state\; B}}$$

where CS_average_ corresponds to the NMR observable under fast exchange and CS_state A_ and CS_state B_ represent the CS values of states A and B, respectively. The coefficients *p* indicate, at equilibrium, the population of relevant states.

The complete CS dataset, resolved as a function of the conformational ensemble (Fig. 6h), facilitates interpretation of NMR measurements by quantitatively linking observed signals to underlying structural populations. It also enables the explicit identification of otherwise hidden or transient conformational states that contribute to the measured averages.

## Discussion

The decoding of complex protein dynamics is a long-lasting challenge in protein science. This task is particularly relevant for understanding the molecular mechanisms of GPCRs, as the cellular behaviors of these important drug targets depend strongly on the conformational space sampled by their distinct functional states. To tackle such a challenge, we developed an approach integrating in silico simulations and high-resolution experimental biophysical data at the 3D GPCRome level. Built on a community-driven, open-access GPCRmd platform and showcased on several receptors, our toolkit provides insights into GPCR dynamics and enhances their study from multiple perspectives.

Firstly, GPCRmd-NMR improves the biophysical data interpretability and functional state assignment. A major practical hurdle in the NMR studies of protein dynamics is the data interpretation. CSs, though highly sensitive to protein structure and environment, are difficult to directly translate into structural parameters. Many pioneering works have targeted this challenge, yet it becomes pronounced in highly dynamic proteins such as GPCRs^4^. GPCRmd-NMR enables the dynamic streamlining of CS evolutions at high temporal resolution. As showcased on the B1R–peptide complex (Fig. 2a), GPCRmd-NMR illuminates the complex conformational distribution together with the NMR data on a frozen sample. On the NTS_1_R at a more physiologically relevant temperature, our approach permits us to unravel the CS distribution averaged by the dynamic sampling of protein conformations (Fig. 3a–c). Such a capacity in interpreting the static and averaged NMR data on the basis of the time evolution of protein structures will open the way for uncovering the functional states encoding these biophysical readouts. Despite the success of many NMR reporters (for example, ^19^F-containing groups, ^13^C-methyl groups and backbone ^1^H–^15^N amide groups) in GPCR studies, they suffer from the limited information content (monoplexing approach) and/or the lack of correlation information between different sites (even in multiplexing methods). GPCRmd-NMR comprehends these valuable experimental biophysical data by ‘projecting’ them back to the fully atomistic protein dynamics. This approach was benchmarked against all available experimental NMR datasets from the BMRB, including CS datasets comprising 679 measurements.

Secondly, GPCRmd-NMR illuminates underexplored GPCR dynamics through the MD ‘mining’ of NMR data. As discussed above, the experimental biophysical data capture only some local or low-dimensional structural features. As demonstrated in the B1R–peptide case (Fig. 2a), our MD interpretation of the NMR readouts revealed complex structural plasticity that cannot be derived feasibly by conventional static NMR data analysis approaches alone. This capacity to illuminate the hidden structural dynamics became even more evident on NTS_1_, where a local side-chain conformational switch located next to the receptor activation site could be unmasked by the GPCRmd-NMR analysis of the experimental CS values (Fig. 3a–c). In this sense, our GPCRmd-NMR toolkit provides opportunities for identifying the previously unnoticed function-relevant sites by mining the experimental biophysical data.

Thirdly, the GPCRmd-NMR platform accelerates the generation of hypothesis-driven biophysical strategies for mapping function-relevant receptor dynamics. A prerequisite for the success of biophysical studies of receptor dynamics is the proper selection of the types and the specific locations of reporters. Traditionally, these biophysical reporters have been chosen on the basis of prior experience or through laborious trial-and-error processes, presenting a major bottleneck of the biophysical studies of receptor dynamics. As shown on rhodopsin, GPCRmd-NMR can generate function-relevant structural hypotheses (for example, transient cryptic channels involved in ligand entry) and bootstrap potential spectroscopic solutions by exploring the structural evolution of receptors (Fig. 5g–i). This feature not only greatly reduces the cost and risk of the biophysical studies but also guides the design of biophysical strategies to probe previously underexplored functional sites. Additionally, GPCRmd-NMR establishes in silico biophysical profiles across the 3D GPCRome, providing baseline patterns that facilitate the selection of receptor-specific biophysical reporters that map the unique structural features of each receptor.

Fourthly, GPCRmd-NMR opens a window to bridge the MD–NMR gap at distinct timescales. NMR spectroscopy has a wide timescale coverage (ps to day), while classical MD faces the challenge in accessing long-term structural evolution (ms timescale). The GPCRmd-NMR framework addresses this issue through multiple complementary approaches (Fig. 6). On the one hand, it leverages the recent finding that rare events, including large conformational transitions such as receptor activation and inactivation, are in fact rapid and can occur on the nanosecond-to-microsecond timescale^16,45,46^ (Supplementary Fig. 24a–c). These intriguing and widely observed MD behaviors across the GPCRome align well with the classical state transition model used for depicting the exchange process in NMR spectroscopy, in which the state transition rates are defined by the lifetime of states and rareness of events rather than the time taken by the state transition event^42^. On the other hand, GPCRmd-NMR addresses a key limitation in describing equilibrium ensembles and their underlying conformational heterogeneity by combining MD-derived conformational insights with experimental NMR observables. This integrative approach enables the NMR-guided reconstruction of the full conformational space (Fig. 6a–h).

Fifthly, beyond establishing an overall MD–NMR integrative framework, the generated GPCRmd-NMR dataset with its broad coverage of different GPCR types (Fig. 1b) provides researchers with a unique opportunity to interrogate specific mechanistic aspects of their GPCR of interest. As shown in this study, this includes the identification of transient ligand and lipid entry gateways and their coupling to spectroscopic reporters, quantification of orthosteric pocket plasticity and its impact on ligand recognition. The framework further enables characterization of fast side-chain exchange processes at key activation hinges, dissection of microswitch coupling during receptor (in)activation and analysis of disorder-to-order transitions in IDRs, such as C-terminal tails that regulate signaling interfaces. The exploration of conformational events linked to other functional aspects can be facilitated by our open-accessible protocol templates provided at GitHub (https://github.com/GPCRmd/GPCR_Chemical_Shift). Importantly, the broad coverage of the GPCRome in the current dataset also enables systematic investigation of how sequence variation shapes receptor dynamics and signaling mechanisms, providing comparative insights into the principles governing GPCR function. Beyond the current dataset, GPCRmd-NMR is constantly growing and will rapidly expand the open and transparent NMR data access to multiple functionally relevant aspects.

Lastly, GPCRmd-NMR presents a template for the open-access integration of MD simulations and diverse experimental biophysical data. As discussed, GPCRmd-NMR boosts the NMR studies of GPCR dynamics across the entire research pipeline, from hypothesis generation and reporter selection to data interpretation. Furthermore, its open database architecture permits incorporation of emerging deep learning-based CS predictors and quantum-chemical level CS calculations, as well as other deeply used CS predictors when requested by the community (that is, CAMShift^47^). Together with the more vigorous sampling of conformational space, they could resolve some remaining limits raised by the currently implemented CS predictors, for example, the relatively poor ^1^H CS performance. A current limitation of experimental validation is the limited availability of GPCR NMR datasets. At present, five GPCR backbone CS datasets (679 measurements) are publicly available in the BMRB, all of which are included in our validation study. Thanks to the high spatiotemporal resolution of fully atomistic MD at the backend of our platform, the demonstrated success on supporting NMR spectroscopy can be expanded to many other spectroscopic methods (for example, double electron–electron resonance, smFRET, ultraviolet–visible light spectroscopy and fluorescence), biophysical parameters (for example, distance, wavelength and signal intensity) or even biochemical readouts (for example, H/D exchange).

**Extended Data Fig. 2:**
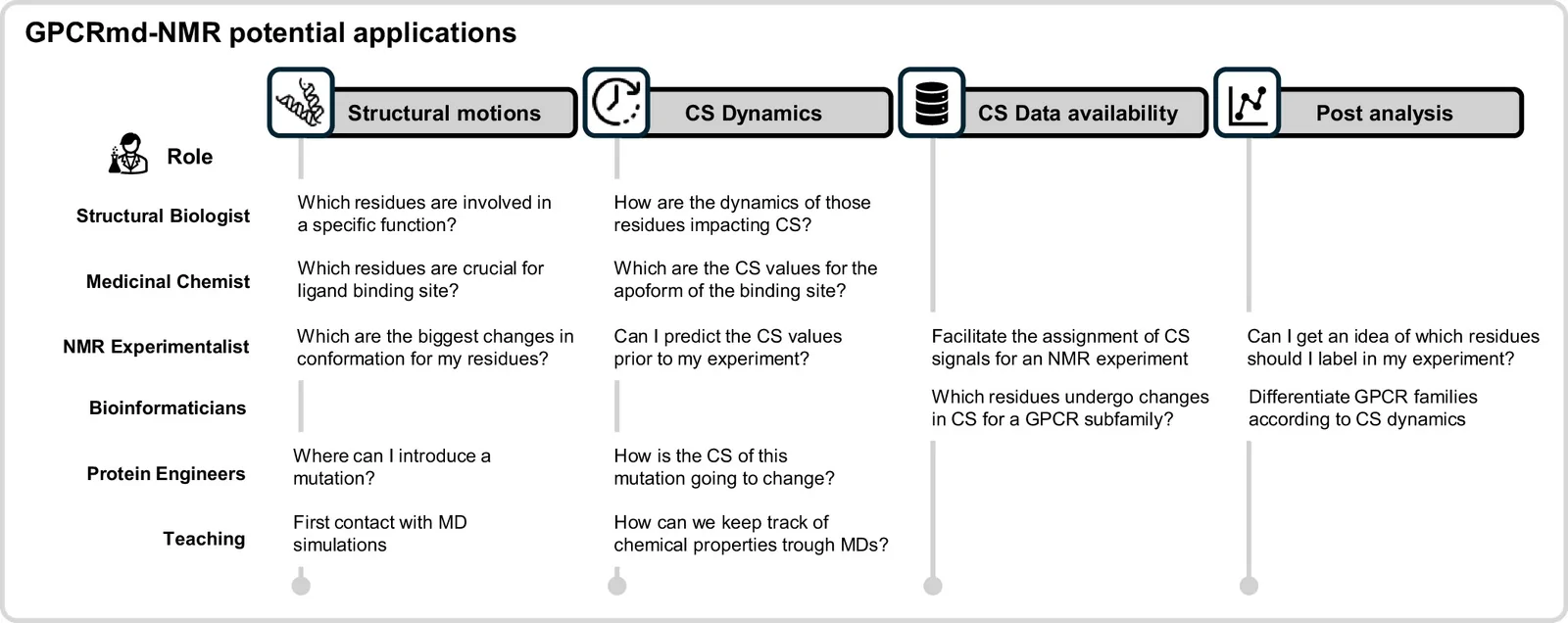
GPCRmd-NMR applications across disciplines. Schematic overview of how GPCRmd-NMR can support different stages of structure- and NMR-based analysis across user communities. Structural motions: MD simulations can identify residues involved in receptor function, ligand binding and conformational changes, guiding residue selection for mutational studies. CS dynamics: analysis of the relationship between residue conformational dynamics and CS, including estimation of CS values across receptor states and assessment of mutation-induced CS changes. CS data availability: support for the assignment and interpretation of NMR CS signals and identification of residues showing CS changes across GPCR subfamilies. Post-analysis: interpretation of experimental NMR data and identification of residues suitable for isotopic labelling.

In conclusion, GPCRmd-NMR is specifically tailored to meet the needs of scientists from diverse disciplines, such as medicinal chemists, NMR-based structural biologists and bioinformaticians among others (Extended Data Fig. 2). This broad accessibility makes the GPCRmd-NMR tool a valuable resource for advancing research across multiple directions in GPCR research. Moreover, this framework can serve as a template for similar efforts in other important protein families such as kinases, chaperones, nuclear receptors, ion channels and transporters.

## Methods

### Data processing for CS computation

The computing protocol is composed of an initial data preprocessing part, where nonprotein atoms and nonstandard amino acids are excluded from the prediction. Next, CS values are computed by a python pipeline and the binaries of the programs SHIFTX2 (version 1.10), SPARTA+ (version 2.90) and UCBShift (version 2.0). The pH and temperature parameters used by the predictors were set to 7 and 300 K, respectively. This is in line with the MD parameters used to run the simulations in GPCRmd. Afterward, the statistical error from the prediction is computed. For this, we calculated a block average analysis for each of the atoms included in the prediction. This blocking strategy allows us to treat data obtained from MD simulations as a sum of uncorrelated random variables, obtaining an accurate variance estimate for each predicted CS value. To ease the data comprehension, we provide the error as the sum of squares of the previously mentioned block average analysis and the intrinsic error of the predictor, defined by the authors^21,22^. For the 2D solution NMR plot, we represent the CS distributions by calculating the mean and deviation from the original 2D NMR data, aiming to mimic an average distribution of experimental solution NMR. Lastly, the data are rearranged into a user-friendly format ready to be downloaded from the server (example output in Supplementary Figs. 25 and 26). This process usually takes around 5–8 h of computing per system using our facilities and the code is scalable to any future server upgrades.

### Data visualization

The resulting CS predictions are stored in CSV format and they are used to produce the plots in the three different visualization modes. CSV format is easy to read and manipulate with most of the analysis tools available in the market. Two distinct CSV files are available for download from the server: a raw dataset and a simplified dataset. The raw dataset contains predicted CS values for each frame of the trajectory, while the simplified dataset includes the mean values and associated errors of these predictions. This difference was meant to avoid downloading unnecessarily large datasets. Each of the lines in both datasets represents a single atom of the system, including the information of its atom and residue identifier.

The interactive plots were generated by using the python libraries plotly (https://plotly.com/) and bokeh (https://bokeh.org/). When selecting any of the atoms shown in the plots, a zoomed-in representation appears in the GPCRmd viewer.

### MD simulations

CS values were computed for three specific case studies analyzed in this study, as well as for the entire GPCRmd repository.

#### Preparation of MD simulations for case studies

##### B1R study

For the B1R, modeling was not necessary as the structure was directly obtained from the Protein Data Bank (PDB 7EIB), whereas, for the NTS_1_, we used two different models, adapting each to the structure and models used in their corresponding NMR experiments.

##### NTS_1_R for Bumbak et al. study

For the work conducted by Bumbak et al., the initial structure of the NTS_1_R was based on PDB ID 6Z66, spanning from Y62 to N370. Using Prime from Maestro^48^, we modeled the ICL1 (K92–S97) and ECL2 (S214–G222). A homology model was subsequently generated using the target sequence outlined in the supplementary material of the authors’ paper, incorporating their key substitutions (M181L, M267L, M293L and M408V). An additional step of minimization of the loops was performed using Prime, with simultaneous minimization of protein hydrogen bonds to accommodate the modeled residues and prevent clashes.

##### NTS_1_R for Mohamadi et al. study

For the NTS_1_R case study, we used PDB 4BWB and no additional modeling was required.

##### β_2_AR for Heng et al. study

Simulations are available in the original paper.

##### Adenosine A_2A_ receptor for D’ Amore et al. study

Simulations are available in the original paper.

##### General system setup

All system setups were conducted using the CHARMM-GUI server. Protein cappings were achieved using methyl groups to neutralize them (ACE and CT3), while peptide cappings were intentionally left uncapped. Protonation states of amino acids were determined using PropKa (version 3.0)^49^ at a pH of 7. For the NTS_1_, a disulfide bridge was formed between C225 and C142. The receptors were embedded into a homogeneous bilayer composed of POPC after aligning the system with the OPM server. The systems were neutralized and ionized by adding appropriate concentrations of NaCl and MgCl_2_ to mimic experimental conditions. For the B1R, we used salt concentrations of 0.10 M NaCl and 0.05 M MgCl_2_; for the NTS_1_, we used 0.15 M NaCl.

The software used for MD simulations was ACEMD (version 3.3)^50^ using the CHARMM36m all-atom forcefield. Each system was subjected to energy minimization for 500 steps to alleviate steric clashes and optimize atomic positions. This was followed by a 20-ns equilibration phase where the system temperature was gradually increased from 0 to 300 K and then equilibrated in an NPT ensemble using a Berendsen barostat. To stabilize the system, restraints were applied to Cα atoms, gradually released from 1 kcal mol^−1^ to 0 kcal mol^−1^ over 15 ns, while restraints on remaining heavy atoms were relaxed from 0.1 kcal mol^−1^ to 0 kcal mol^−1^. The production runs were performed using a timestep of 4 fs under the NVT ensemble with a Langevin thermostat at a constant temperature of 300 K without restraints. The larger timescales, compared to the conventional 2 fs, were enabled by the use of hydrogen mass repartitioning, a technique that increases the mass of hydrogen atoms to allow increasing the timestep without compromising simulation accuracy. The duration of the production runs varied according to the experiment conducted:

- For the B1R study, three replicates of 500 ns were performed.
- For the NTS_1_R study conducted by Mohamadi et al., ten replicates of 1 µs were performed.
- For the NTS_1_R study conducted by Bumbak et al., three replicates of 1 µs were performed.

#### MD simulations from GPCRmd

The computational details of the MD simulations at GPCRmd are described on GitHub (https://github.com/GPCRmd/simulation_pipeline). In addition, the users can review the specific details for each simulation on the server in the simulation report section.

### Analysis and visualization

For CS analysis, simulations were processed using the SHIFTX2 CS predictor (along with SPARTA+ and UCBShift version 2.0 when specified). The specific pipelines used to compute and analyze the results for each of the experiments can be found below, along with a summary of the content for each section.

1. *GPCRmd-NMR aligns with experimental NMR observations—B1R:* The file was read from experimental DQ–SQ NMR and these values were converted into a grid. The predicted CS files were read, the values were transformed to DQ–SQ format and the two spectra were overlapped into a single plot, coloring each atom type differently. Black lines were added to identify correlated pairs of atoms.
2. *GPCRmd-NMR aligns with experimental NMR observations—NTSR:* The experimental data used in this study were sourced from BMRB 51907. We calculated the correlation between the experimental CS and the average value of the predicted CS obtained from the MD simulations for each replicate and for each CS predictor. This process was repeated for every atom type and the findings are presented in a tabular format. Additionally, we illustrate, using NGL representation^51,52^, the residues that contain experimental values.
3. *Illuminating underexplored GPCR dynamics* *through GPCRmd CS streaming:* In this study, we delved into receptor flexibility by analyzing predicted CSs. We construct a histogram comparing the CS of the HE atom from the methyl groups of M250.
4. *Exploring IDRs and interface contacts:* We aligned the published MD simulations and computed the correlation between coil/noncoil conformations and the minimum distance between the CT and the protein. We present the correlation of CS for each CT residue with respect to both features.
5. *Integrative enhancement of spectroscopic pipelines* *through GPCRmd-NMR—μOR:* In this study, we analyzed a community-contributed 1.2-µs adaptive sampling simulation capturing the (in)activation dynamics of the µOR while bound to the agonist morphine (GPCRmd 199, PDB 5C1M, submitted to GPCRmd by Kapoor et al.^39^). This simulation allowed us to explore the dynamic behavior and structural changes of this receptor during the activation process. To begin our analysis, we first read and aligned the MD trajectory frames uploaded in GPCRmd. We established as reference structures the inactive (PDB 4DKL) and active (PDB 5C1M) states of the receptor. These references served to categorize the frames with a root-mean-square deviation of 1.5 Å or less from the reference structures into two groups: active and inactive-like. We performed a Welch’s *t*-test to determine the significance of differences in CS values per residue. Lastly, we scaled and mapped the *P* values to *B* factors within a PDB file for visualization purposes. The resulting data allowed for the identification of the most variant residues between active and inactive forms, which could be readily visualized using software such as VMD^53^, using the *B* factor as a color scheme.
6. *Integrative enhancement of spectroscopic pipelines* *through GPCRmd-NMR—rhodopsin receptor:* We computed the distance between the Cα atoms of V204 and A272 and plotted the evolution of the CS of the V204 Hγ.
7. *Integrative enhancement of spectroscopic pipelines* *through GPCRmd-NMR—adenosine A*_*2A*_ *receptor:* MD trajectories were aligned and analyzed after retrieval from the original publication. Distances of the L243–A239 and L243–F238 hydrogen bonds were monitored, along with the CS evolution of the L243 NH atom.

### Reporting summary

Further information on research design is available in the Nature Portfolio Reporting Summary linked to this article.

## Online content

Any methods, additional references, Nature Portfolio reporting summaries, source data, extended data, supplementary information, acknowledgements, peer review information; details of author contributions and competing interests; and statements of data and code availability are available at https://doi.org/10.1038/s41594-026-01891-8.

## Supplementary information

Supplementary Information Supplementary Notes 1 and 2, Figs. 1–26, Tables 1 and 2 and References.

Reporting Summary

Peer Review File

## Source data

Source Data Fig. 2 Computational CS predictions for P8 B1R and correlations plots for NTS_1_R.

Source Data Fig. 3 Computational CS predictions for M250 on NTS_1_R.

Source Data Fig. 4 Computational CS predictions for CT of β_2_AR.

## Data Availability

The GPCRmd-NMR platform is available online through the GPCRmd resource (https://www.gpcrmd.org/gpcrmd-nmr-tool). All MD simulations used as case studies in this publication can be accessed using the indicated GPCRmd submission identifiers. Users can visualize simulations directly on the platform, calculate CSs and download both the simulation trajectories and the corresponding CS data. Original data underlying this paper are available in the main text and its Supplementary Information. Source data are provided with this paper.

## Code availability

To facilitate the reproduction of the experiments, we uploaded the Jupyter-notebook files to the GPCRmd GitHub repository (https://github.com/GPCRmd/GPCR_Chemical_Shift). These notebooks contain all the code, information and steps needed to replicate the results shown in this study. The user might need to download the data from the provided links in the Jupyter-notebook and place them into the proper directories because of the size of the files.
