# Supplementary material for: Integrative biophysical illumination of the 3D GPCRome dynamics
Source: Nat Struct Mol Biol. 2026 Oct 27;33(11):1733–44. doi: 10.1038/s41594-026-01891-8 (PMC13759331; doi:10.1038/s41594-026-01891-8)
Supplement: Supplementary file 1 [file 41594_2026_1891_MOESM1_ESM.pdf]

nature structural & molecular biology

Supplementary information https://doi.org/10.1038/s41594-026-01891-8

Integrative biophysical illumination of the 3D GPCRome dynamics

In the format provided by the authors and unedited

Supplementary Information

Content

1. Supplementary Notes
2. Supplementary Figures
3. Supplementary Tables
4. References

1. Supplementary Notes

Supplementary Note 1

The first mode, termed "One-Dimensional Mean Chemical Shift", presents a mean representation of the CS derived from the entire MD. The distribution's center corresponds to the mean of the CS, while its width represents an error estimation for the CS prediction (refer to methods for more details). This error estimation guides the user to distinguish whether two measures are significantly different or not. In **Figure 5B**, a visual representation illustrates how the two distributions for the Cα and Nitrogen atoms between the aspartates differ in CS. For instance, the Nitrogen atom and the Cα of $D79^{2.50}$ and $D192^{45x51}$ are well separated in the β_2 adrenergic receptor. The second mode, termed "Two-Dimensional Chemical Shift", allows users to correlate two atom types by mapping their distribution on a plane, resembling a real 2D-NMR plot. This mode helps to quickly understand the ranges of CS values explored throughout the simulation. **Figure 5C** illustrates the "Two-Dimensional Chemical Shift" mode, where $D79^{2.50}$ has slightly greater fluctuation in CS compared to $D192^{45x51}$ for the Cα and Nitrogen atoms. Importantly, both visualization modes are interactive to enhance user comprehension. For instance, selecting a specific NMR signal allows users to visualize the corresponding atom within the 3D receptor structure using the GPCRmd viewer. A snapshot from GPCRmd highlighting the main features of both modes is presented in **Supplementary Figures 19-23**.

Supplementary Note 2 - The intermittent conformational transition process

It has been frequently observed that the actual state transition processes, including the (pre) inactive-(pre)active conformation transitions, occur within the nanosecond to microsecond regime[1–4]. The observed fast state transitions in these cases support an intermittent conformation transition model, in which distinct states transit “instantly” between each other. This model is in particular valid in the slow exchange regime since the time required for the state transition event is much smaller than the lifetime of states. The long lifetime of states leads to the rareness of conformational changes and small exchange rate constant, which equivalently defines the so-called slow exchange in NMR spectroscopy. Here, our survey on MD data in GPCRMD database and literature data strongly shows a unified picture of slow exchange across in silico MD simulation and NMR spectroscopy, which is based on long-living states and rapid transition between these states. Therefore, a number of state resampling (for the equilibrated MD) and sampling (for the non-equilibrated MD) approaches can be taken to tackle the slow exchange regime as long as the transition events can be captured by MD simulations. For the fast exchange scenario, similar approaches can be taken in combination with observable averaging.

2. Supplementary Figures

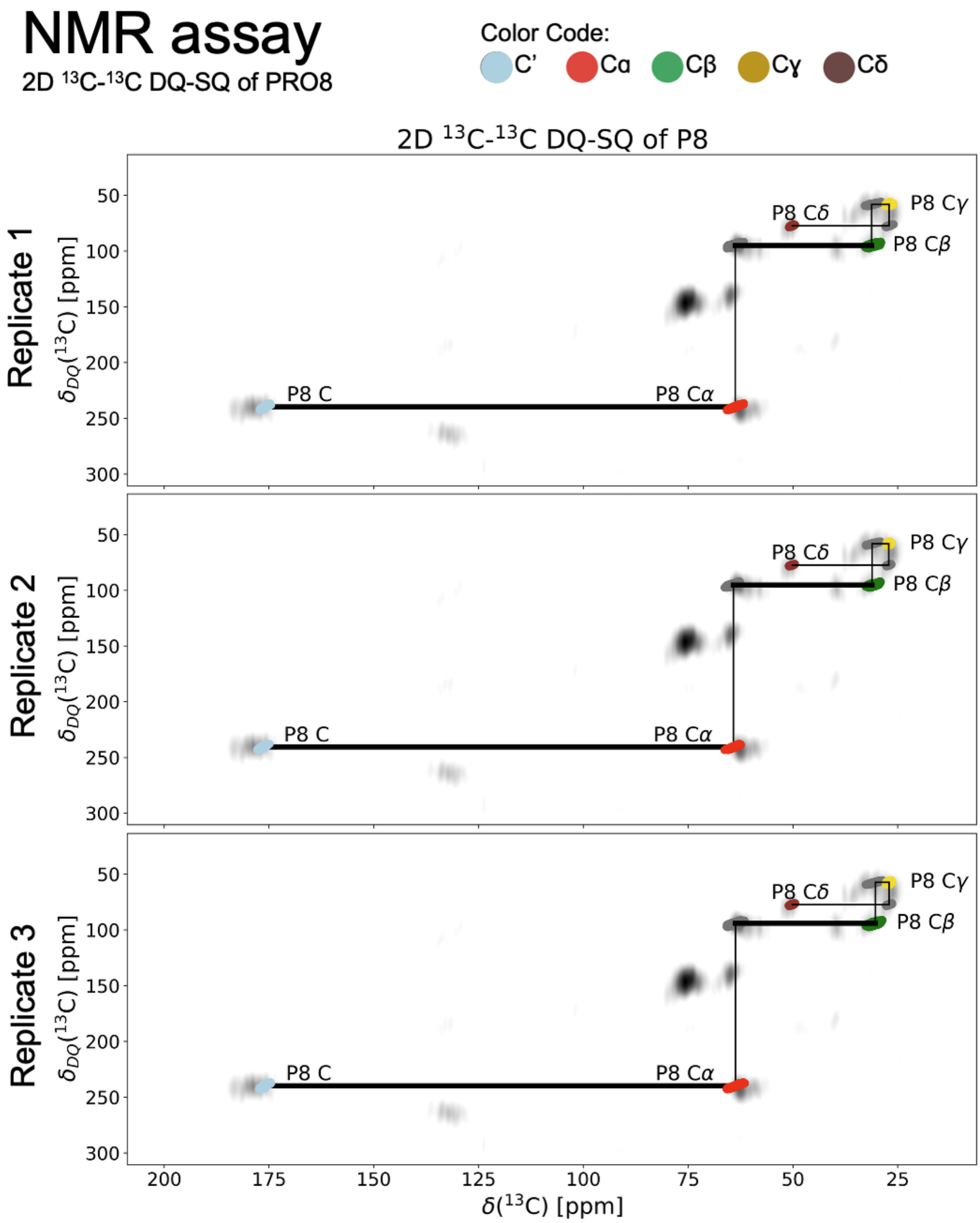

Supplementary Figure 1. ^{13}C-^{13}C DQ-SQ overlap between predicted (colored) and experimental (grey) chemical shift values for P8 in the Bradykinin 1 Receptor. The lines between atom types represent the carbon-carbon correlations, indicating spatial proximity between them. Each replicate consists in a independent MD simulation of 500ns (GPCRmd ID: 2095, PDB: 7EIB).

ShiftX2 – C’

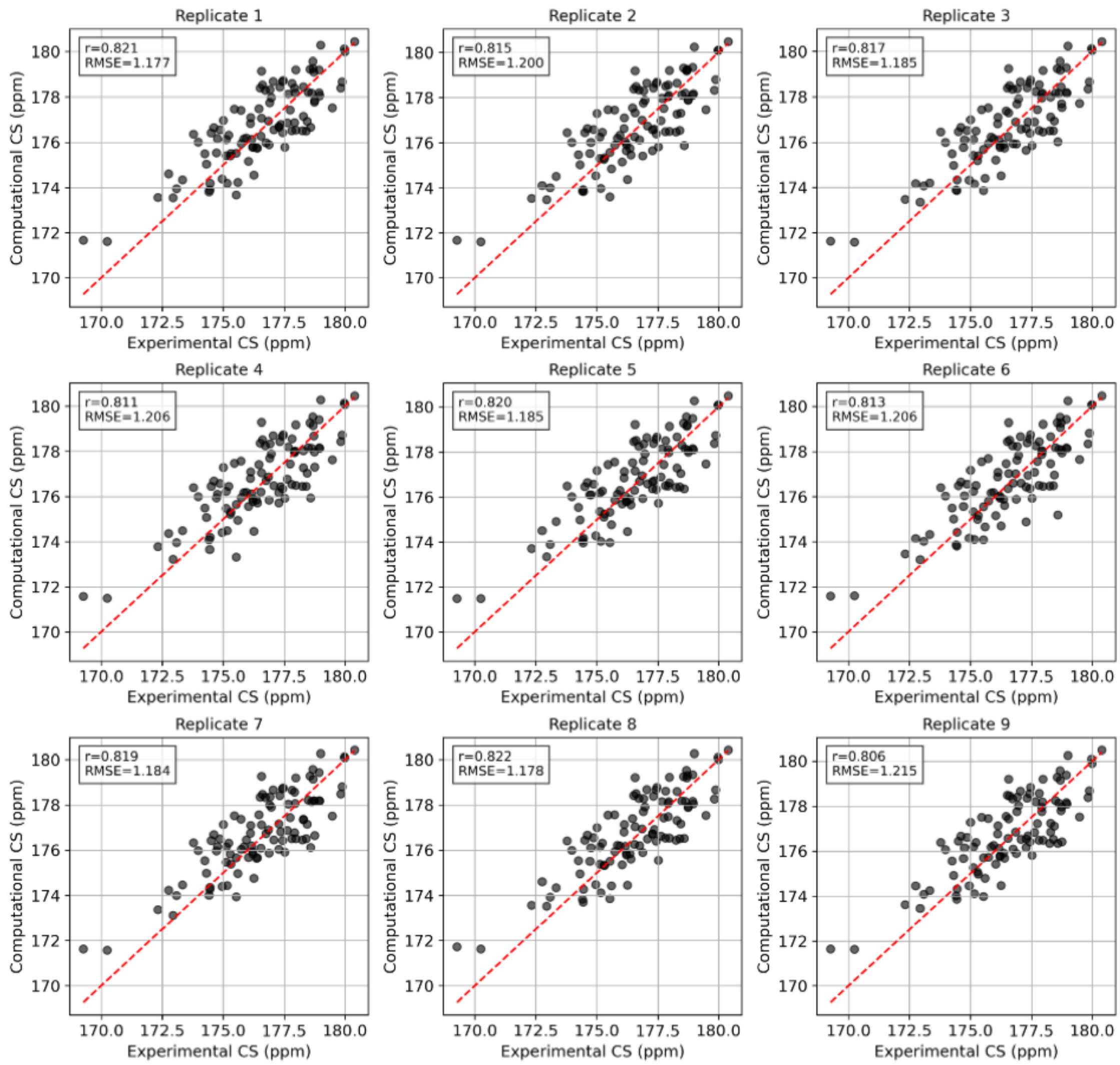

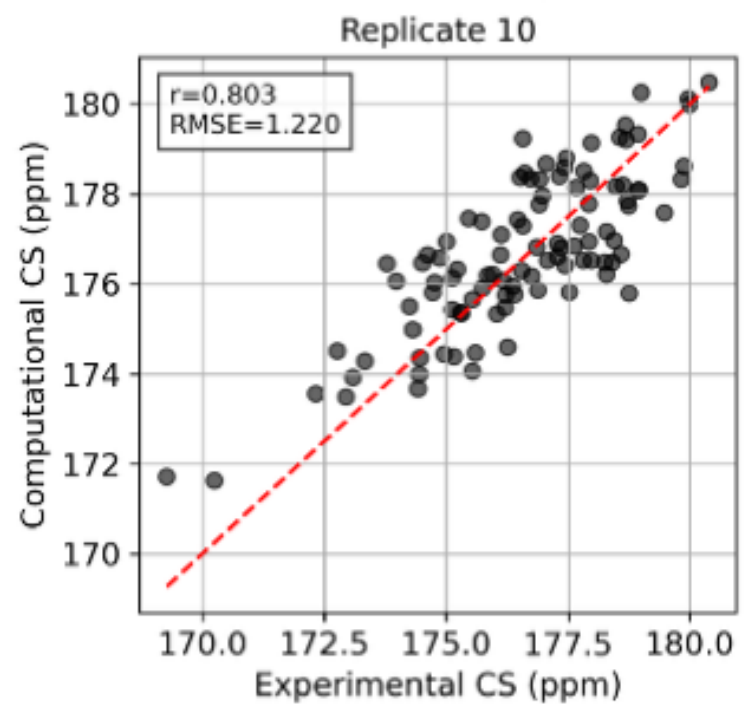

ShiftX2 - C'	Rep. 1	Rep. 2	Rep. 3	Rep. 4	Rep. 5	Rep. 6	Rep. 7	Rep. 8	Rep. 9	Rep. 10
Correlation coefficient	0.821	0.815	0.817	0.811	0.820	0.813	0.819	0.822	0.806	0.803
RMSE (ppm)	1.177	1.200	1.185	1.206	1.185	1.206	1.184	1.178	1.215	1.220

Supplementary Figure 2. ShiftX2 Correlations for atom type C’ (carboxyl) for the neurotensin-1 receptor (experiment ID 7 in Table 1).

ShiftX2 – CA

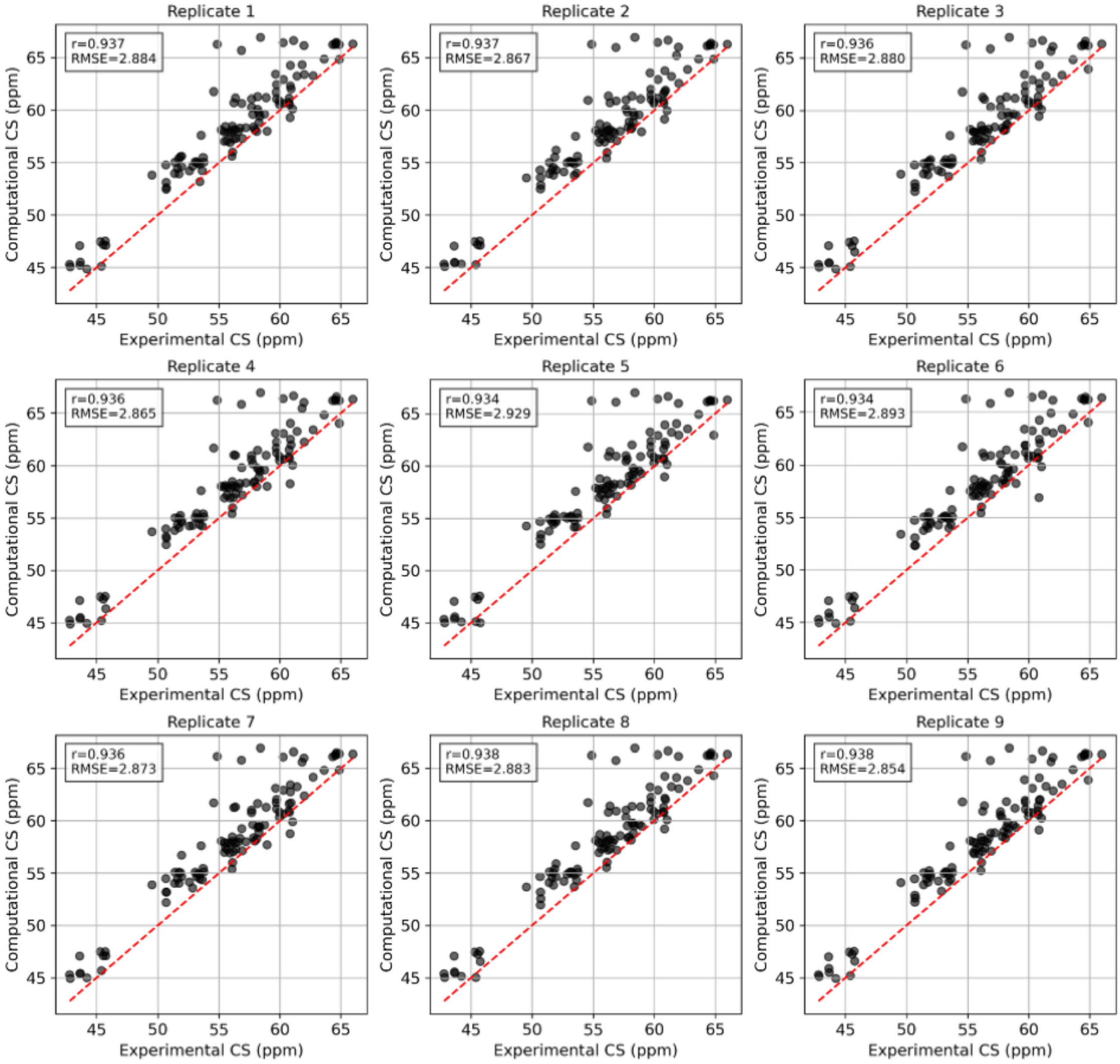

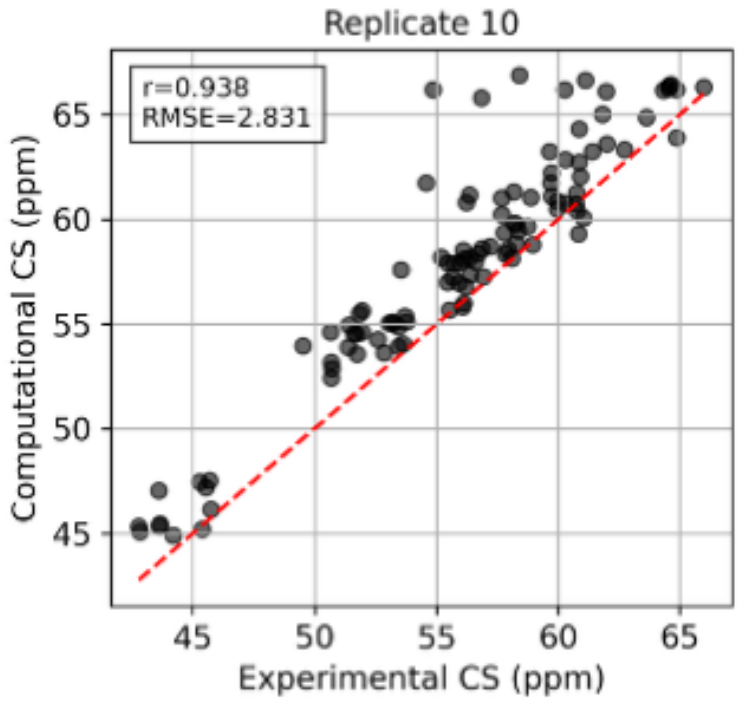

ShiftX2 - CA	Rep. 1	Rep. 2	Rep. 3	Rep. 4	Rep. 5	Rep. 6	Rep. 7	Rep. 8	Rep. 9	Rep. 10
Correlation coefficient	0.937	0.937	0.936	0.936	0.934	0.934	0.936	0.938	0.938	0.938
RMSE (ppm)	2.884	2.867	2.880	2.865	2.929	2.893	2.873	2.883	2.854	2.831

Supplementary Figure 3. ShiftX2 Correlations for atom type CA for the neurotensin-1 receptor (experiment ID 7 in Table 1).

ShiftX2 – CB

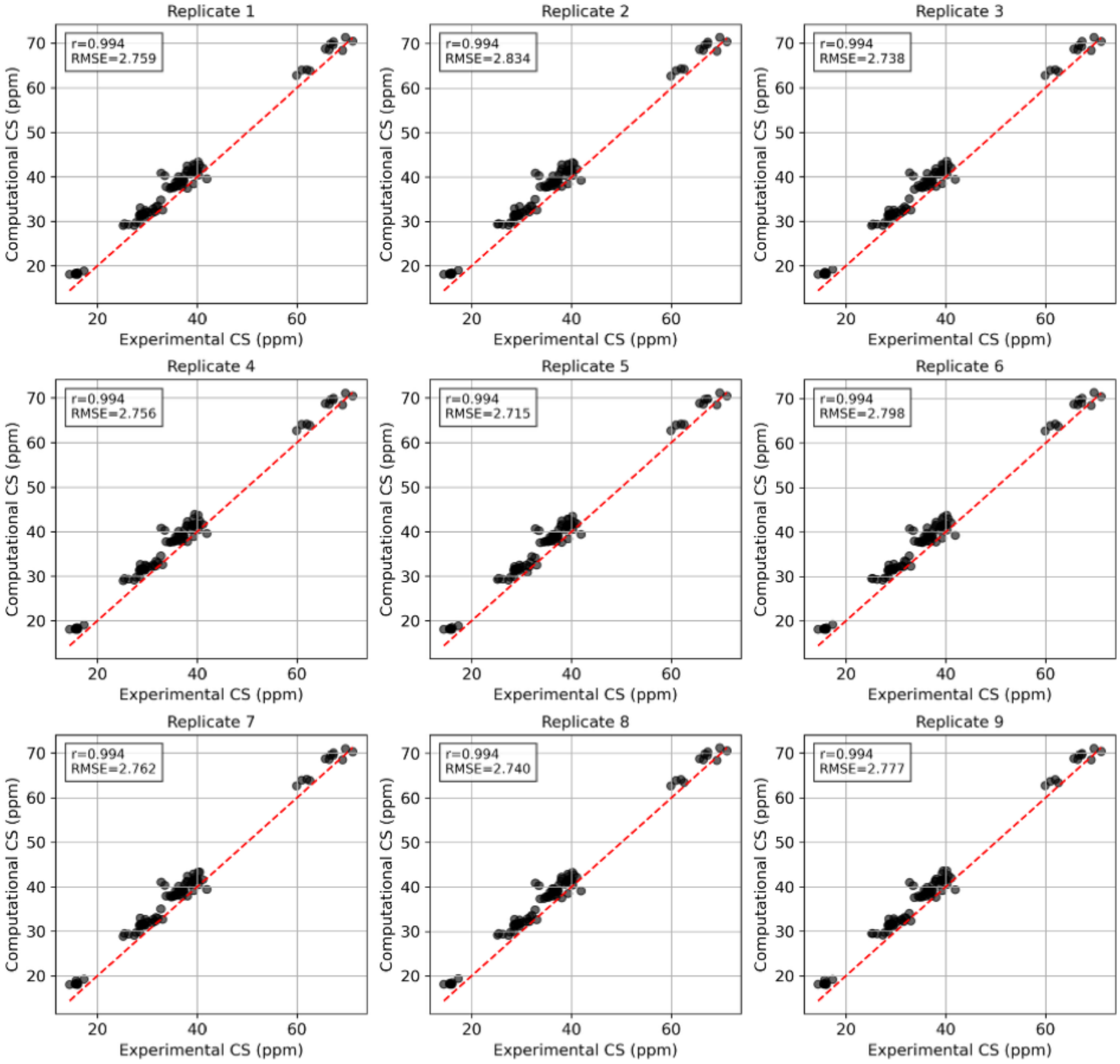

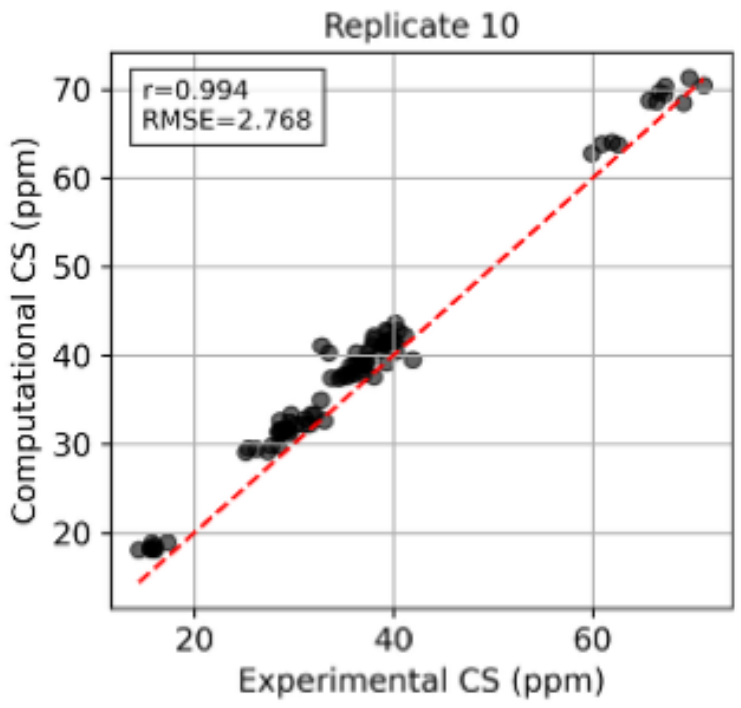

ShiftX2 - CB	Rep. 1	Rep. 2	Rep. 3	Rep. 4	Rep. 5	Rep. 6	Rep. 7	Rep. 8	Rep. 9	Rep. 10
Correlation coefficient	0.994	0.994	0.994	0.994	0.994	0.994	0.994	0.994	0.994	0.994
RMSE (ppm)	2.759	2.834	2.738	2.756	2.715	2.798	2.762	2.740	2.777	2.768

Supplementary Figure 4. ShiftX2 Correlations for atom type CB for the neurotensin-1 receptor (experiment ID 7 in Table 1).

ShiftX2 – NH

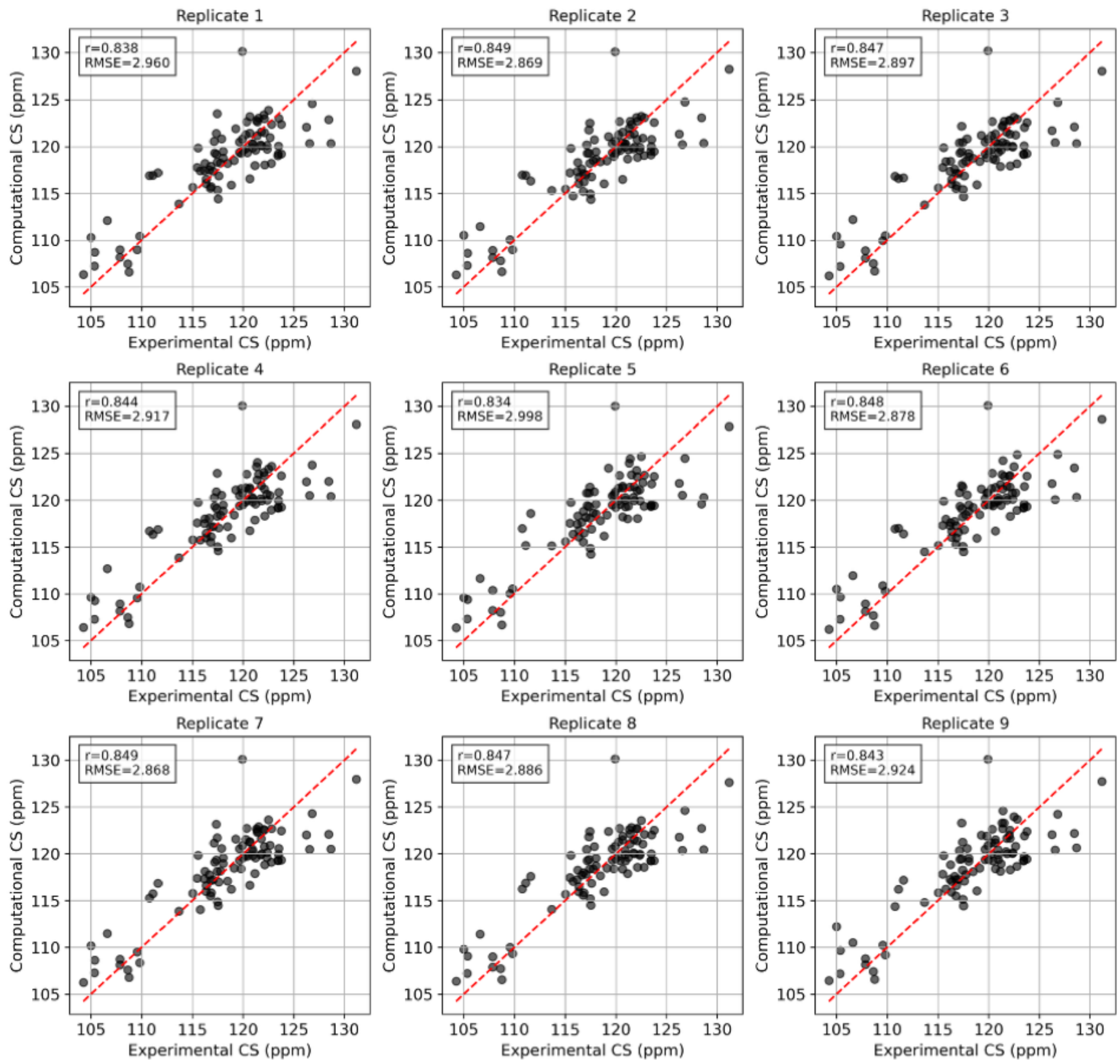

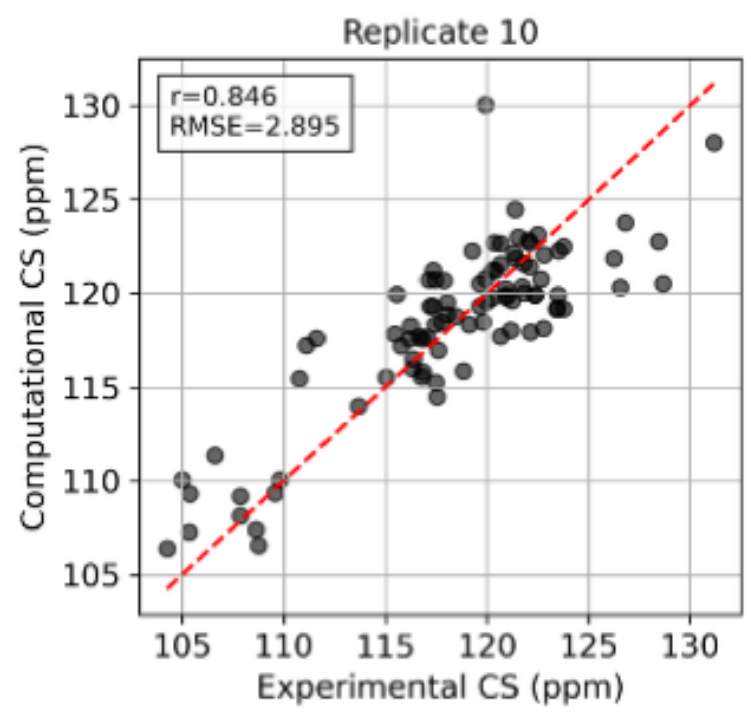

ShiftX2 - NH	Rep. 1	Rep. 2	Rep. 3	Rep. 4	Rep. 5	Rep. 6	Rep. 7	Rep. 8	Rep. 9	Rep. 10
Correlation coefficient	0.838	0.849	0.847	0.844	0.834	0.848	0.849	0.847	0.843	0.846
RMSE (ppm)	2.960	2.869	2.897	2.917	2.998	2.878	2.868	2.886	2.924	2.895

Supplementary Figure 5. ShiftX2 Correlations for atom type NH (backbone) for the neurotensin-1 receptor (experiment ID 7 in Table 1).

ShiftX2 – HN (backbone)

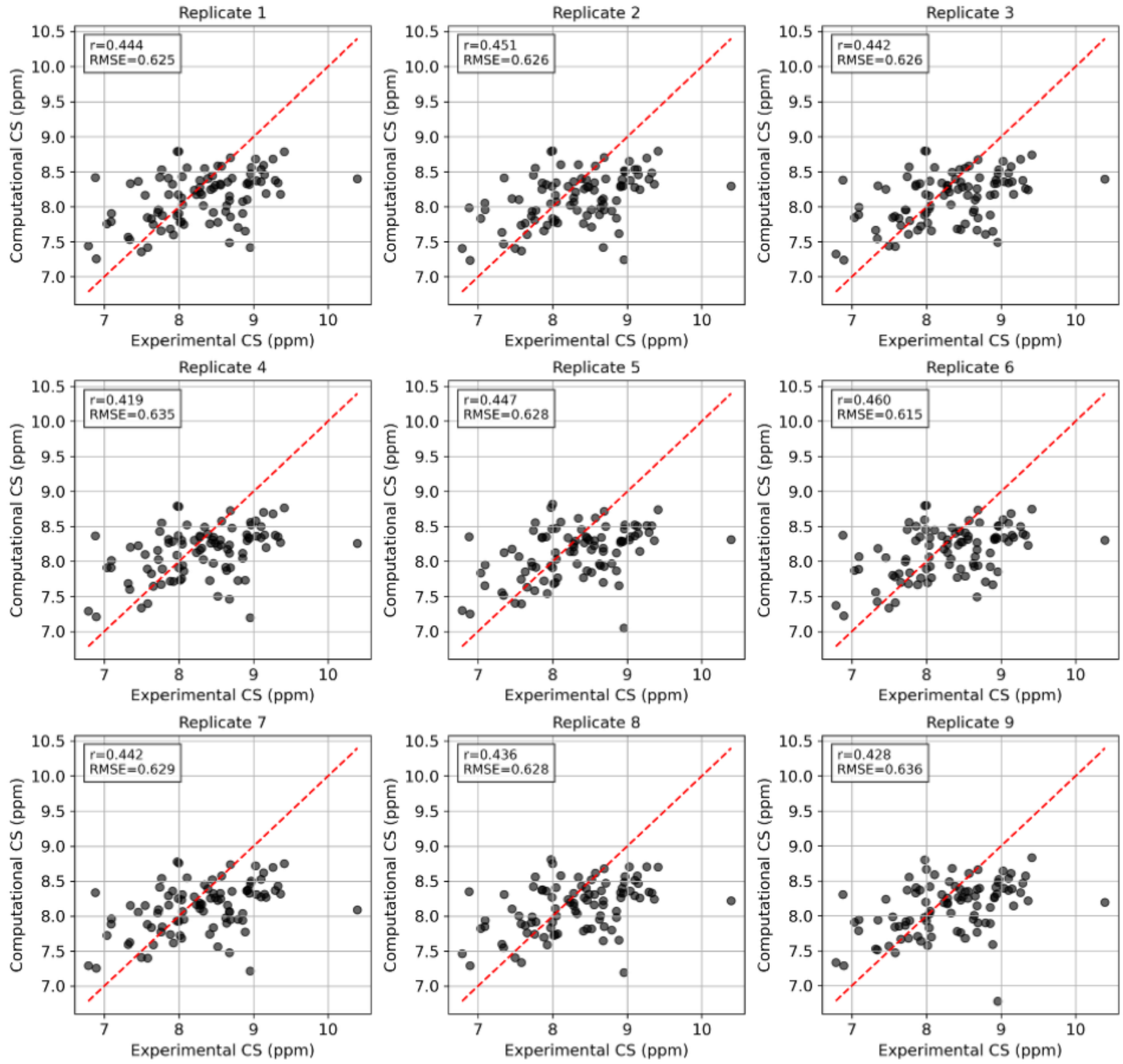

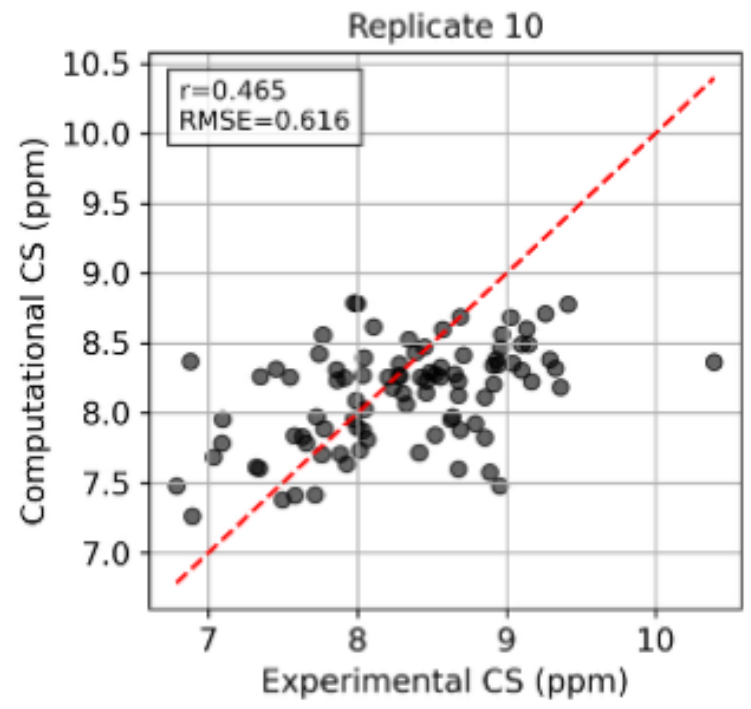

ShiftX2 - HN	Rep. 1	Rep. 2	Rep. 3	Rep. 4	Rep. 5	Rep. 6	Rep. 7	Rep. 8	Rep. 9	Rep. 10
Correlation coefficient	0.444	0.451	0.442	0.419	0.447	0.460	0.442	0.436	0.428	0.465
RMSE	0.625	0.626	0.626	0.635	0.628	0.615	0.629	0.628	0.636	0.616

Supplementary Figure 6. ShiftX2 Correlations for atom type HN (backbone) for the neurotensin-1 receptor (experiment ID 7 in Table 1).

Sparta+ – C’

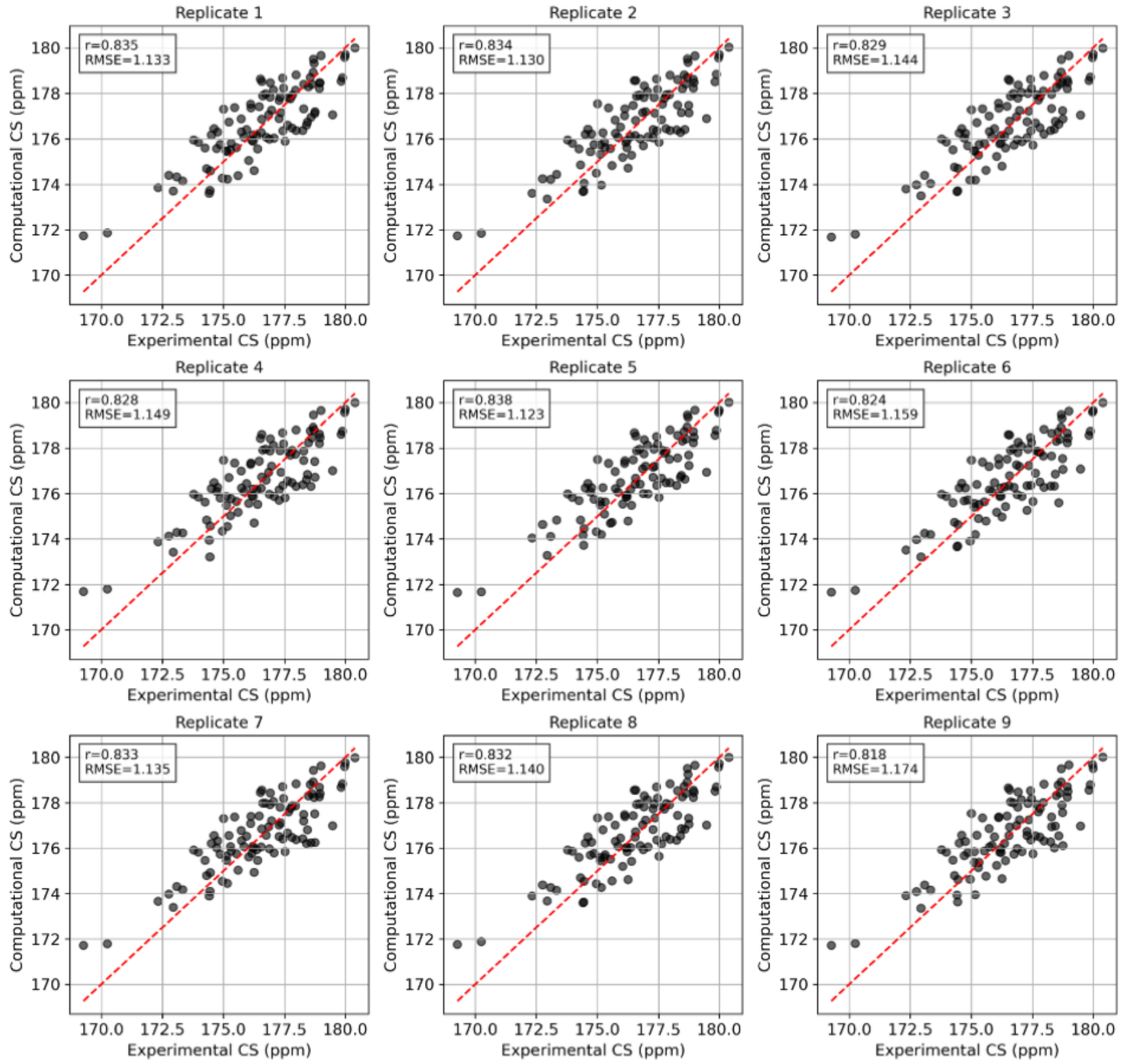

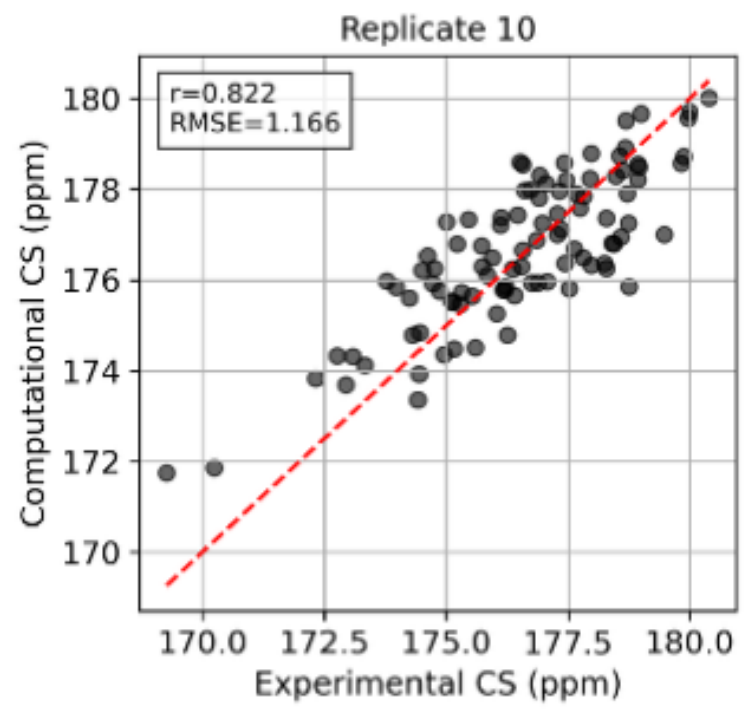

SPARTA+ - C'	Rep. 1	Rep. 2	Rep. 3	Rep. 4	Rep. 5	Rep. 6	Rep. 7	Rep. 8	Rep. 9	Rep. 10
Correlation coefficient	0.835	0.834	0.829	0.828	0.838	0.824	0.833	0.832	0.818	0.882
RMSE (ppm)	1.133	1.130	1.144	1.149	1.123	1.159	1.135	1.140	1.174	1.166

Supplementary Figure 7. Sparta+ Correlations for atom type C’ (carboxyl) for the neurotensin-1 receptor (experiment ID 7 in Table 1).

Sparta+ – CA

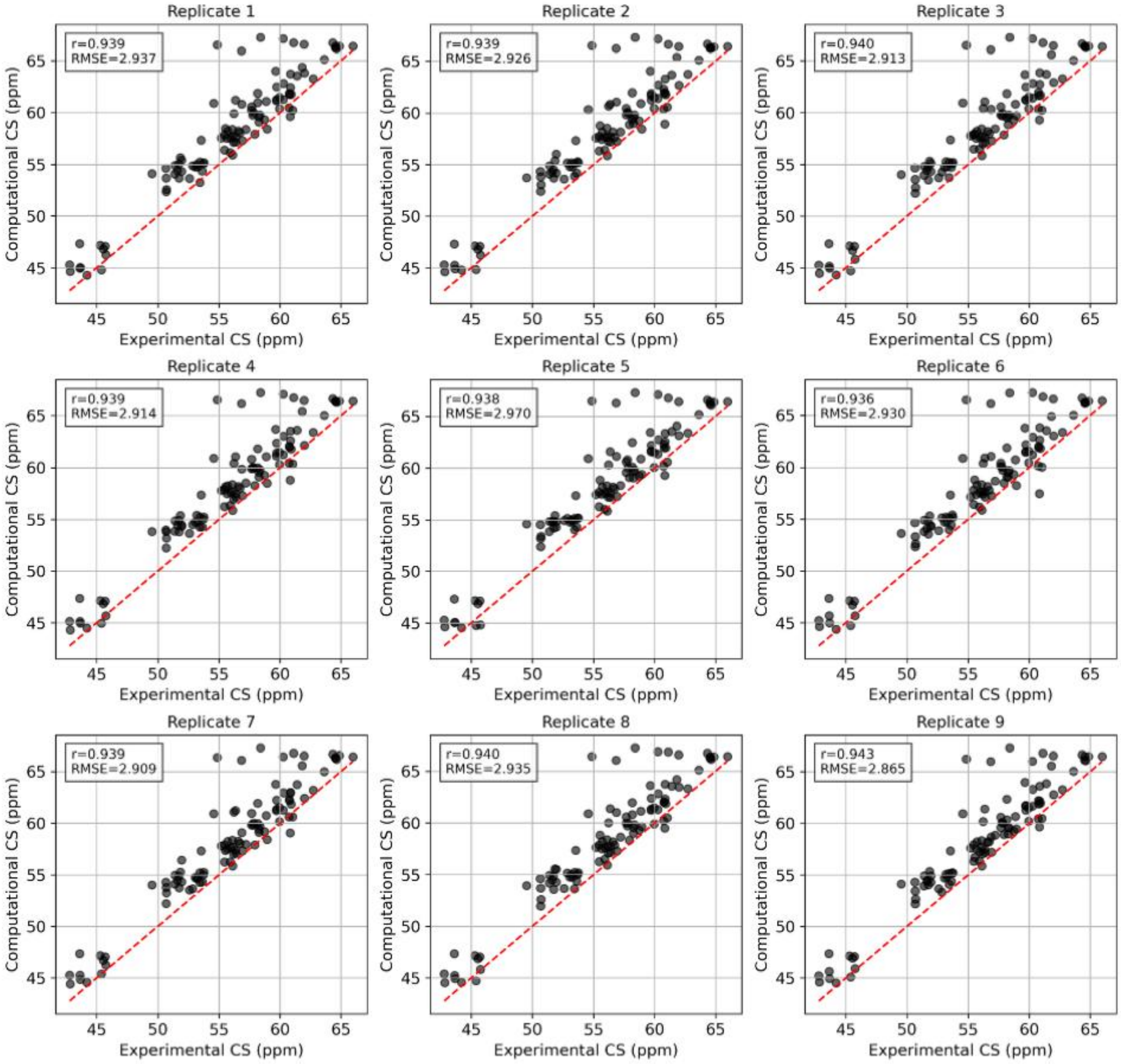

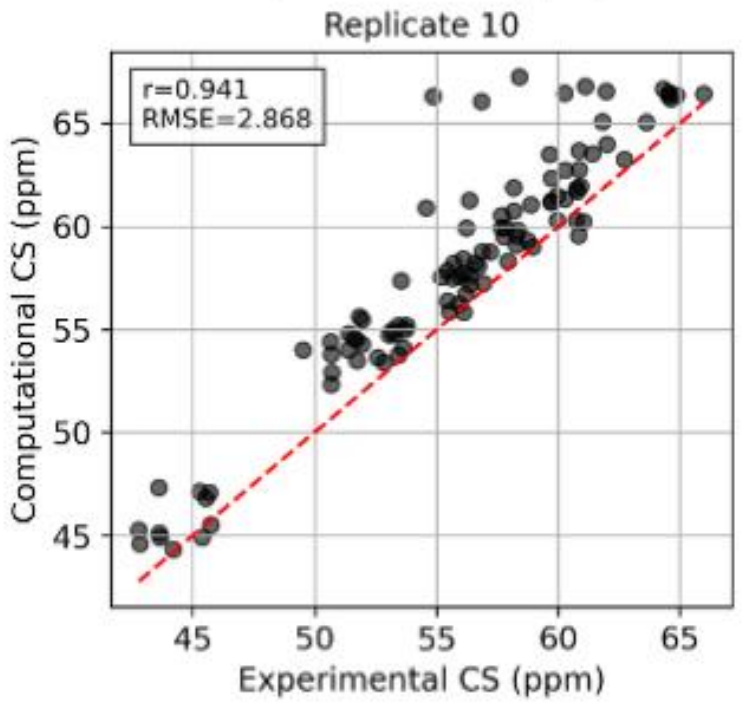

SPARTA+ - CA	Rep. 1	Rep. 2	Rep. 3	Rep. 4	Rep. 5	Rep. 6	Rep. 7	Rep. 8	Rep. 9	Rep. 10
Correlation coefficient	0.939	0.939	0.940	0.939	0.938	0.936	0.939	0.940	0.943	0.941
RMSE (ppm)	2.937	2.926	2.913	2.914	2.970	2.930	2.909	2.935	2.865	2.868

Supplementary Figure 8. Sparta+ Correlations for atom type CA for the neurotensin-1 receptor (experiment ID 7 in Table 1).

Sparta+ – CB

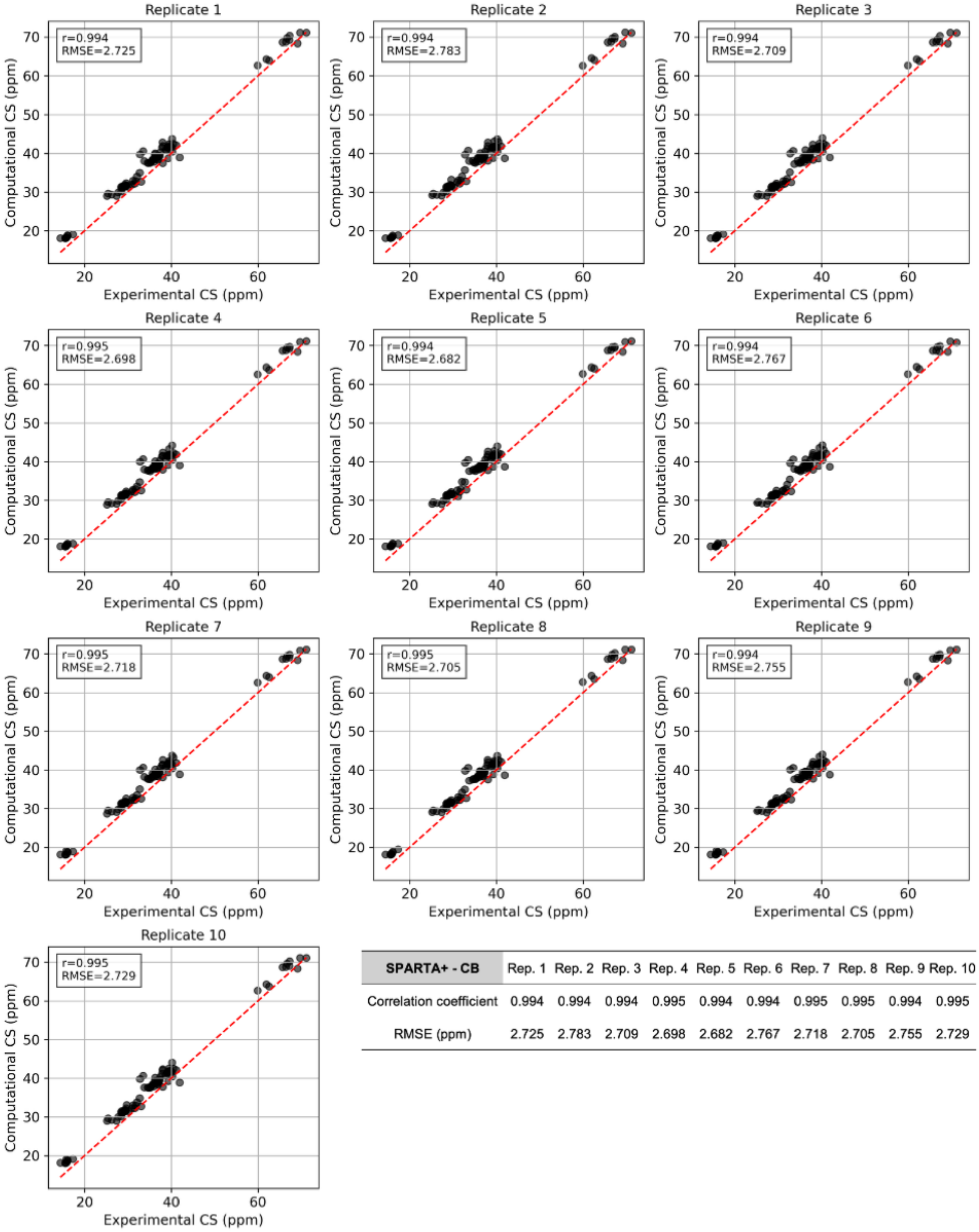

SPARTA+ - CB	Rep. 1	Rep. 2	Rep. 3	Rep. 4	Rep. 5	Rep. 6	Rep. 7	Rep. 8	Rep. 9	Rep. 10
Correlation coefficient	0.994	0.994	0.994	0.995	0.994	0.994	0.995	0.995	0.994	0.995
RMSE (ppm)	2.725	2.783	2.709	2.698	2.682	2.767	2.718	2.705	2.755	2.729

Supplementary Figure 9. Sparta+ Correlations for atom type CB for the neurotensin-1 receptor (experiment ID 7 in Table 1).

Sparta+ – NH

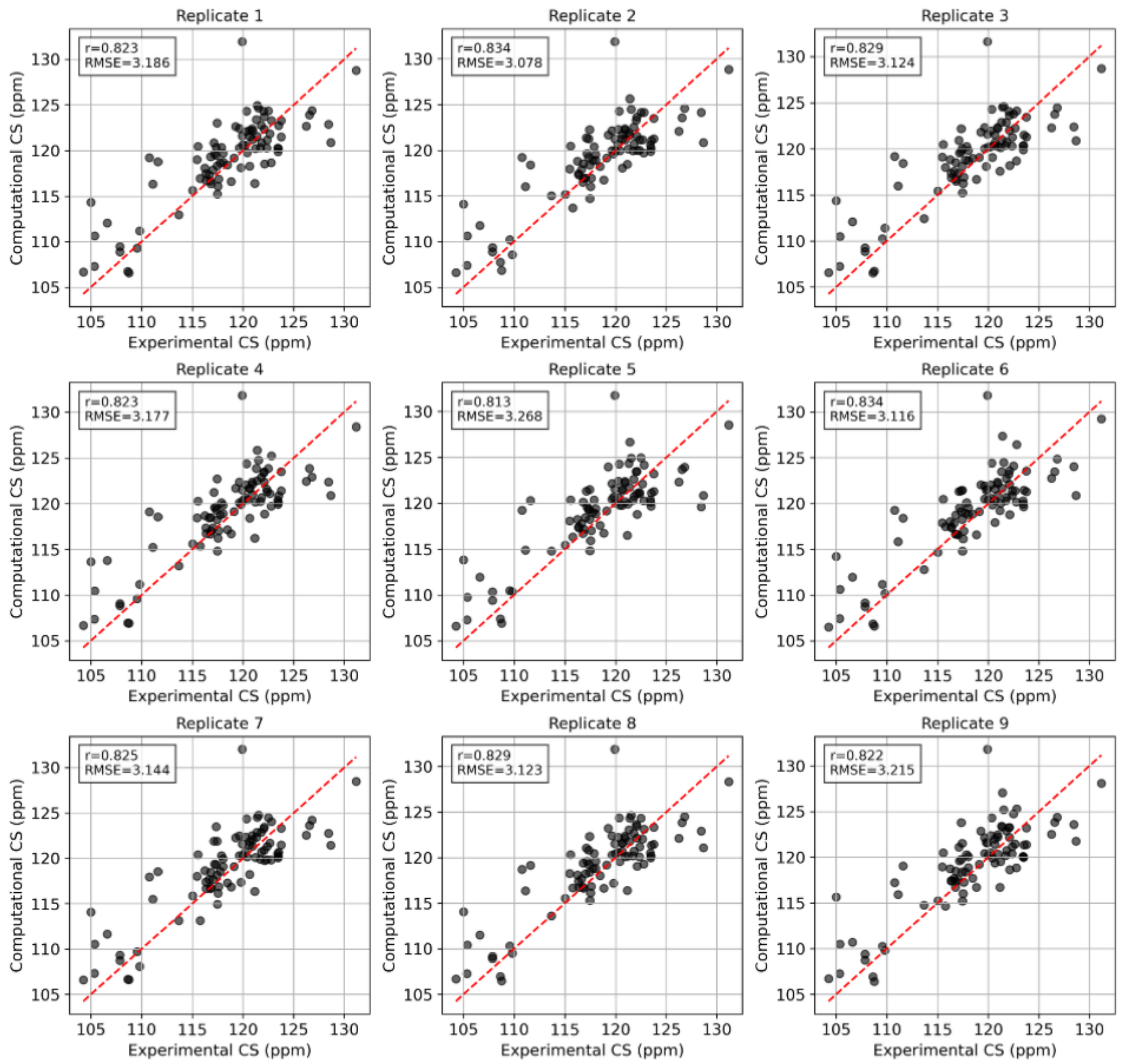

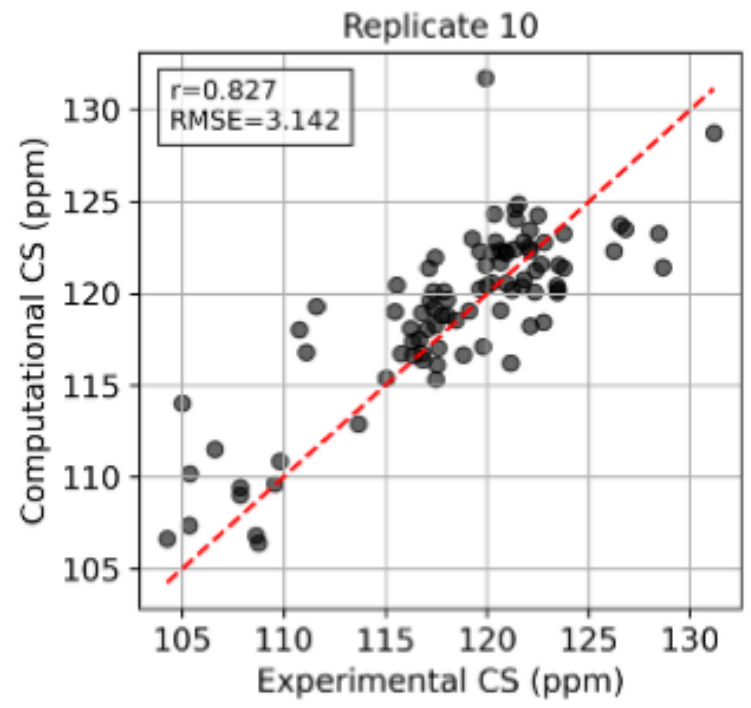

SPARTA+ - NH	Rep. 1	Rep. 2	Rep. 3	Rep. 4	Rep. 5	Rep. 6	Rep. 7	Rep. 8	Rep. 9	Rep. 10
Correlation coefficient	0.823	0.834	0.829	0.823	0.813	0.834	0.825	0.829	0.822	0.827
RMSE (ppm)	3.186	3.078	3.124	3.177	3.268	3.116	3.144	3.123	3.215	3.142

Supplementary Figure 10. Sparta+ Correlations for atom type NH (backbone) for the neurotensin-1 receptor (experiment ID 7 in Table 1).

Sparta+ – HN (backbone)

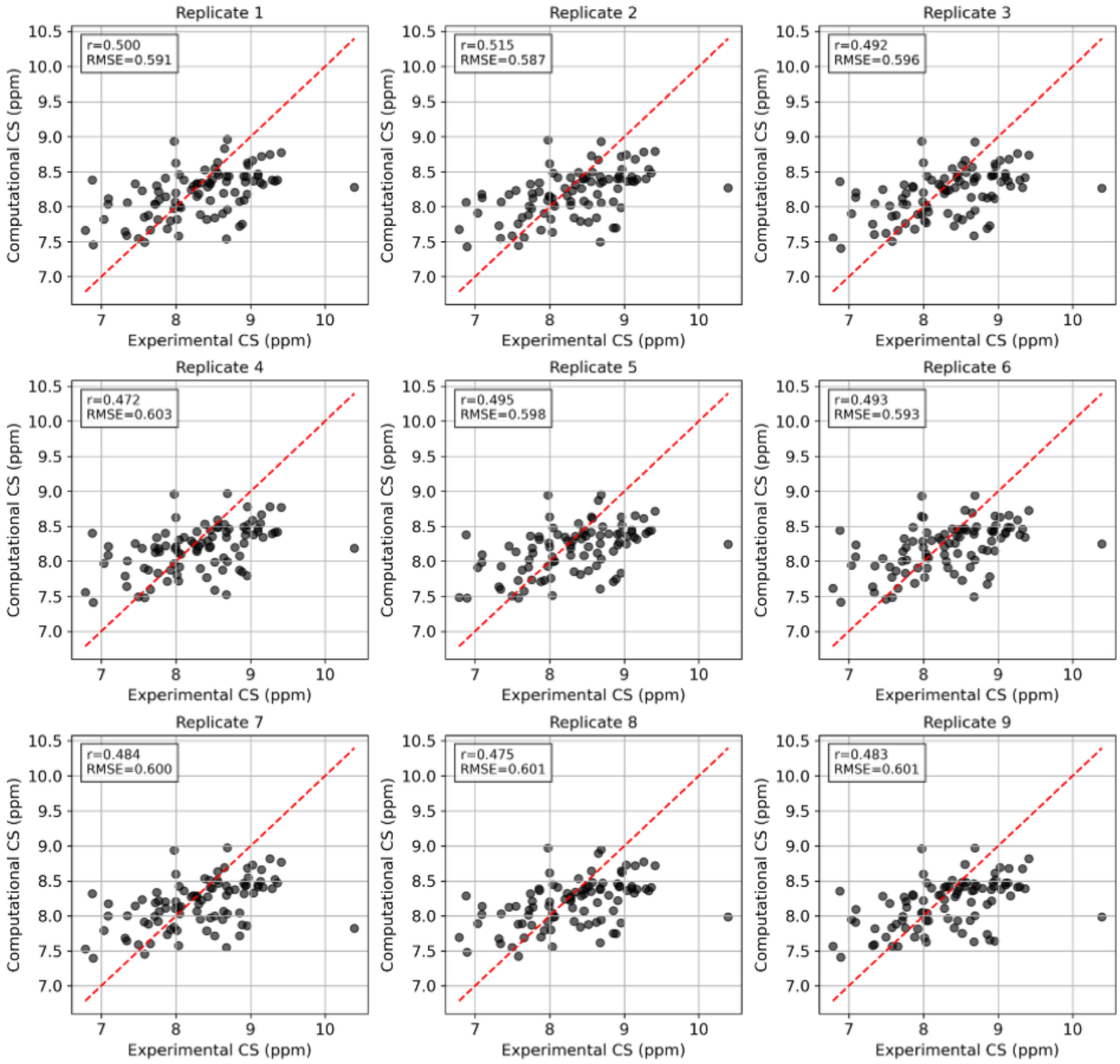

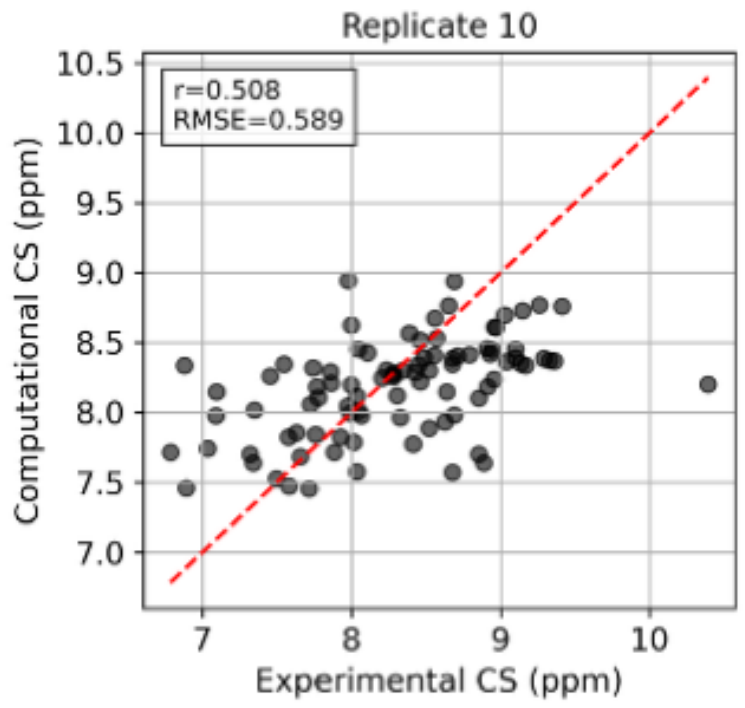

SPARTA+ - HN	Rep. 1	Rep. 2	Rep. 3	Rep. 4	Rep. 5	Rep. 6	Rep. 7	Rep. 8	Rep. 9	Rep. 10
Correlation coefficient	0.500	0.515	0.492	0.472	0.495	0.493	0.484	0.475	0.483	0.508
RMSE (ppm)	0.591	0.587	0.596	0.603	0.598	0.593	0.600	0.601	0.601	0.589

Supplementary Figure 11. Sparta+ Correlations for atom type HN (backbone) for the neurotensin-1 receptor (experiment ID 7 in Table 1).

UCBShift 2.0 – C’

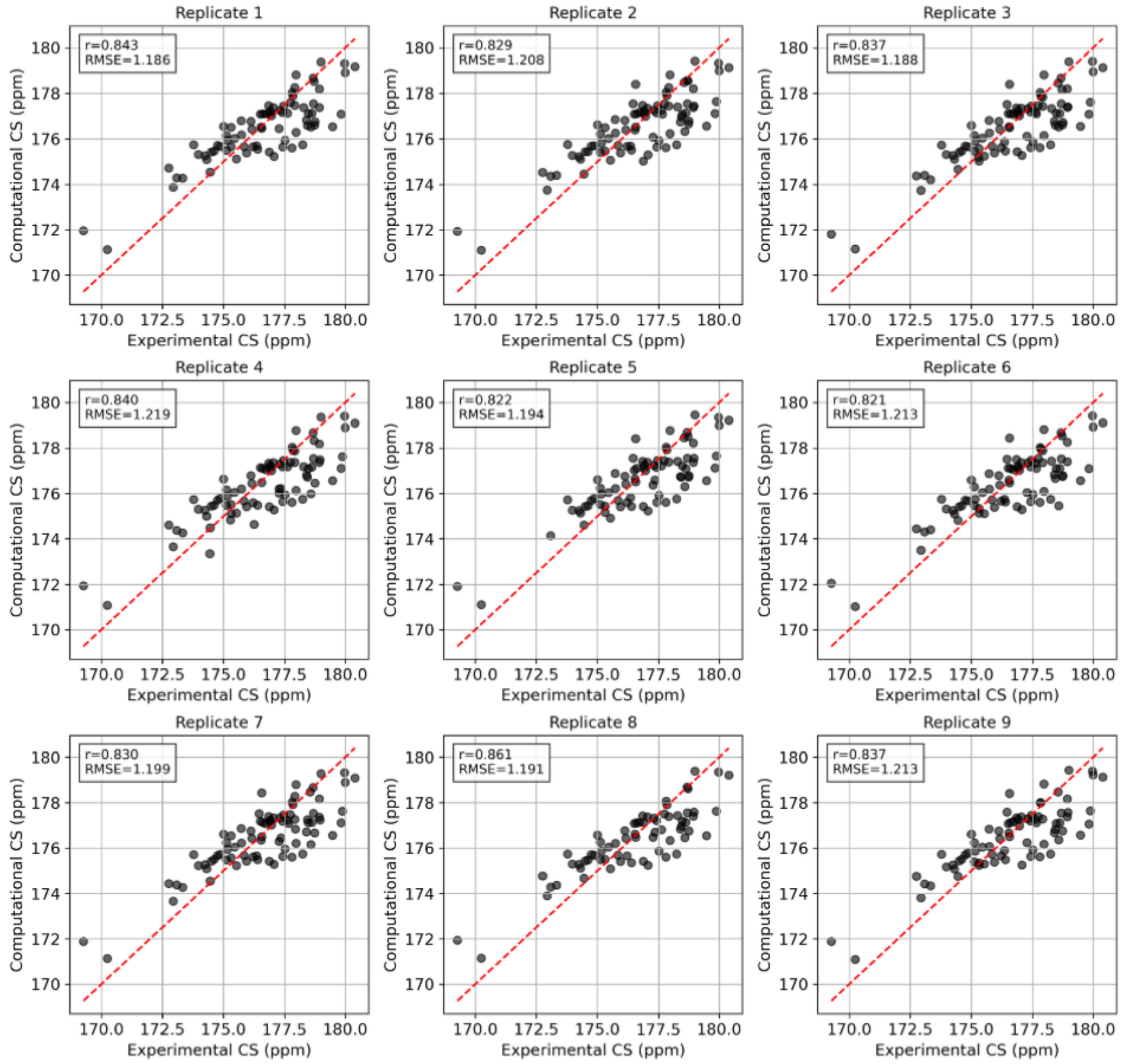

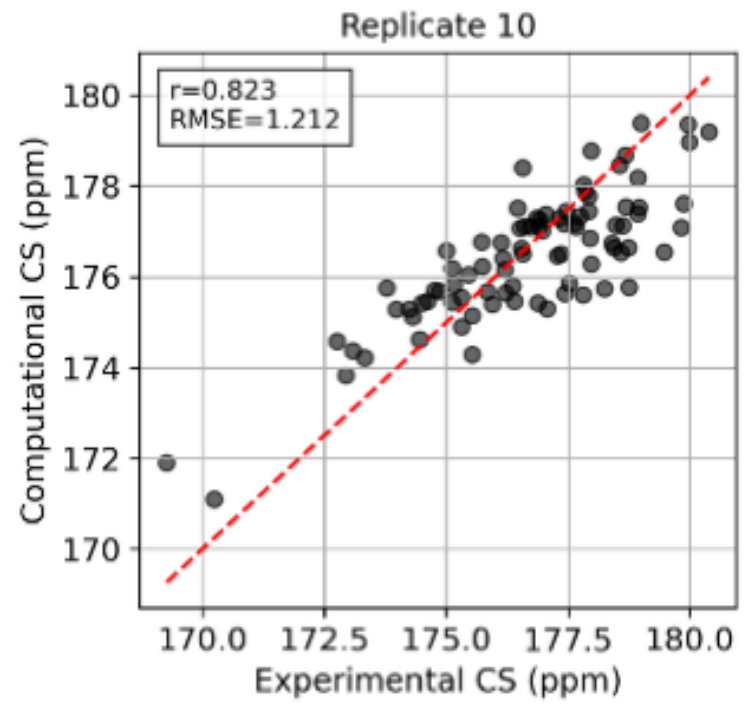

UCBShift 2.0 - C'	Rep. 1	Rep. 2	Rep. 3	Rep. 4	Rep. 5	Rep. 6	Rep. 7	Rep. 8	Rep. 9	Rep. 10
Correlation coefficient	0.843	0.829	0.837	0.840	0.822	0.821	0.830	0.861	0.837	0.823
RMSE (ppm)	1.186	1.208	1.188	1.219	1.194	1.213	1.199	1.191	1.213	1.212

Supplementary Figure 12. UCBShift 2.0 Correlations for atom type C’ (carboxyl) for the neurotensin-1 receptor (experiment ID 7 in Table 1).

UCBShift 2.0 – CA

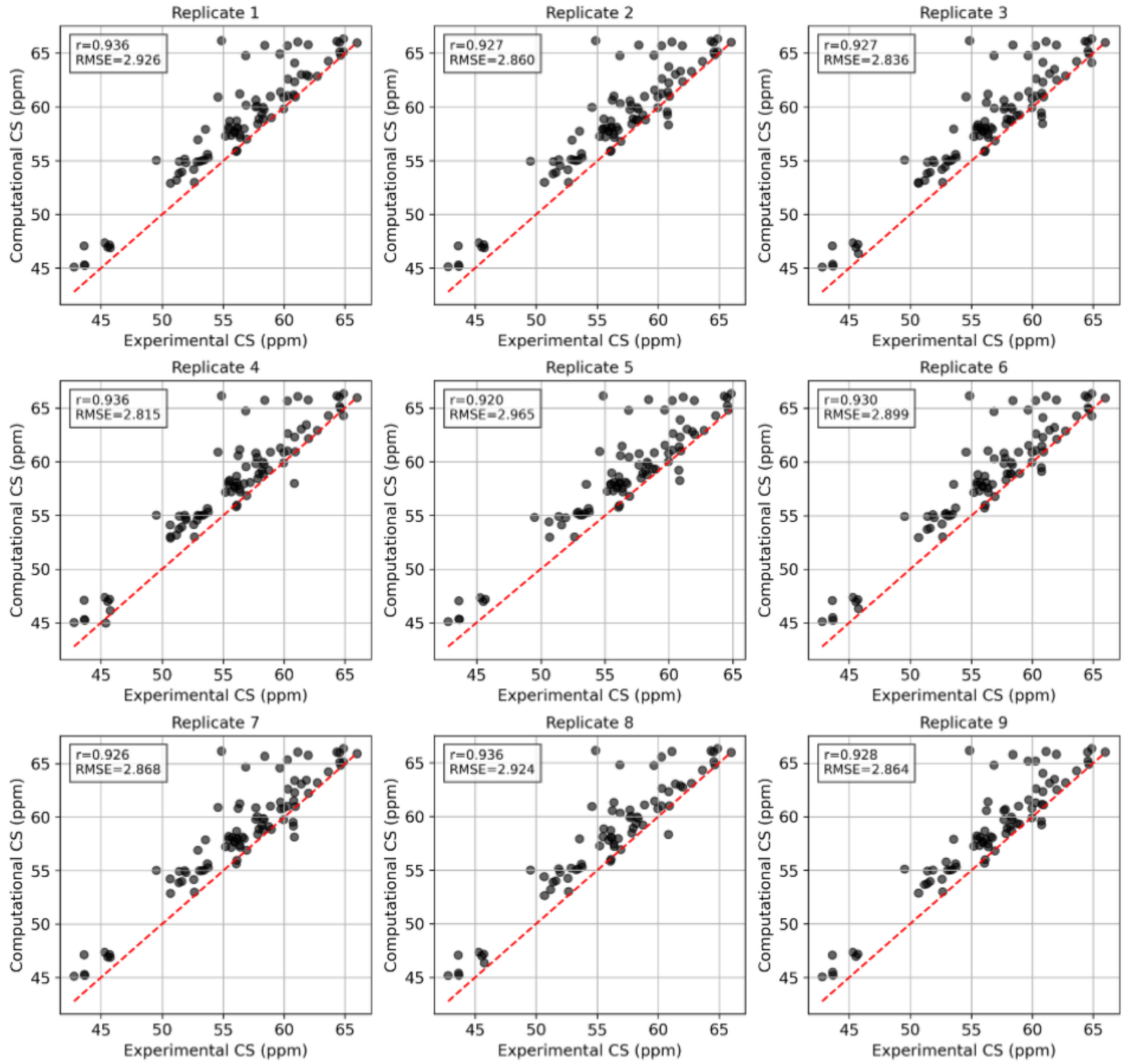

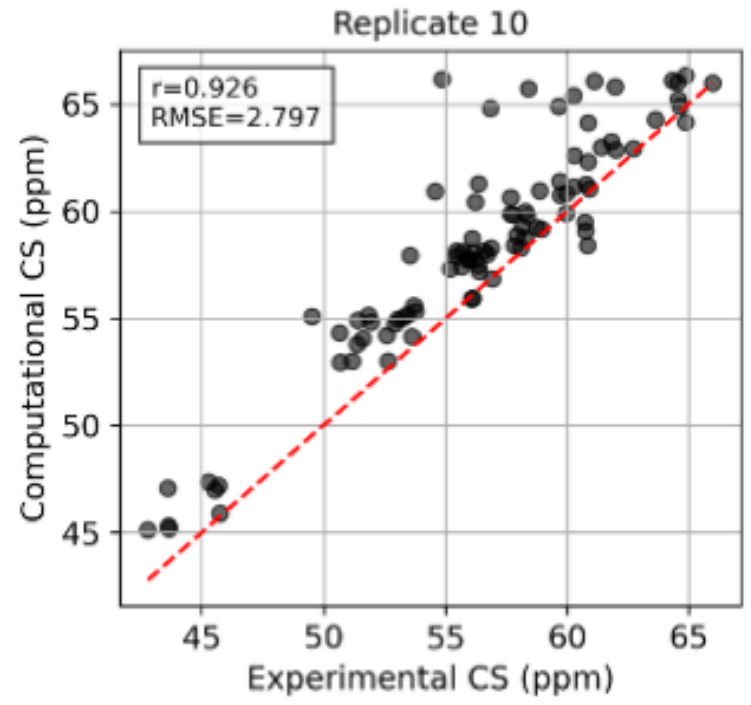

UCBShift 2.0 - CA	Rep. 1	Rep. 2	Rep. 3	Rep. 4	Rep. 5	Rep. 6	Rep. 7	Rep. 8	Rep. 9	Rep. 10
Correlation coefficient	0.936	0.927	0.927	0.936	0.920	0.930	0.926	0.936	0.928	0.926
RMSE (ppm)	2.926	2.860	2.836	2.815	2.965	2.899	2.868	2.924	2.864	2.797

Supplementary Figure 13. UCBShift 2.0 Correlations for atom type CA for the neurotensin-1 receptor (experiment ID 7 in Table 1).

UCBShift 2.0 – CB

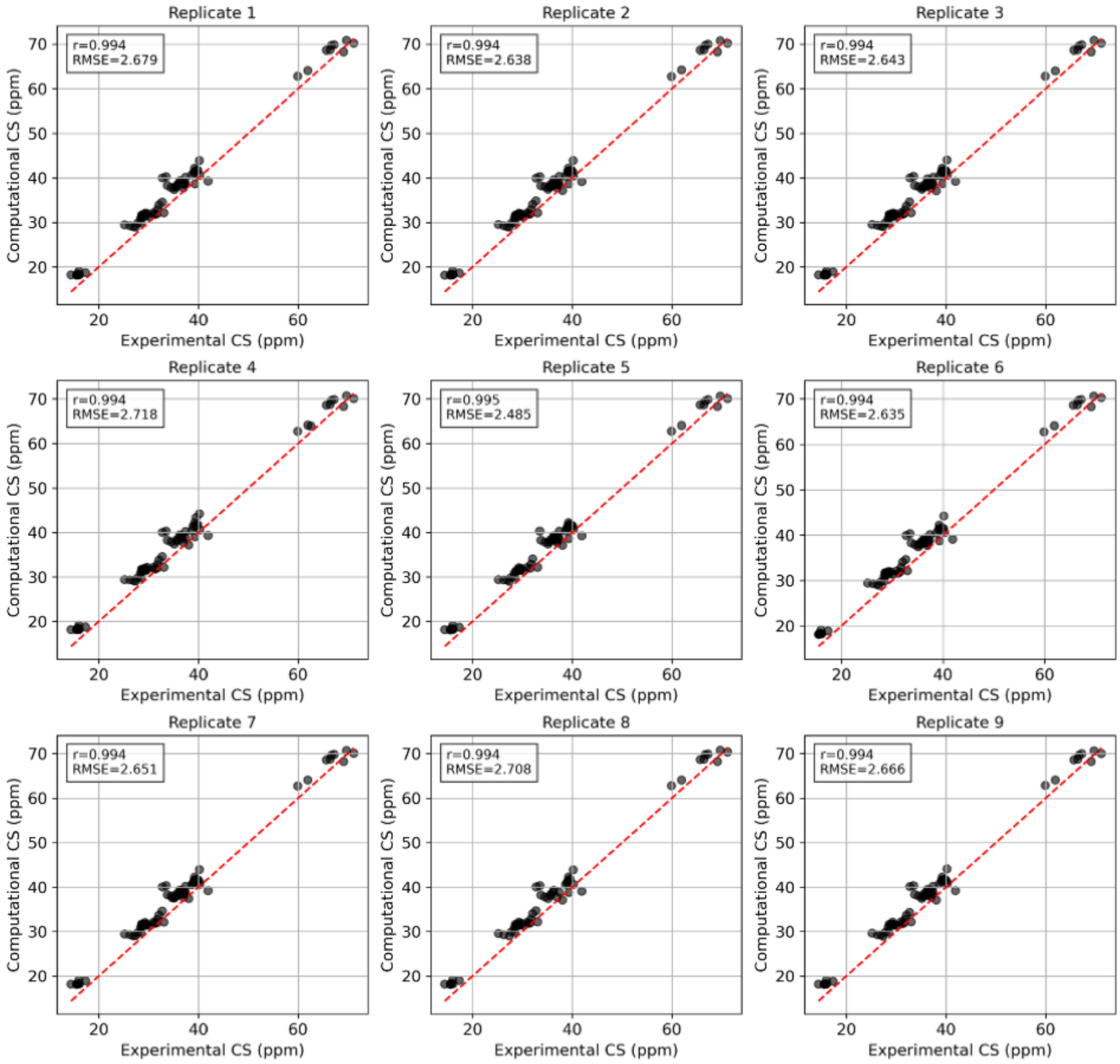

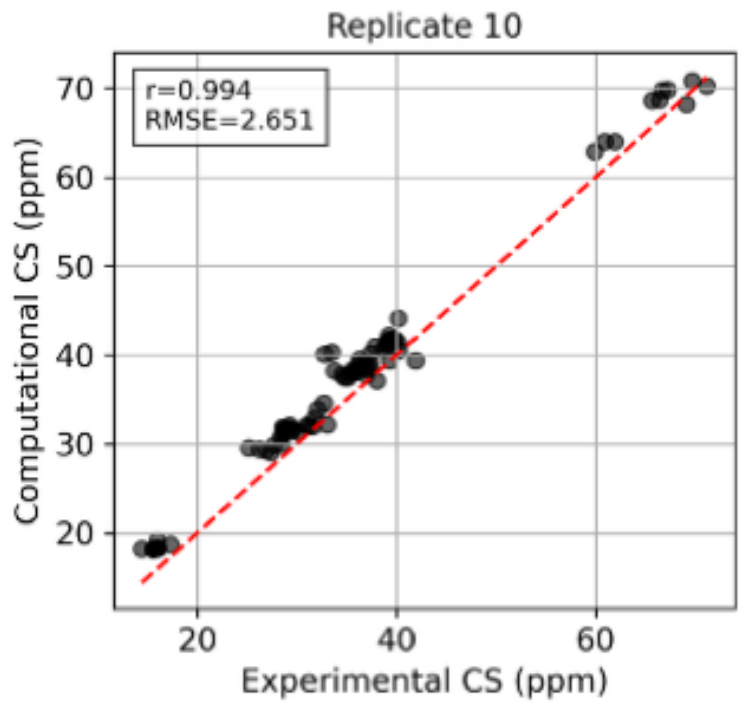

UCBShift 2.0 - CB	Rep. 1	Rep. 2	Rep. 3	Rep. 4	Rep. 5	Rep. 6	Rep. 7	Rep. 8	Rep. 9	Rep. 10
Correlation coefficient	0.994	0.994	0.994	0.994	0.995	0.994	0.994	0.994	0.994	0.994
RMSE (ppm)	2.679	2.638	2.643	2.718	2.485	2.635	2.651	2.708	2.666	2.651

Supplementary Figure 14. UCBShift 2.0 Correlations for atom type CB for the neurotensin-1 receptor (experiment ID 7 in Table 1).

UCBShift 2.0 – NH

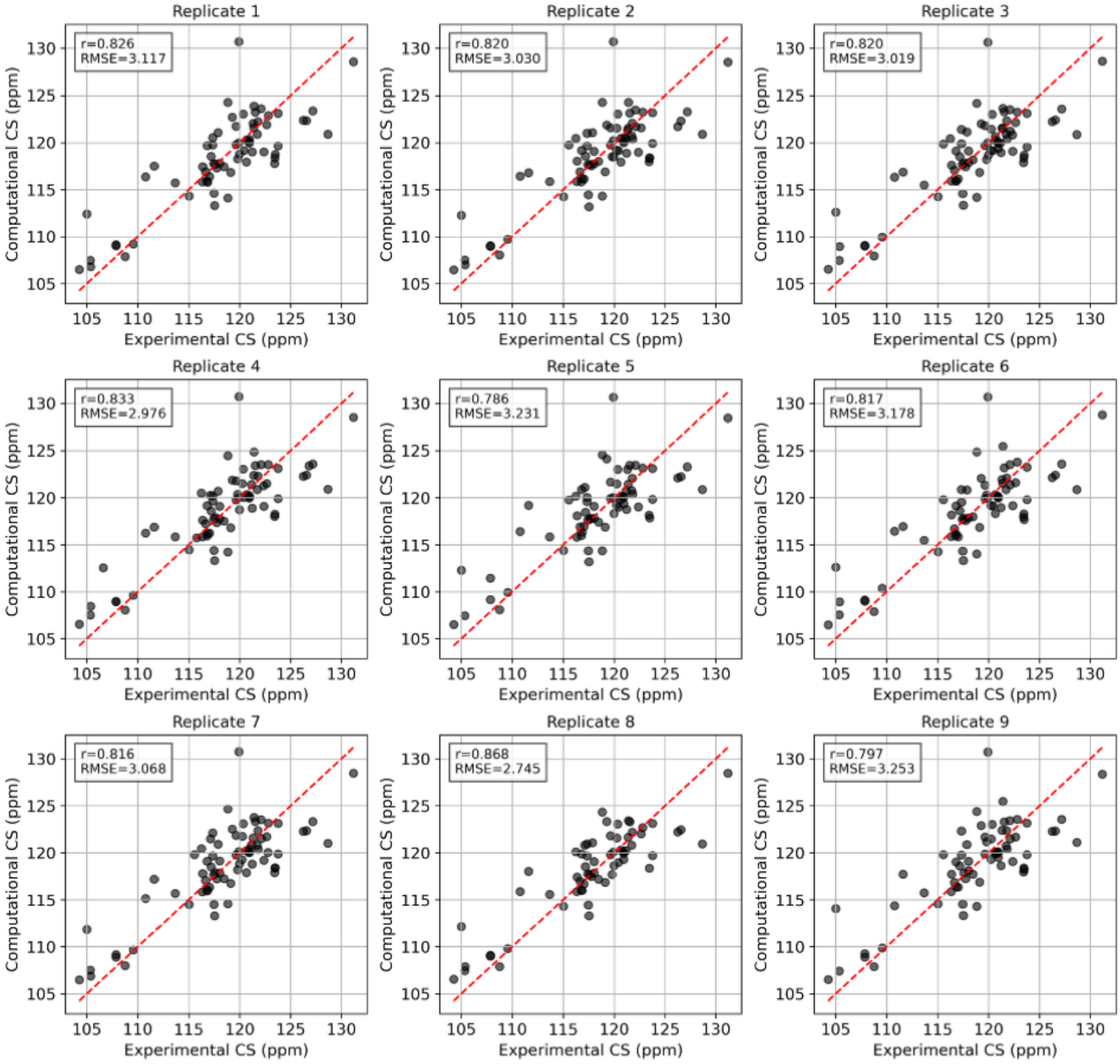

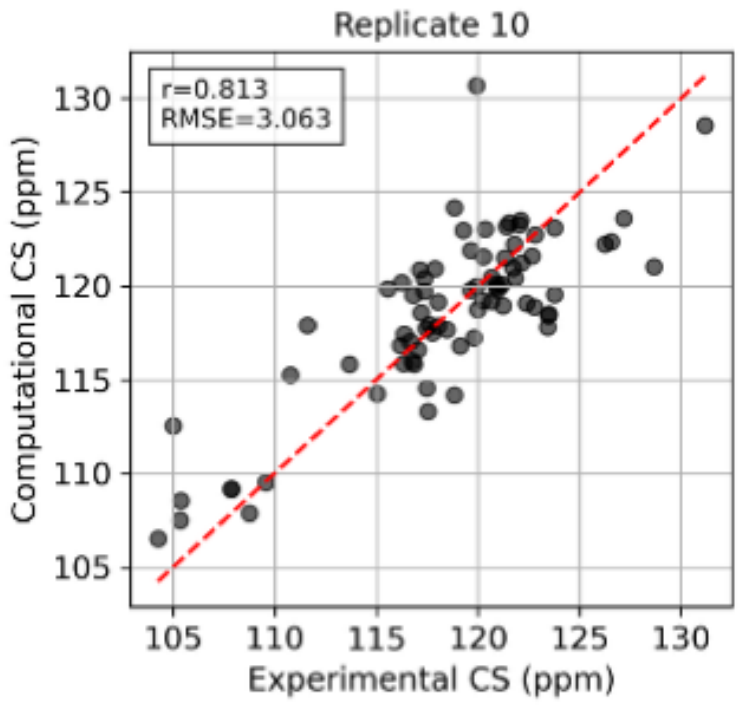

UCBShift 2.0 - NH	Rep. 1	Rep. 2	Rep. 3	Rep. 4	Rep. 5	Rep. 6	Rep. 7	Rep. 8	Rep. 9	Rep. 10
Correlation coefficient	0.826	0.820	0.820	0.833	0.786	0.817	0.816	0.868	0.797	0.813
RMSE (ppm)	3.117	3.030	3.019	2.976	3.231	3.178	3.068	2.745	3.253	3.063

Supplementary Figure 15. UCBShift 2.0 Correlations for atom type NH (backbone) for the neurotensin-1 receptor (experiment ID 7 in Table 1).

UCBShift 2.0 – HN (backbone)

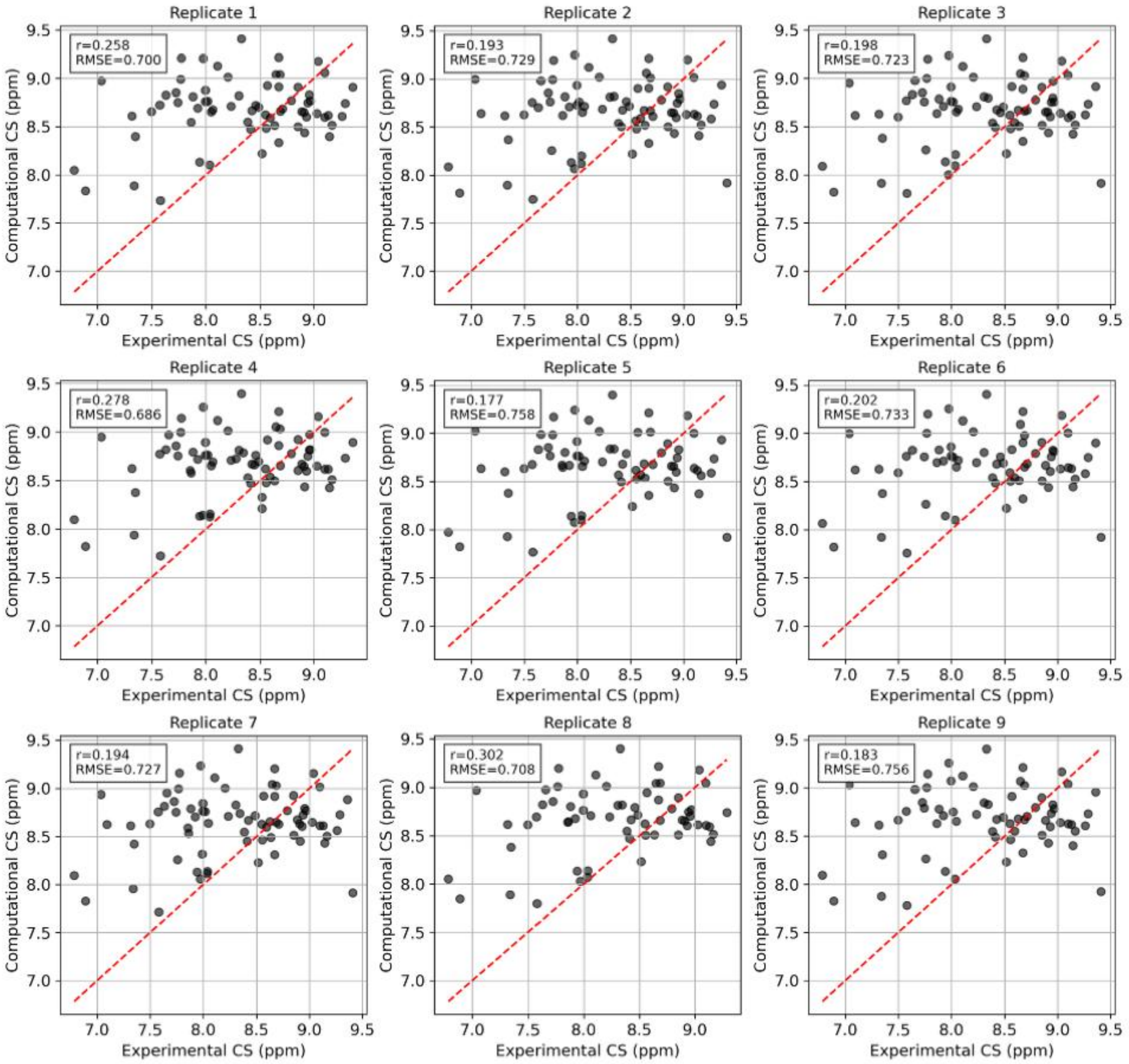

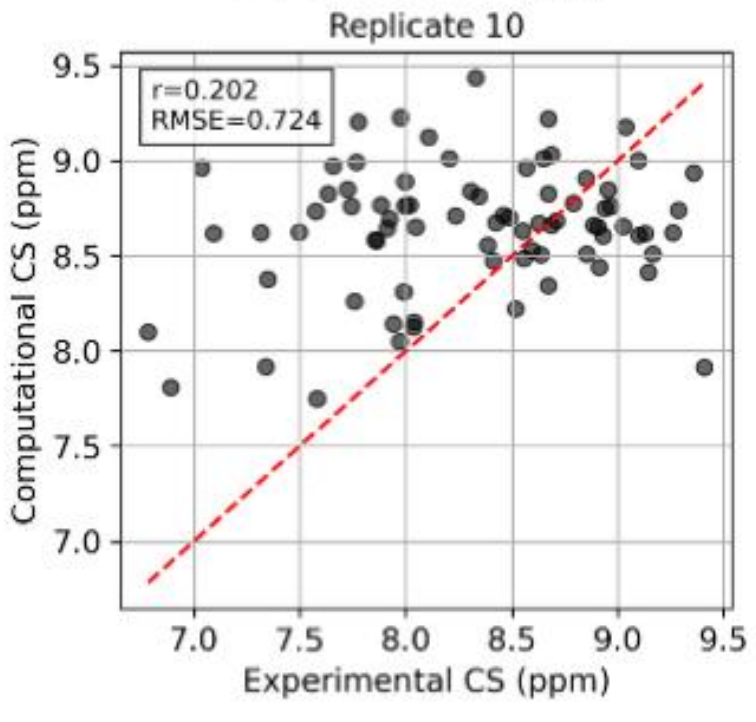

UCBShift 2.0 - HN	Rep. 1	Rep. 2	Rep. 3	Rep. 4	Rep. 5	Rep. 6	Rep. 7	Rep. 8	Rep. 9	Rep. 10
Correlation coefficient	0.258	0.193	0.198	0.278	0.177	0.202	0.194	0.302	0.183	0.202
RMSE (ppm)	0.700	0.729	0.723	0.686	0.758	0.733	0.727	0.708	0.756	0.724

Supplementary Figure 16. UCBShift 2.0 Correlations for atom type HN (backbone) for the neurotensin-1 receptor (experiment ID 7 in Table 1).

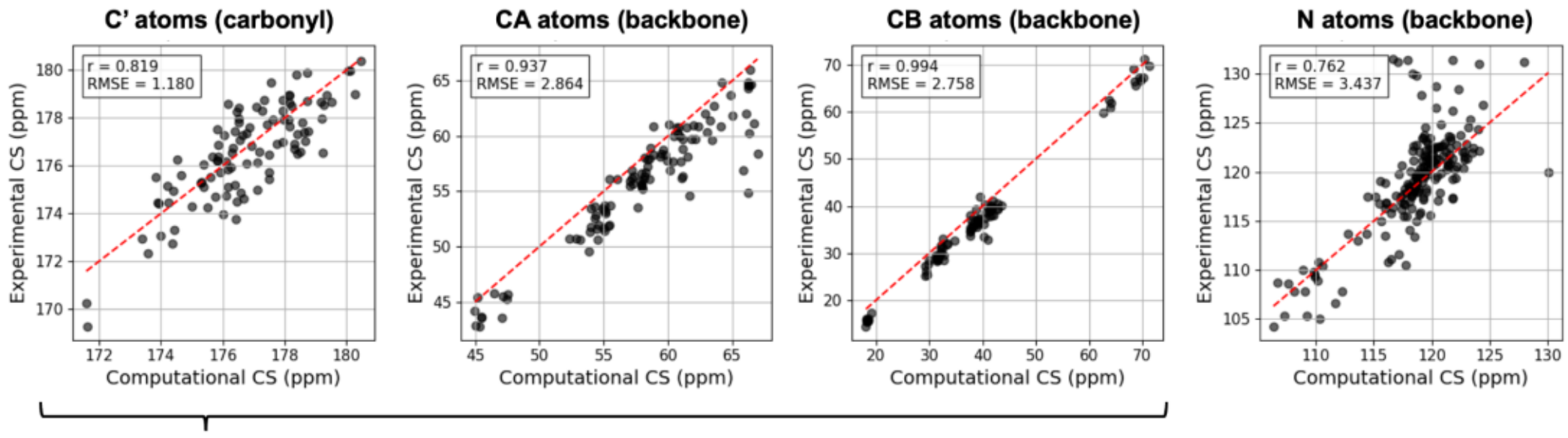

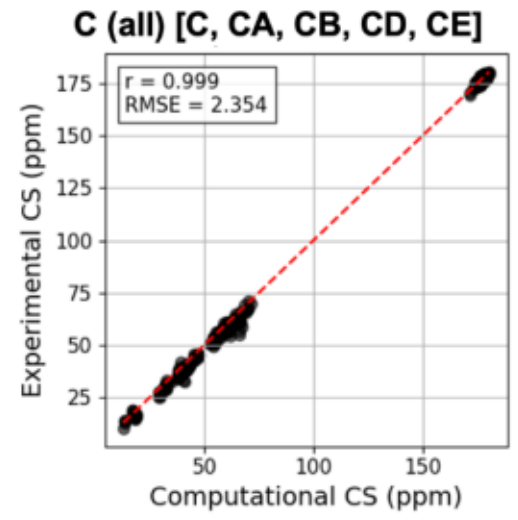

Atom type	Pearson r	RMSE	Exp. data points	Experiment IDs
C' (carbonyl)	0.819	1.180	101	12
CA	0.937	2.864	106	12
CB	0.994	2.758	86	12
N	0.762	3.437	188	9, 11, 12
H	0.321	0.780	188	9, 11, 12
C (all)	0.999	2.354	303	8, 9, 10, 11, 12

Supplementary Figure 17. Benchmarking of GPCRmd-NMR CS prediction against NMR experimental datasets. The table presents the benchmarking results of GPCRmd-NMR CS predictions against experimental data from diverse studies (IDs 8–12). All experimental datasets were combined into a single correlation matrix, and the performance of the CS predictor ShiftX2 was evaluated across different atom types. The reported number of experimental data points corresponds to the total CS signals obtained from studies with IDs 8–12. Detailed information about each receptor system included in these experiments (Experiment ID) is provided in Table 1.

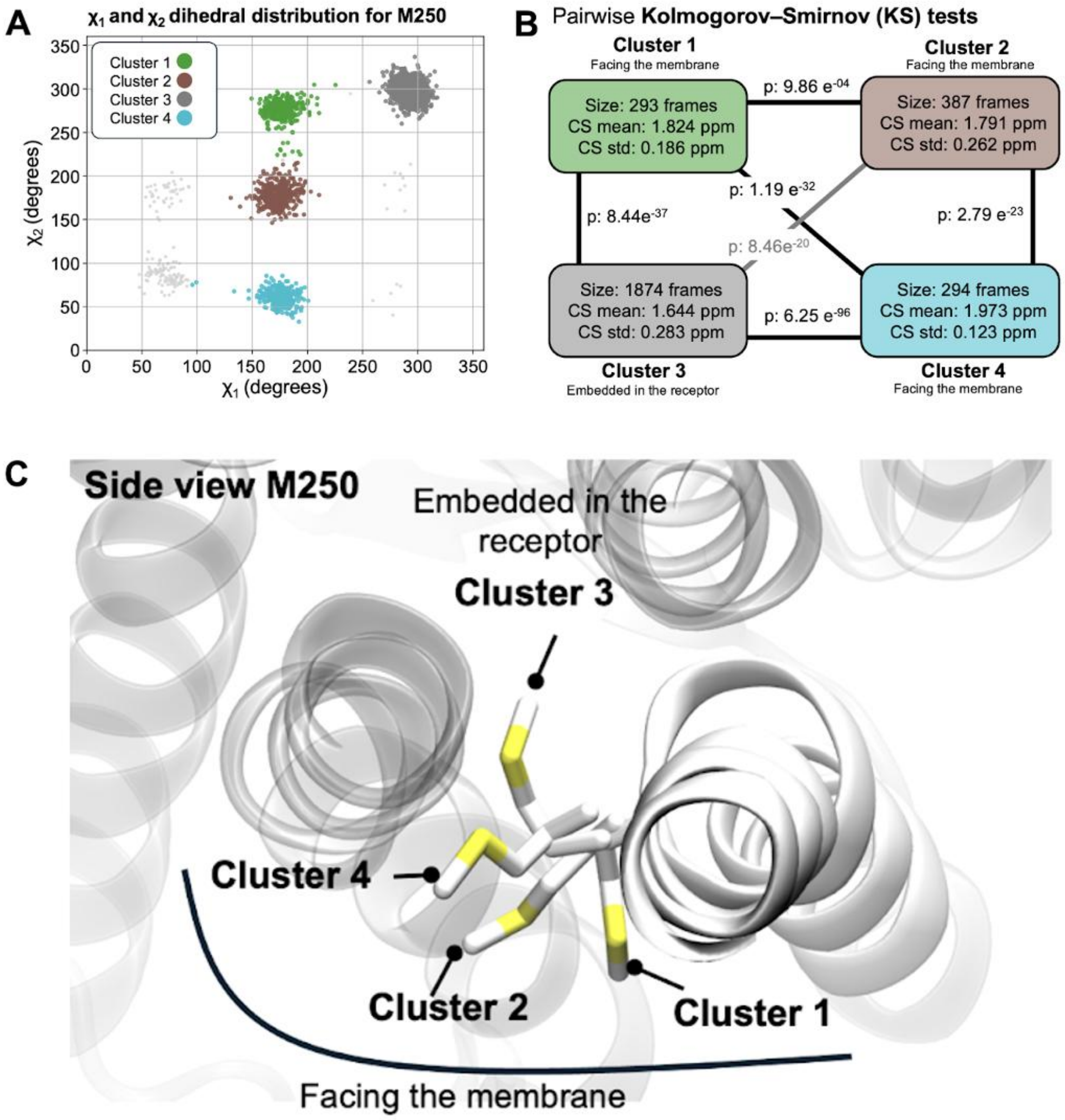

Supplementary Figure 18. Conformational landscape of M250 χ-dihedrals. (**A**) $\chi 1$–$\chi 2$ dihedral distribution for M250 across all three MD replicates. Each point represents one trajectory frame, colored by the four conformational clusters identified using an unsupervised density-based algorithm (OPTICS): Cluster 1 (green), Cluster 2 (brown), Cluster 3 (grey) and Cluster 4 (cyan); unassigned points are shown in light grey. **(B)** Pairwise Kolmogorov–Smirnov tests comparing CS distributions between clusters. For each cluster, the number of frames, mean CS, and standard deviation are provided. Lines indicate p-values, demonstrating significant differences in CS between clusters. **(C)** Structural representation of M250 cluster centroids. Clusters 1, 2, and 4 point toward the membrane, while Cluster 3 is embedded in the receptor. Interestingly, the receptor-embedded M250 exhibits two distinct CS populations (see Main Text, Figure 3), yet only a single conformational cluster (cluster 3) is observed. This indicates that the CS signals are not determined solely by the M250 χ dihedral angles, but also reflect changes in the local environment.

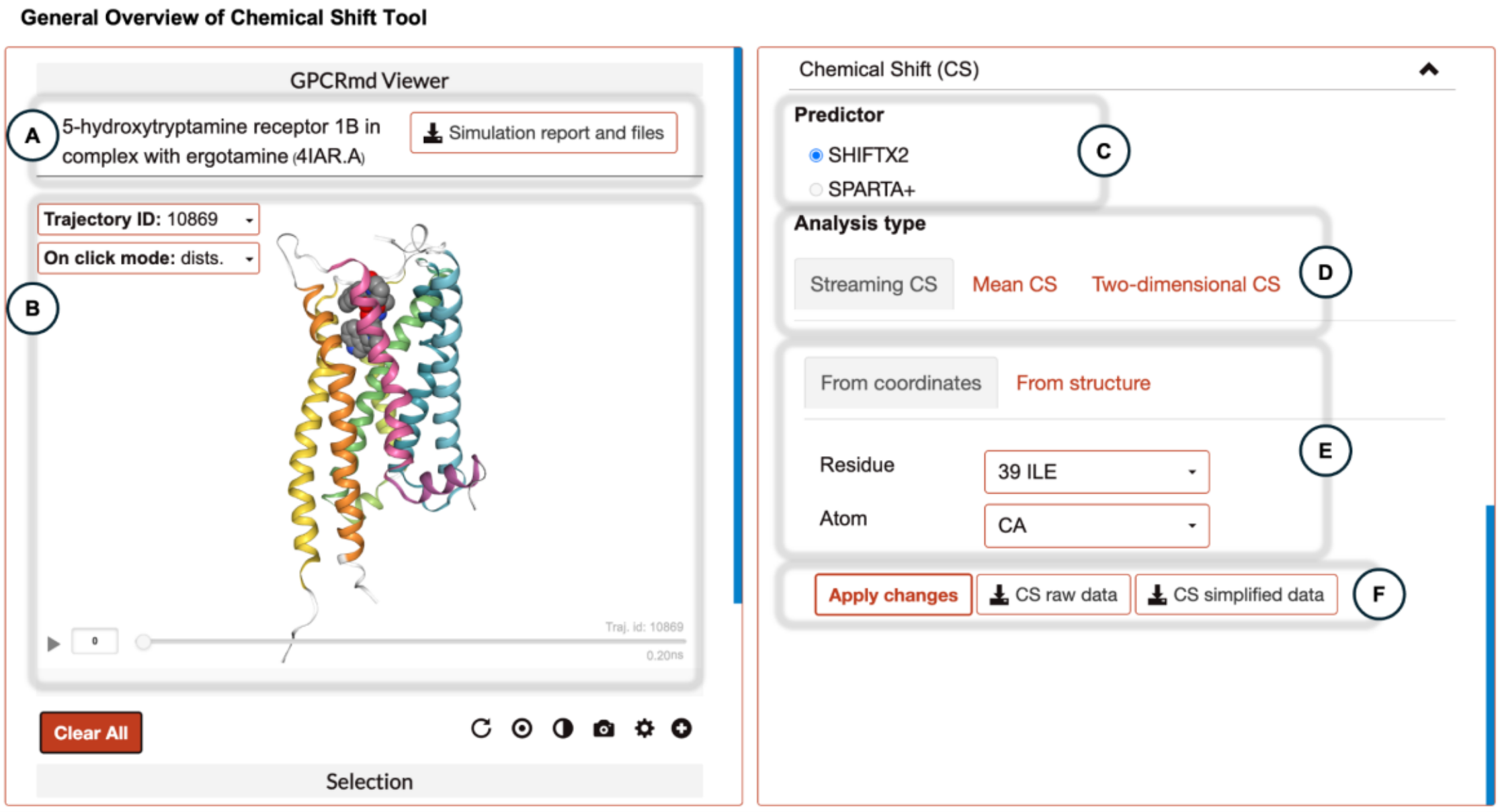

Supplementary Figure 19. General overview of GPCRmd chemical shift tool. (**A**) Name of the receptor and PDB code. The simulation report and files button redirects the user to an in-depth page where all the necessary files to reproduce the MD and the trajectories can be downloaded. (**B**) GPCRmd viewer, where the user can visualize the MD simulation. (**C**) Allows to select which predictor to use when showing the results. (**D**) Allows to select a visualization mode to display the CS values. (**E**) Allows to select the atoms to display the chemical shifts. (**F**) Apply changes button will generate the plot with the specified visualization mode and atoms. The user can also download the csv files containing the chemical shifts for each atom on the system.

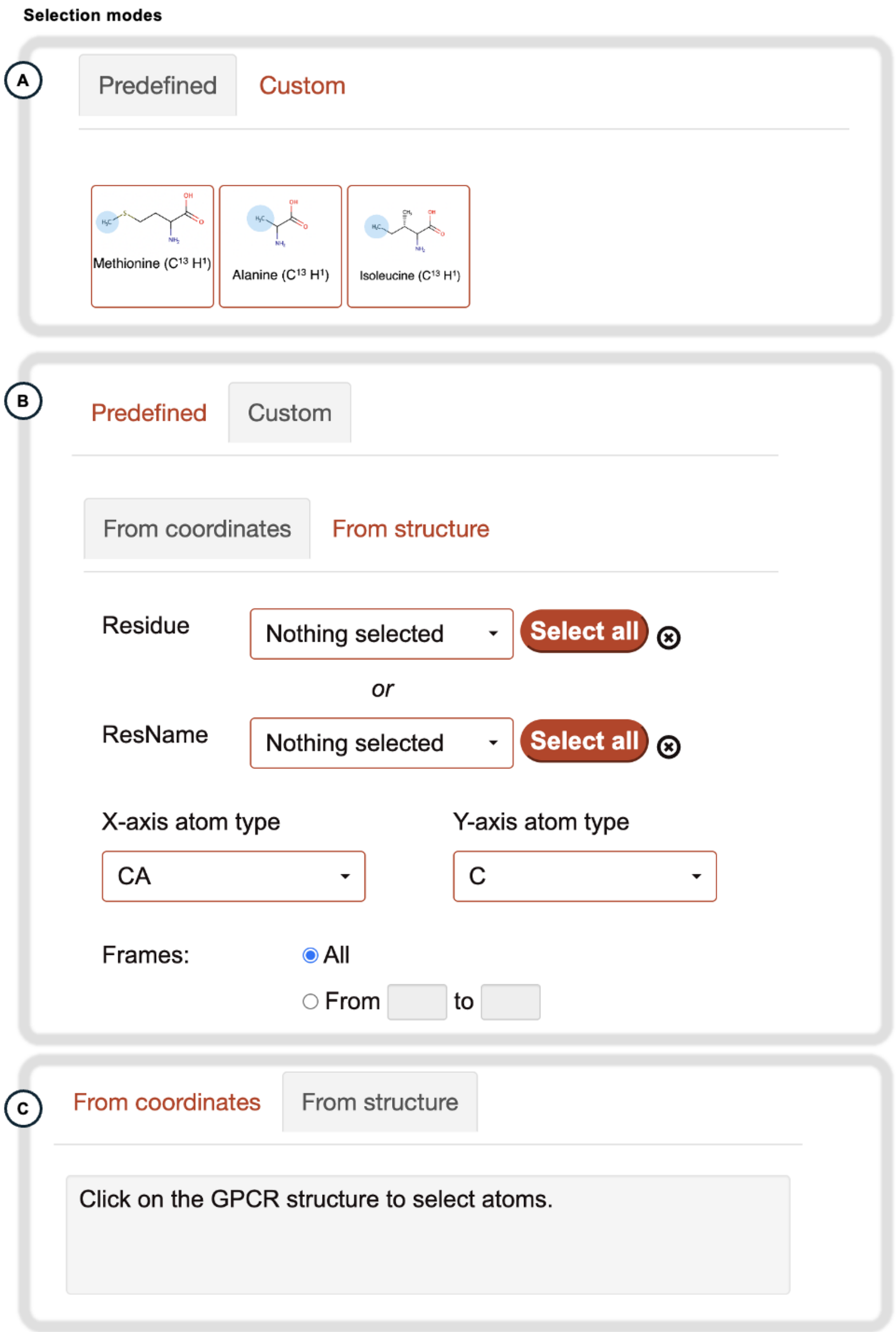

Supplementary Figure 20. Selection modes in GPCRmd Chemical Shift tool. (**A**) There are 3 predefined groups of atoms based on the most common labeled atoms in NMR experiments (Methionines: Cε and Hε; Alanines: Cβ and Hβ; Isoleucines: Cδ and Hδ). This selection mode is not available in the streaming chemical shift mode because only 1 atom should be selected. (**B**) The user can choose between selecting residues by their IDs or names, as well as which atoms types to include. This selection mode is specific for each visualization mode. (**C**) The user can also select atoms directly through the GPCRmd viewer

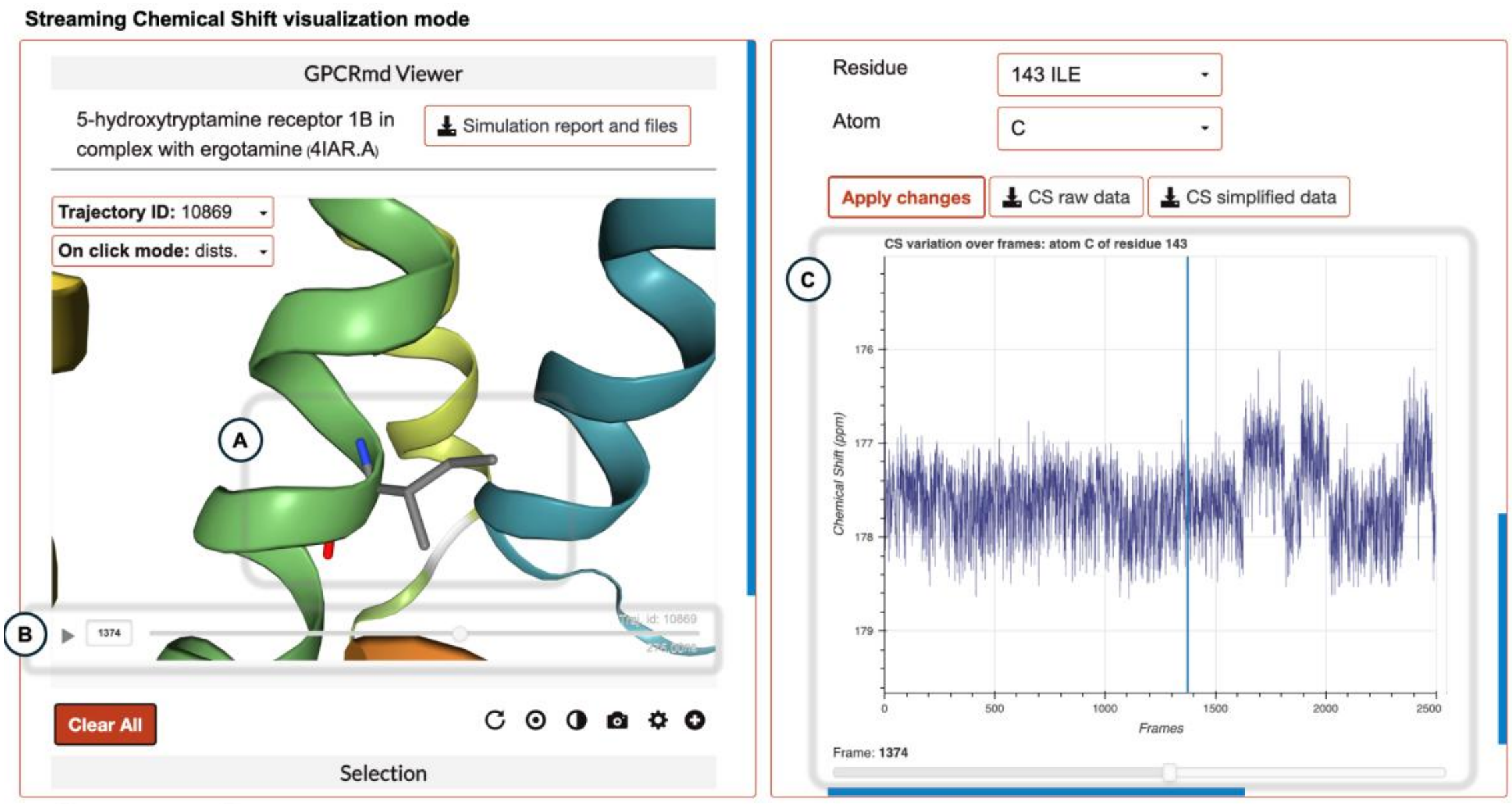

Supplementary Figure 21. Streaming Chemical Shift visualization mode. In this mode the user can interactively see the evolution of the chemical shifts over the molecular simulation. (**A**) The selected atom is zoomed in as the CS values are displayed. (**B**) The user can play the molecular simulation using the GPCRmd viewer. (**C**) Evolution of the chemical shift for the selected residue. A blue line and a progress button keeps track of the current frame displayed on the GPCRmd viewer. An accumulative histogram of the chemical shift values appears on the right part of the plot if the user hovers through it.

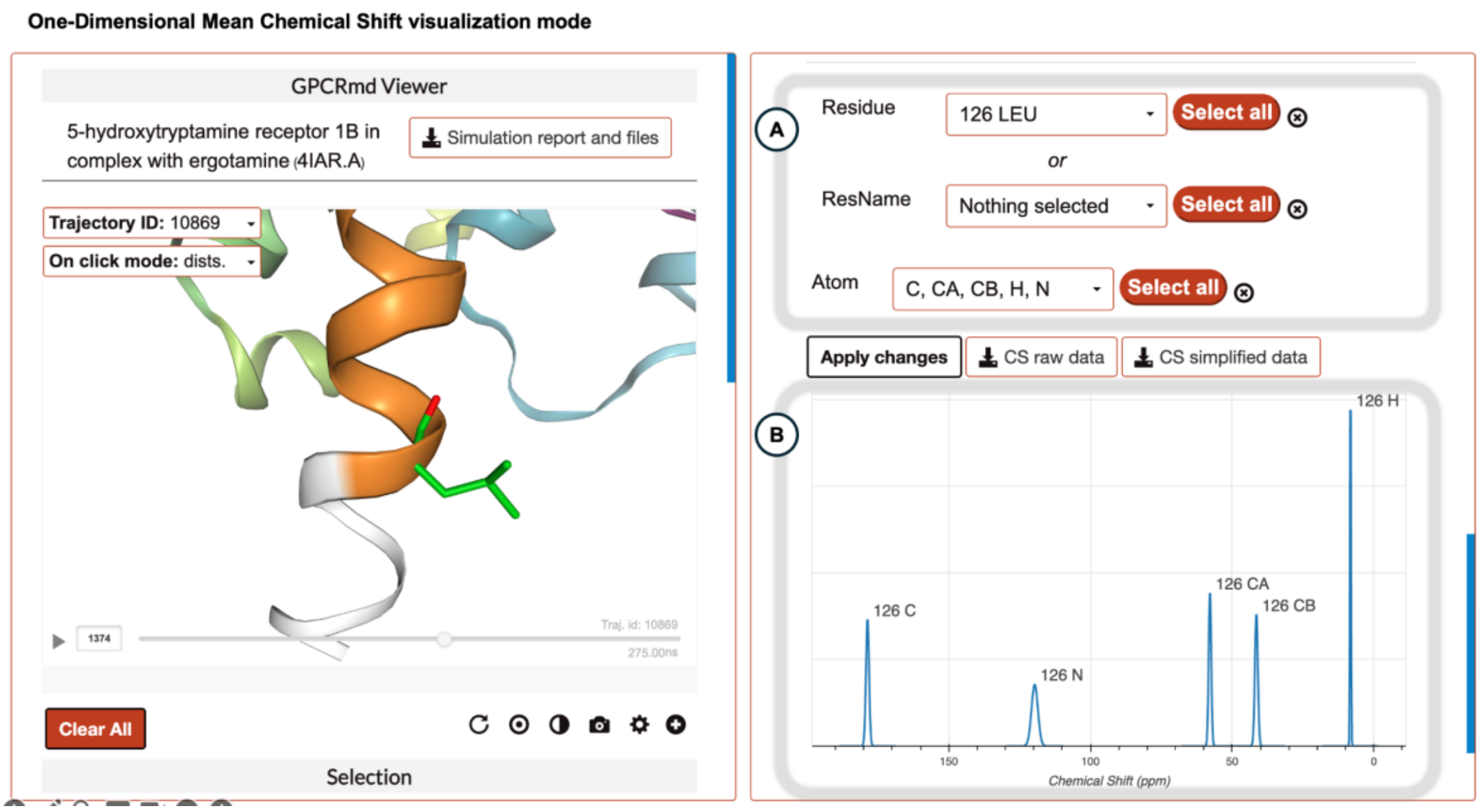

Supplementary Figure 22. One-Dimensional Mean Chemical Shift visualization mode. This mode allows the user to quickly compare chemical shift values over multiple residues and atom types. (**A**) The selection mode allows the user to select multiple atom types and residues at the same time. (**B**)

The mean signals for each selected atom are displayed. The width of the signal represents the associated error to the predicted value.

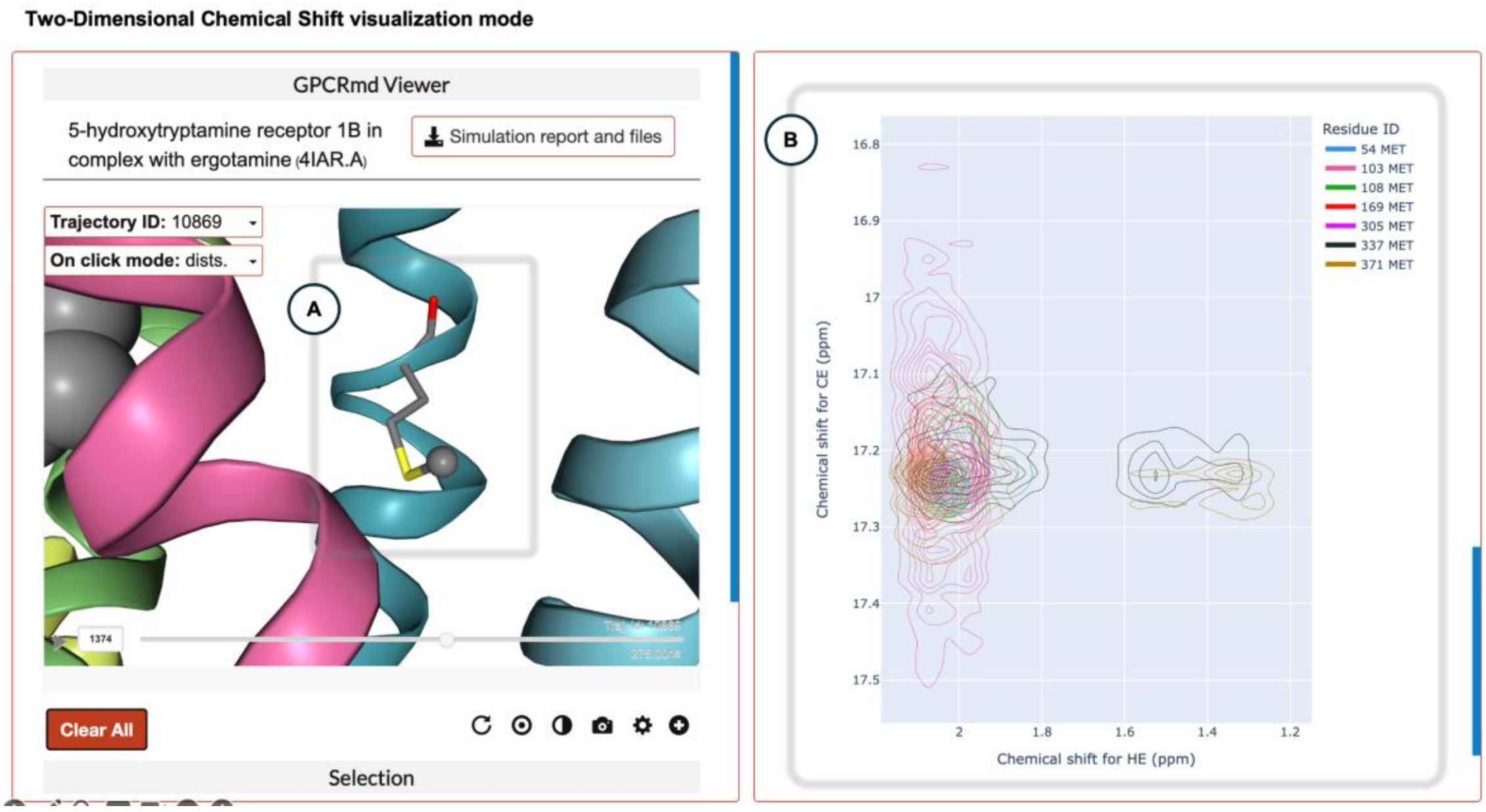

Supplementary Figure 23. Two-Dimensional Chemical Shift visualization mode. This mode allows the user to compare different signals for 2 atoms over the same residues. It becomes especially useful when trying to detect which residues are the most different between the selected ones. (**A**) GPCRmd viewers zooms in on the selected residue in the right plot. (**B**) The user can interactively select the residues using the legend to get an isolated representation of their signal in the plot and it will be zoomed in the GPCRmd viewer.

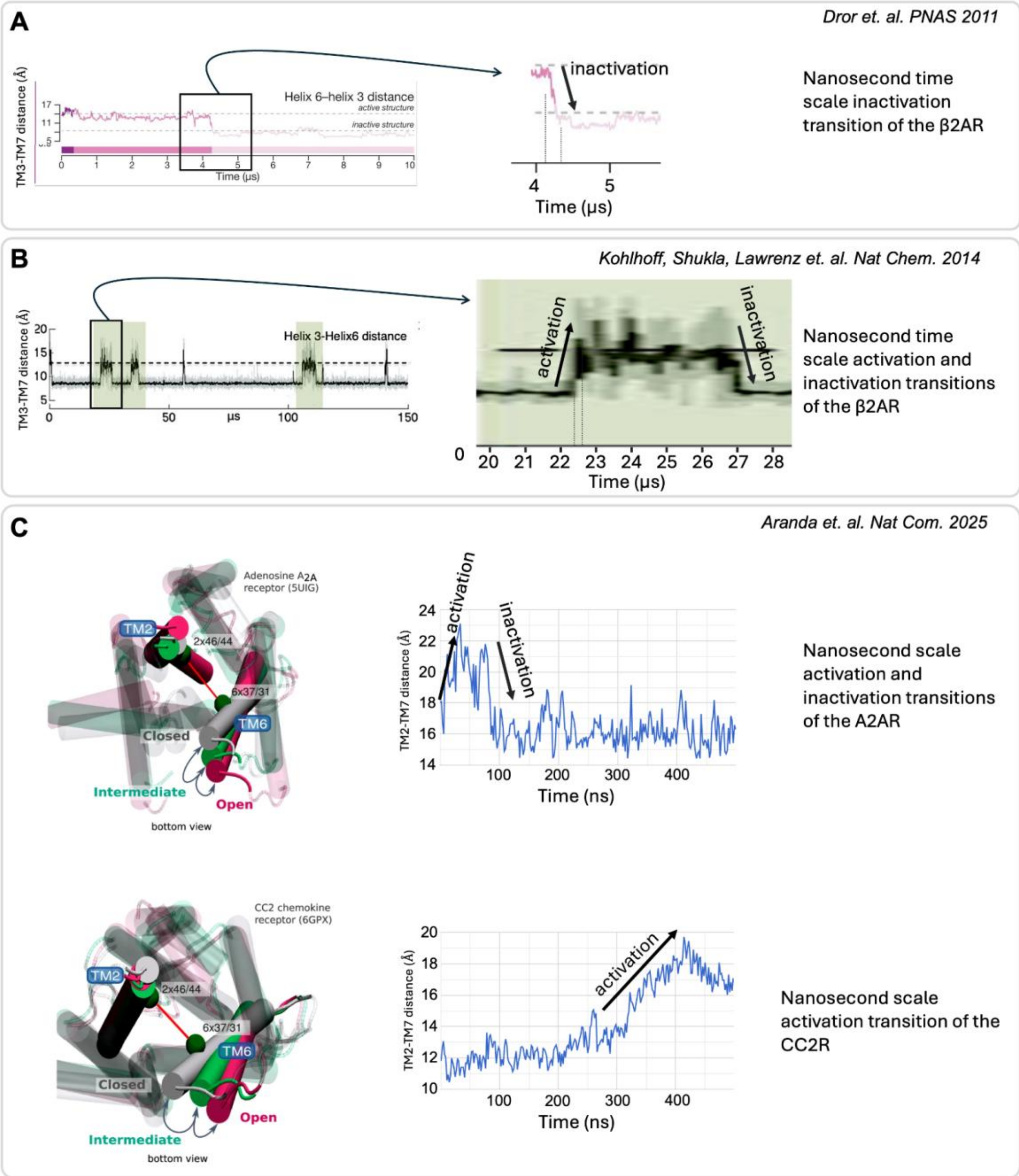

Supplementary Figure 24. **Sampling GPCR transitions at the nanosecond timescale through classical unbiased MD. (A)** Dror et al. sampled the inactivation of the β_2AR, observing that the TM6–TM3 distance rapidly decreases over just a few nanoseconds once the transition begins. **(B)** Kohlhoff, Shukla, Lawrenz et al. captured both activation and inactivation processes of the β_2AR, likewise monitoring the TM6–TM3 distance detecting fast transitions once initiated. **(C)** Aranda et al. performed multiple 500-ns simulations across different GPCRs. In some cases they observed activation or inactivation events, each occurring on the nanosecond timescale once the process had begun.

#Text file containing the mean chemical shift value for each atom predicted
#Int_Error = Intrinsic Error associated with the CS prediction made by SHIFTX+
#Ext_Error = Extrinsic error associated to the fact that the MD is not infinite. The error is estimated by performing a block analysis for each atom.
#Total_Error = Sum of squares of the Int_Error and the Ext_Error
ResidueID ResidueNAME Atom_Type Mean_CS(ppm) Int_Error(ppm) Ext_Error(ppm) Total_Error(ppm)
2 SER C 175.259 0.533 0.0119 0.5331
2 SER CA 57.6037 0.4412 0.0062 0.4412
2 SER CB 64.2798 0.5163 0.0236 0.5168
2 SER H 8.3008 0.1711 0.0021 0.1711
2 SER HA 4.5108 0.1231 0.0011 0.1231
2 SER HB2 4.0537 0.9482 0.004 0.9482
2 SER HB3 3.9736 0.9482 0.0016 0.9482
2 SER N 117.3538 1.1169 0.0088 1.1169
3 SER C 176.0649 0.533 0.0103 0.5331
3 SER CA 60.7321 0.4412 0.0154 0.4415
3 SER CB 62.9466 0.5163 0.0252 0.5169
3 SER H 8.784 0.1711 0.0017 0.1711
3 SER HA 4.2417 0.1231 0.0016 0.1231
3 SER HB2 3.9901 0.9482 0.0006 0.9482
3 SER HB3 3.9214 0.9482 0.0008 0.9482
3 SER N 120.7846 1.1169 0.2325 1.1408
4 VAL C 177.9411 0.533 0.0054 0.533
4 VAL CA 65.6378 0.4412 0.0136 0.4414
4 VAL CB 31.6399 0.5163 0.0031 0.5163
4 VAL CG1 21.1209 0.9787 0.0072 0.9787
4 VAL CG2 21.6315 0.9787 0.0123 0.9788
4 VAL H 8.0454 0.1711 0.0027 0.1711
4 VAL HA 3.8341 0.1231 0.0018 0.1231
4 VAL HB 2.1164 0.9482 0.0007 0.9482
4 VAL HG1 0.9679 0.9482 0.0007 0.9482
4 VAL HG2 0.9825 0.9482 0.001 0.9482
4 VAL N 120.545 1.1169 0.118 1.1231

Supplementary Figure 25. Simplified downloadable text file. The file provides for each atom, the Residue ID, Residue Name, Atom ID, mean chemical shift across the full trajectory (ppm), intrinsic error (ppm), extrinsic error (ppm), and the total predicted error (ppm).

resSeq	name	resname	resname_s	0	1	2	3	4	5	6	7	8	9
2	C	SER	S	175.0411	175.0542	175.0549	175.1978	175.1854	174.7136	175.1358	175.0777	174.9647	174.6995
2	CA	SER	S	57.5749	57.5916	57.5714	57.5264	57.4668	57.3127	57.6287	57.5367	57.5003	57.6733
2	CB	SER	S	64.9977	64.9978	64.6122	64.877	65.0282	64.4744	65.1524	65.0243	65.05	65.0656
2	H	SER	S	8.2158	8.2054	8.2329	8.1149	8.2373	8.2087	8.2624	8.2713	8.2247	8.1972
2	HA	SER	S	4.5943	4.5955	4.5116	4.5758	4.6364	4.5361	4.503	4.5706	4.5797	4.5325
2	HB2	SER	S	4.2593	4.2551	4.0231	4.2983	4.2618	4.2122	4.2652	4.2896	4.2722	4.2779
2	HB3	SER	S	4.1271	4.1271	4.0819	4.1409	4.1585	4.0615	4.1209	4.1089	4.0837	4.0598
2	N	SER	S	116.9524	116.9493	116.8158	117.3812	117.021	117.3237	117.2528	117.0931	117.1485	116.88
3	C	SER	S	175.7414	175.7003	176.1638	176.3789	176.2695	176.1611	176.2736	176.4685	176.155	176.3295
3	CA	SER	S	60.7848	60.7679	60.8524	60.683	61.2204	60.8606	60.7211	60.7936	61.1893	60.5975
3	CB	SER	S	62.8502	62.8212	63.1132	62.6418	63.0277	62.8349	62.8049	62.5354	62.9797	62.5963
3	H	SER	S	8.6937	8.6945	8.8402	8.8905	8.911	8.8133	8.8017	8.8364	8.8563	8.93
3	HA	SER	S	4.2725	4.272	4.2149	4.1871	4.2606	4.1484	4.2631	4.2527	4.2248	4.2156
3	HB2	SER	S	3.9611	3.959	4.0042	4.0558	3.9596	4.0289	4.0045	3.9945	3.9947	4.0446
3	HB3	SER	S	3.8983	3.8992	3.9387	3.9114	3.9182	3.9213	3.8908	3.9189	3.9563	3.9379
3	N	SER	S	117.6321	117.6498	117.9627	117.6812	117.6362	121.4099	117.9471	117.9323	118.0843	117.1234
4	C	VAL	V	177.9144	177.8804	178.3455	178.7217	178.6495	178.4837	177.9682	178.2868	178.4621	177.915
4	CA	VAL	V	65.7266	65.6868	65.8222	65.2758	65.8691	65.7717	66.3688	66.1702	66.0952	66.4364
4	CB	VAL	V	31.4835	31.4835	31.4735	31.4232	31.4956	31.6645	31.5434	31.4554	31.4958	31.6316
4	CG1	VAL	V	20.8627	20.8599	21.4021	21.2284	21.3761	21.4377	21.1304	21.438	21.4666	21.6712
4	CG2	VAL	V	21.8792	21.8451	21.8172	21.8391	21.8953	21.326	22.2662	21.6703	21.7038	22.0056
4	H	VAL	V	7.968	7.9728	8.2525	8.3052	8.2515	8.1372	8.3067	8.2864	8.2306	8.1535
4	HA	VAL	V	3.9627	3.9672	3.8805	3.8665	3.9341	3.9142	3.7975	3.9192	3.8033	3.7373
4	HB	VAL	V	2.1568	2.1649	2.1298	2.1423	2.1609	2.1317	2.1405	2.1686	2.1619	2.1833
4	HG1	VAL	V	1.004	0.985	0.9939	0.9718	1.0165	0.9799	0.9526	1.0142	0.9474	1.063
4	HG2	VAL	V	1.0201	1.0201	1.0261	1.0415	1.0789	1.0635	1.0421	1.051	1.0481	1.0694
4	N	VAL	V	120.1586	120.026	120.3733	119.5326	120.5628	119.3918	121.5251	120.9514	120.0668	119.9083

Supplementary Figure 26. Extended downloadable CSV file. The extended file includes, for each atom, the Residue ID, Residue Name, Atom ID, and the full per-frame chemical shift (CS) values across the entire trajectory.

3. Supplementary Table

	Atom type C'			Atom type CA			Atom type CB			Atom type NH			Atom type HN		
Replicate	SPARTA+	ShiftX2	UCBShift 2.0	SPARTA+	ShiftX2	UCBShift 2.0	SPARTA+	ShiftX2	UCBShift 2.0	SPARTA+	ShiftX2	UCBShift 2.0	SPARTA+	ShiftX2	UCBShift 2.0
1	0.835	0.821	0.843	0.939	0.937	0.936	0.994	0.994	0.994	0.823	0.838	0.826	0.500	0.444	0.258
2	0.834	0.815	0.829	0.939	0.937	0.927	0.994	0.994	0.994	0.834	0.849	0.820	0.515	0.451	0.193
3	0.829	0.817	0.837	0.940	0.936	0.927	0.994	0.994	0.994	0.829	0.847	0.820	0.492	0.442	0.198
4	0.828	0.811	0.840	0.939	0.936	0.936	0.995	0.994	0.994	0.823	0.844	0.833	0.472	0.419	0.278
5	0.838	0.820	0.822	0.938	0.934	0.920	0.994	0.994	0.995	0.813	0.834	0.786	0.495	0.447	0.177
6	0.824	0.813	0.821	0.936	0.934	0.930	0.994	0.994	0.994	0.834	0.848	0.817	0.493	0.460	0.202
7	0.833	0.819	0.830	0.939	0.936	0.926	0.995	0.994	0.994	0.825	0.849	0.816	0.484	0.442	0.194
8	0.832	0.822	0.861	0.940	0.938	0.936	0.995	0.994	0.994	0.829	0.847	0.868	0.475	0.436	0.302
9	0.818	0.806	0.837	0.943	0.938	0.928	0.994	0.994	0.994	0.822	0.843	0.797	0.483	0.428	0.183
10	0.822	0.803	0.823	0.941	0.938	0.926	0.995	0.994	0.994	0.827	0.846	0.813	0.508	0.465	0.202
Mean	0.829	0.815	0.834	0.939	0.936	0.929	0.994	0.994	0.994	0.826	0.845	0.820	0.492	0.443	0.219

Supplementary Table 1. Comparison of the performance of CS predictors given as Pearson correlation for the Neurotensin Type 1 receptor (see Exp ID 7 in Table 1 in the main manuscript).

Exp ID	Application	Receptor	PDB	CS signal	Purpose	Year*
1	Conformational differences in side chain rotamers	Serotonin 2b receptor	4AIQ	Cb	Case study	
2	Capturing ligand peptide binding	Bradykinin 1A receptor	7EIB	2D 13C-13C double-quantum single-quantum (DQ-SQ) spectrum	Case study	
3	Entrance gateway into GPCRs and allosteric sites	Rhodopsin receptor	5DYS	Ca	Case study	
4	GPCR activation/inactivation	μ-opioid receptor	5C1M	NH	Case study	
5	Distinguishing intrisically disordered regions & interphase contacts	*β2-adrenergic receptor*	2RH1	Backbone NH HN	Case Study	2023
6	Assigning methyl groups	Neurotensin receptor 1	6Z66	Methyl Methionine	Case study	2023
7	Benchmark different predictors (UCBShift, ShiftX2, Sparta+)	Neurotensin receptor 1	4BWB	C, Ca, Cb, NH, HN	Benchmark	2023
8	Correlation Matrix	*β1-adrenergic receptor*	4BVN	Methionine CE HE	Benchmark	2017
9	Correlation Matrix	*β1-adrenergic receptor*	4AMJ	Backbone NH HN	Benchmark	2025
10	Correlation Matrix	Adenosine A_2A receptor	4EIY	Isoleucine CD HD	Benchmark	2017
11	Correlation Matrix	*β2-adrenergic receptor*	2RH1	Backbone NH HN	Benchmark	2023
12	Correlation Matrix	Neurotensin receptor 1	4BWB	C, Ca, Cb, NH, HN	Benchmark	2023

Supplementary Table 2. Overview of case and benchmark studies. *The year refers to the publication from which the corresponding experimental data were obtained for benchmark studies. The access link to the specific analysis protocol is provided on https://github.com/GPCRmd/GPCR_Chemical_Shift/

4. References

1. Dror, R. O. *et al.* Activation mechanism of the β2-adrenergic receptor. *Proc. Natl. Acad. Sci. U. S. A.* **108**, 18684–18689 (2011).
2. Kohlhoff, K. J. *et al.* Cloud-based simulations on Google Exacycle reveal ligand modulation of GPCR activation pathways. *Nat. Chem.* **6**, 15–21 (2014).
3. Aranda-García, D. *et al.* Large scale investigation of GPCR molecular dynamics data uncovers allosteric sites and lateral gateways. *Nat. Commun.* **16**, 2020 (2025).
4. D'Amore, V. M., Conflitti, P., Marinelli, L. & Limongelli, V. Minute-timescale free-energy calculations reveal a pseudo-active state in the adenosine A2A receptor activation mechanism. *Chem* **10**, 3678–3698 (2024).
