# Supplementary material for: Integrative biophysical illumination of the 3D GPCRome dynamics
Source: Nat Struct Mol Biol. 2026 Oct 27;33(11):1733–44. doi: 10.1038/s41594-026-01891-8 (PMC13759331; doi:10.1038/s41594-026-01891-8)
Supplement: Supplementary file 2 [file 41594_2026_1891_MOESM2_ESM.pdf]

nature portfolio

Corresponding author(s): Jana Selent, Jiafei Mao

Last updated by author(s): Aug 9, 2026

Reporting Summary

Nature Portfolio wishes to improve the reproducibility of the work that we publish. This form provides structure for consistency and transparency in reporting. For further information on Nature Portfolio policies, see our Editorial Policies and the Editorial Policy Checklist.

Statistics

For all statistical analyses, confirm that the following items are present in the figure legend, table legend, main text, or Methods section.

n/a	Confirmed	
☐	☒	The exact sample size (*n*) for each experimental group/condition, given as a discrete number and unit of measurement
☐	☒	A statement on whether measurements were taken from distinct samples or whether the same sample was measured repeatedly
☐	☒	The statistical test(s) used AND whether they are one- or two-sided<br>*Only common tests should be described solely by name; describe more complex techniques in the Methods section.*
☒	☐	A description of all covariates tested
☐	☒	A description of any assumptions or corrections, such as tests of normality and adjustment for multiple comparisons
☐	☒	A full description of the statistical parameters including central tendency (e.g. means) or other basic estimates (e.g. regression coefficient) AND variation (e.g. standard deviation) or associated estimates of uncertainty (e.g. confidence intervals)
☐	☒	For null hypothesis testing, the test statistic (e.g. *F*, *t*, *r*) with confidence intervals, effect sizes, degrees of freedom and *P* value noted<br>*Give P values as exact values whenever suitable.*
☒	☐	For Bayesian analysis, information on the choice of priors and Markov chain Monte Carlo settings
☒	☐	For hierarchical and complex designs, identification of the appropriate level for tests and full reporting of outcomes
☐	☒	Estimates of effect sizes (e.g. Cohen's *d*, Pearson's *r*), indicating how they were calculated

Our web collection on statistics for biologists contains articles on many of the points above.

Software and code

Policy information about availability of computer code

Data collection	ACEMD3.3<br>SHIFTX2 (v1.10)<br>SPARTA+<br>UCBShift 2.0<br>Prime (2025.1)
Data analysis	VMD (1.9.3)<br>NGL (3.0.8)

For manuscripts utilizing custom algorithms or software that are central to the research but not yet described in published literature, software must be made available to editors and reviewers. We strongly encourage code deposition in a community repository (e.g. GitHub). See the Nature Portfolio guidelines for submitting code & software for further information.

Data

Policy information about availability of data

All manuscripts must include a data availability statement. This statement should provide the following information, where applicable:

- Accession codes, unique identifiers, or web links for publicly available datasets
- A description of any restrictions on data availability
- For clinical datasets or third party data, please ensure that the statement adheres to our policy

DATA AVAILABILITY
The GPCRmd-NMR platform is available online through the GPCRmd resource (https://www.gpcrmd.org/gpcrmd-nmr-tool). All MD simulations used as case studies in this publication can be accessed using the indicated GPCRmd submission IDs. Users can visualize simulations directly on the platform, calculate CSs, and download both the simulation trajectories and the corresponding CS data. Original data underlying this manuscript are available in the main text and its Supplementary Information. Source data are provided with this paper.

CODE AVAILABILITY
To facilitate the reproduction of the experiments, we uploaded the Jupyter-notebook files to the GPCRmd GitHub repository (https://github.com/GPCRmd/GPCR_Chemical_Shift). These notebooks contain all the code, information and steps needed to replicate the results shown in this study. The user might need to download the data from the provided links in the Jupyter-notebook and place it into the proper directories, due to the size of the files.

Research involving human participants, their data, or biological material

Policy information about studies with human participants or human data. See also policy information about sex, gender (identity/presentation), and sexual orientation and race, ethnicity and racism.

Reporting on sex and gender	n/a
Reporting on race, ethnicity, or other socially relevant groupings	n/a
Population characteristics	n/a
Recruitment	n/a
Ethics oversight	n/a

Note that full information on the approval of the study protocol must also be provided in the manuscript.

Field-specific reporting

Please select the one below that is the best fit for your research. If you are not sure, read the appropriate sections before making your selection.

☒ Life sciences ☐ Behavioural & social sciences ☐ Ecological, evolutionary & environmental sciences

For a reference copy of the document with all sections, see nature.com/documents/nr-reporting-summary-flat.pdf

Life sciences study design

All studies must disclose on these points even when the disclosure is negative.

Sample size	GPCRmd dataset: The simulation set consists of a total of 1028 simulations.<br>Case studies: 12 (see Table 1 in main manuscript)
Data exclusions	none
Replication	GPCRmd dataset: 3 replicates<br>Case studies: 3 replicates (at least)
Randomization	none
Blinding	Blinding and/or grouping is not relevant in MD simulations. MD simulations are run under specific conditions or with defined parameters. The outcomes are determined solely by the input settings and simulation protocols, not by human judgment.

Reporting for specific materials, systems and methods

We require information from authors about some types of materials, experimental systems and methods used in many studies. Here, indicate whether each material, system or method listed is relevant to your study. If you are not sure if a list item applies to your research, read the appropriate section before selecting a response.

Materials & experimental systems

n/a	Involved in the study
☒	☐ Antibodies
☐	☒ Eukaryotic cell lines
☒	☐ Palaeontology and archaeology
☒	☐ Animals and other organisms
☒	☐ Clinical data
☒	☐ Dual use research of concern
☒	☐ Plants

Methods

n/a	Involved in the study
☒	☐ ChIP-seq
☒	☐ Flow cytometry
☒	☐ MRI-based neuroimaging

Eukaryotic cell lines

Policy information about cell lines and Sex and Gender in Research

Cell line source(s)	Sf9 (Spodoptera frugiperda) insect cells were obtained from the laboratory of the Institute for Biophysical Chemistry, Goethe University Frankfurt, Germany.
Authentication	The Sf9 cell line was not independently authenticated.
Mycoplasma contamination	The Sf9 cell line was not tested for mycoplasma contamination.
Commonly misidentified lines (See ICLAC register)	The Sf9 cell line is not included in the ICLAC Register of Misidentified Cell Lines.

Plants

Seed stocks	n/a
Novel plant genotypes	n/a
Authentication	n/a
