# Supplementary material for: Integrative biophysical illumination of the 3D GPCRome dynamics
Source: Nat Struct Mol Biol. 2026 Oct 27;33(11):1733–44. doi: 10.1038/s41594-026-01891-8 (PMC13759331; doi:10.1038/s41594-026-01891-8)
Supplement: Supplementary file 3 [file 41594_2026_1891_MOESM3_ESM.pdf]

nature portfolio

Peer Review File

Integrative Biophysical Illumination of the 3D GPCRome Dynamics

Corresponding Author: Dr Jana Selent

A version of this paper was originally rejected for publication by Nature Structural & Molecular Biology, however that decision was reconsidered after appeal by the authors.

Version 0:

Decision Letter:

Nature Structural & Molecular Biology NSMB-A50850

3rd Jun 2025

Dear Dr. Selent,

Thank you for submitting your manuscript, "Integrative Biophysical Illumination of the 3D GPCRome Dynamics". Please accept our apologies for the delay in this decision. Nevertheless, the comments of 4 expert referees are below.

You will see that while reviewers express interest in the method and its utility, reviewers #2 and #4, who have the relevant technical expertise in computational biology, list several serious concerns. Specifically, they both comment on the insufficient benchmarking, which to some extent is echoed in the comments of reviewer #1. Furthermore, they also list concerns regarding the use of SHIFTX2 and SPARTA+ and the limitations of these approaches, as well as the disconnect between timescales sampled by MD and NMR, therefore questioning the physiological relevance of the insights obtained. Based on these comments, we currently cannot offer to publish the study in Nature Structural & Molecular Biology. We hope the referees' comments will be useful to you in revising the manuscript for submission elsewhere.

However, considering the strengths of the manuscript, we believe it could be a potential candidate for further consideration in Nature Communications or EMBO Press journals. Since you have opted out of transfer consultations upon submission, we have not yet contacted our editorial colleagues at these journals - I would be happy to consult my editorial colleagues there on your behalf to discuss this. Please let me know if this is something you would be interested in.

I am sincerely sorry we could not be more positive on this occasion.

Sincerely,

Katarzyna Ciazynska, PhD
(she/her)
Senior Editor
Nature Structural & Molecular Biology
https://orcid.org/0000-0002-9899-2428

Reviewer #1 (Remarks to the Author):

The authors provide a open-access platform to help predict or analyze NMR spectra from highly dynamic GPCR systems using MD simulations. The authors make the point that this framework can be used to validate spectroscopic data of dynamic ensembles and assign NMR spectra, plus predict sites for mutagenesis and subsequent detailed

NMR/FRET/DEER studies. The work strikes me as very promising and useful but I'd love to see additional validation:
1) There is a very long timescale MD simulation of A2AR (D'Amore et al., 2024, Chem 10, 3678–3698 December 12, 2024), An analysis of the frames would generate very interesting chemical shift data sets and the resulting chemical shift histograms would fall into the topic of averaging versus distinct chemical shift clusters (not averaged), since trajectory timescales span many milliseconds. It's also something not discussed (inhomogeneous versus homogeneous broadening) that the authors could explain using this data set.
2) There is also a recent publication in Science, https://www.science.org/doi/10.1126/science.adq9106, describing 81 methyl probes in a receptor. The authors have taken the trouble to assign all reporters and identify slowly exchanging states with a 4 state ensemble. This is a great milestone to be incorporated into the current GPCR-md, in the spirit of Figure 4
3) I had trouble understanding Fig 1A and the supp figures. To me, all the frames and all the 2Ds looked identical. I couldn't get a sense of the effect of averaging
4) Supp Fig 2 Caption "C atom type". Are these C' (carbonyls)
5) Supp Figs 2-6. perhaps annotate the Figs with slope and R^2 and show an aggregate? Move caption to same page
6) Supp Fig 7. Can you show the 13C shift predictions?
7) Supp Fig 8-9. It would be helpful to give an example and show the output data
8) The authors may wish to contrast this work with that used to predict chemical shifts across trajectories of intrinsically disordered proteins
9) There is no discussion of CamShift + PLUMED which predict shifts across MD trajectories

Reviewer #2 (Remarks to the Author):

Summary
In the manuscript "Integrative Biophysical Illumination of the 3D GPCRome Dynamics" by Medel-Lacruz et al, they presented GPCRmd-NMR, a novel webserver that maps conformations from molecular dynamics (MD) simulations to chemical shift (CS) predictions from available software such as ShiftX2 and SPARTA+ developed by others across the 3D proteome of GPCRs. This resulted in over 7 million in-silico GPCR conformations and over 20 billion associated in-silico chemical shift values. Both are purely in-silico values.
They then validated the ShiftX2 or SPARTA+ CS values generated on MD trajectories for B1R, NTSR1, 5-HT2B receptors to respective experimental NMR data, showing some alignments between predicted and experimental NMR data. They also outlined in Figure 3 and the discussion, potential applications such as NMR signal assignment, reporter site selection, and hypothesis generation for structural dynamics. The platform is positioned as scalable, open-access, and generalizable to other protein families and spectroscopic methods, providing pre-calculated datasets, interactive visualizations, and downloadable scripts to support broad user adoption.

However, several major concerns substantially undermine the strength of the claims.

Major Comments

1. First and foremost, the entire framework seems to rely on pre-existing chemical shift machine-learning-based predictors (ShiftX2, SPARTA+) which themselves have known limitations, particularly in modeling proton shifts and dynamic ensembles, yet the authors present their results as if these limitations can be overcome with the mass of MD conformations, without providing large-scale benchmarking of their new integrative approach against available or original experimental NMR data except for B1R and NTS1. This small-scale meta-analysis approach raises concerns.
The study will be much more appreciated if purely in-silico values (MD and CS) were mapped (e.g. by Uniprot ID) to compare with experimental CS values compiled for instance on the biological magnetic resonance data bank (BMRB). Indeed, how many experimental data points had been cumulated in the GPCRmd-NMR webserver? What percentage of data on BMRB concerns GPCR?
2. A follow-up comment. In Figure 3D, the authors suggest that machine learning scientists can "use the curated CS data for training/improving CS models." However, the chemical shift data provided are entirely in-silico predictions generated by machine-learning-based predictors (ShiftX2, SPARTA+). If one were to retrain or fine-tune models using these data, the result would be an optimization toward reproducing the outputs of ShiftX2 or SPARTA+, which are themselves sub-optimal approximations. This introduces a risk of circular validation.
3. Second, most of the analysis provided is qualitative overlays with experimental data. For instance, the NTS1R methionine studies, while experimental 2D CS values demonstrated clear centering, the in-silico prediction with MD and ShiftX2/SPARTA+ is demonstrating a spread of distribution (the blue regarding M250, Fig 3B) where the modes of computed prediction does not correspond to the experimental mode, which is unimodal, this cannot be explained as mean of the experimental observation. These issues might pertain from weaknesses of the in-silico methods, which is the corpus of data presented in the current study, without further refinements; the issue of the in-silico CS predictor were not cured by integrating MD simulation.
4. In Fig 3C. Besides, the 'membrane-exposed' pose in MD versus 'embedded-in-receptor' pose in MD should be quantified (e.g. with distance/angles/exposed volume/exposed surface area) on top of the qualitative statement, if the bimodal behavior in predicted CS values were to be explained. Statistical tests e.g. Kolmogorov Smirnov test can be used to demonstrate that the two distributions from the binarized poses are distinct, if this is the claim that the author wants to express by right-shift due to poses. Further, it is unclear how Supplementary Fig S7C juxtaposes against experimental data as the plots shown are restricted to a specific MD trajectory, not an expectation from all available MD trajectories.
5. I downloaded some of the PDB and XTC files, corresponding to several figures in this article, from GPCRmd ID: 85 for 5HT and GPCRmd ID: 872 for rhodopsin. E.g. https://gpcrmd.org/dynadb/files/Dynamics/17954_dyn_872.pdb To my surprise, in the model PDB files, the 7TM while is embedded in the lipid, there is a thick layer of "man-in-the-middle" water

blocking the 7TM's access to interact with the lipid. It seems that the system assembly may have some glitches. While due to the large size of the XTC files, I can only check into one of the trajectories, the thick layer of "man-in-the-middle" water is not present, but it still makes me wonder if the glitch is cured in all other trajectories presented?
6. Indeed, it is questionable whether the types of mechanistic insights claimed e.g. microswitch transitions, hidden ligand entry pathways, or functionally relevant conformational states, can be robustly captured by standard MD simulations limited to a few microseconds per trajectory. Without the use of enhanced sampling techniques or statistical frameworks like Markov state models, the explored conformational space is likely insufficient to draw reliable mechanistic conclusions, particularly for complex systems like GPCRs known for their slow and rare transitions. While the future is wide-open, it remains unclear how the current GPCRmd-NMR dataset has meaningfully addressed these limitations or enables deeper mechanistic understanding.

Minor Comments:

7. Fig 2A. The left-hand-side color codes does not fully correspond with the right-hand-side figure. For example, what are cyan and purple? Similarly in the SI figures.
8. Fig 2D, Tables should be taken out of the figure as a separate table. It is unclear visually what the numbers are.
9. On page 11, NTS1 and NST1 were used interchangeably for the neurotensin receptor. The acronym is complicated by additional use of NTSR1 in main text. So, which acronym is chosen?
10. On page 11, Citation for Prime Maestro should be provided.
11. On page 12, 4 femtoseconds intervals were used in the MD simulation but the author did not justify the use of this larger timestep than the commonly used 2 femtoseconds interval. Were techniques in accelerated MD were actually used? Please state in text. Besides, it is not clear what barostat/thermostat were used in the NPT and NVT simulations.

Reviewer #2 (Remarks on code availability):

It seems that the authors only provided codes to plot graphs from csv data, but not the source code where MD trajectories are analyzed and are used to compute the CS values per conformation.

Reviewer #3 (Remarks to the Author):

This manuscript introduces GPCRmd-NMR, a platform integrating molecular dynamics (MD) simulations with nuclear magnetic resonance (NMR) spectroscopy data for G protein-coupled receptors (GPCRs). The work addresses the challenge of obtaining high-resolution information on protein dynamics.

The platform contains a comprehensive dataset of predicted NMR signals mapped to GPCR conformational snapshots, enabling structure-resolved analysis at scale. The authors provide analysis tools that help interpret protein dynamics that are difficult to capture through either experimental or computational methods alone. Several case studies demonstrate the platform's utility, and the resource appears valuable for data mining and hypothesis generation.

The integration of protein dynamics and data science is timely and very relevant to current structural biology research. By providing open access to biophysical data through a web-based framework, the authors create a resource potentially useful to researchers across disciplines.

The manuscript is clearly written, methodologically sound, and contains informative and well-designed figures. The approach can extend to other protein families and biophysical data types, suggesting broader relevance beyond GPCRs.

Still, there is one point that I think needs further clarification.

The authors note in the text and Figure 2C/2D that predictions for HN atoms (amide protons) show relatively low correlations with experimental values ($R^2 = 0.42$ to 0.46),and explain that "1H chemical shifts are more sensitive to subtle inaccuracies in structural or electronic modeling." However, in subsequent analyses, the authors rely on different types of hydrogen chemical shifts - specifically methyl hydrogen shifts (HE) in Figure 3A/B for methionine side chains and valine methyl hydrogen shifts (Hγ) in Figure 4I.

The authors do not seem to clarify whether the reliability limitations observed for amide protons also apply to these other types of hydrogen atoms. The authors could address this point by either providing correlation statistics specifically for methyl hydrogen predictions if available, by explaining why methyl hydrogen predictions might be more reliable than amide hydrogen predictions, or by acknowledging any potential limitations when drawing conclusions from these hydrogen shift analyses.

Minor comments

The manuscript contains several incorrect figure references that should be corrected:

- In "Supplementary Note 1," the text states: "Figure 3C illustrates the 'Two-Dimensional Chemical Shift' mode, where D79(2.50) has slightly greater fluctuation in CS compared to D192(45x51) for the Cα and Nitrogen atoms." This appears to be an error and should reference Figure 4C instead.

- The supplementary material also contains citation inconsistencies. For example, in "Supplementary Note 1," the text mentions: "A snapshot from GPCRmd highlighting the main features of both modes is presented in Figures S4-S5." The authors should verify all supplementary figure citations for accuracy.

Reviewer #4 (Remarks to the Author):

The authors used chemical shift prediction software to generate tremendous amount of computed NMR chemical shift signals for frames in GPCR trajectories in GPCRmd, and try to align these computed chemical shifts with experimental data, and eventually use these computed chemical shifts to generate hypotheses and guide experimental designs. I have big concerns on both the methods and results of this manuscript.

1) SHIFTX2 and SPARTA+ are methods developed around 15 years ago. The training data mainly covers small soluble proteins. By then, NMR data for large membrane proteins such as GPCRs were extremely limited, so this kind of data should be scarce in the training data of the software. I assume the performance of SHIFTX2 and SPARTA+ on GPCRs may be not that reliable due to this reason. Especially,

2) The authors used a few GPCR cases (a few residues in B1R and NTSR1) to “benchmark” the accuracy of predicted chemical shifts. I think this is not enough. There are accumulated GPCR NMR data in recent years, thanks to the efforts of several research groups, such as Ichio Shimada, Stephan Grzesiek, R. Scott Prosser, and Kurt Wuthrich. I suggest the authors compare their computed chemical shifts with reports/results from these labs’ NMR research on various GPCRs. Besides, the authors did not careful analyze why HN chemical shifts cannot be accurately predicted

3) The timescale of MD simulation and NMR chemical shifts are so different that they barely overlap with each other. The timescale of MD simulation is usually at nanoseconds to microseconds, while the timescale of chemical shifts in NMR is at microseconds to milliseconds. Even though the authors calculated huge number of chemical shifts, in experiments, it’s very probably only averages of these chemical shifts can be observed. Thus, it’s very difficult to align chemical shifts from the two methods, people must be very careful to not make mistakes in hypothesis generation based on computed chemical shifts.

4) Many MD trajectories contain “broken” conformation due to the imperfectness of this method, using multiple repeats is one solution but it cannot rule out such scenarios. Given the authors do not filter these conformations out before calculating chemical shifts, wrong dynamics will be used to generate hypothesis.

5) GPCR functional conformational change (such as activation) usually happen at microseconds to milliseconds timescale. MD simulations at microsecond-timescale cannot catch such meaning conformational changes, thus chemical shifts calculated from MD simulations will lean to missing important conformational change, thus they cannot cover full energy landscape.

** As a service to authors, Springer Nature Limited provides authors with the ability to transfer a manuscript that one journal cannot offer to publish to another journal, without the author having to upload the manuscript data again. To transfer your manuscript to another NPG journal using this service, please click on Link Redacted

** For Springer Nature Limited general information and news for authors, see http://npg.nature.com/authors.

Version 1:

Decision Letter:

12th May 2026

Dear Dr. Selent,

Thank you for your letter concerning your manuscript "Integrative Biophysical Illumination of the 3D GPCRome Dynamics". Please accept my apologies for the delay in this decision. This is due to the fact that we cannot prioritise appeals over new submission in periods of high manuscript influx, and that appeals need careful discussion within the entire editorial team.

We have now had a chance to discuss the points you raised in detail, and we have decided to send your paper back to the

original reviewers. Before we do so, we ask that you provide additional documentation needed.

Please ensure to provide source data and code for review, as outlined below.

We want to ensure that the methods and statistics reporting in our papers are of the highest quality. To that end, we ask authors to fill out a Reporting Summary that collects information on experimental design and reagents.

This document can be found by following the link below:

Reporting Summary:
https://www.nature.com/documents/nr-reporting-summary.pdf

Please complete the relevant form and return it within 48 hours. Please note that these forms are dynamic 'smart pdfs' and must, therefore, be downloaded and completed in Adobe Reader. We will then flatten them for ease of use by the reviewers. If you would like to reference the guidance text as you complete the template, please access these flattened versions at http://www.nature.com/authors/policies/availability.html.

Note that you are not required to revise your paper to include the information provided in the reporting summary.

CODE AND COMPUTATIONAL WORK: If newly developed, unpublished code was used in this work, it must be provided with the revision and access to it must be disclosed in the Code Availability Statement in the manuscript. Please also provide the completed software submission checklist. This form is a dynamic 'smart pdf' and must be downloaded and completed in Adobe Reader, instead of opening it in a web browser: https://www.nature.com/documents/nr-software-policy.pdf

If molecular dynamics (MD) simulations were performed, please refer to this Editorial and provide a completed MD simulations checklist with your revision: https://www.nature.com/articles/s42003-023-04653-0

Lastly, if it applies, please complete and upload the completed machine learning checklist with your submission for re-review: https://www.nature.com/documents/machine-learning-checklist.pdf

SOURCE GEL IMAGES: Unprocessed scans should be provided for all gels and western blots presented in figures and should be clearly labelled with the figure number in the file title. The source gel images should be uncropped, unmodified images, with molecular weight markers. Uncropped, unmodified gel images from existing experimental repeats, in addition to the data shown in the figures, can also be presented in source data. Please provide these files as PDF with your revision.

Please ensure that all control panels for gels and western blots are appropriately described as loading or IP controls. Lastly, please ensure that all images in the paper are checked for duplication of panels and for splicing of gel lanes.

SOURCE NUMERICAL DATA: we urge authors to provide, in tabular form in excel files, the data underlying the graphical representations used in figures. This is to further increase transparency in data reporting, as detailed in this editorial (http://www.nature.com/nsmb/journal/v22/n10/full/nsmb.3110.html). Please provide one excel file per figure max, with one panel per tab. When submitting files, the title field should indicate which figure the source data pertains to.

DATA AVAILABILITY: this journal strongly supports public availability of data. All data used in accepted papers should be available via a public data repository, or alternatively, as Supplementary Information. If data can only be shared on request, please explain why in your Data Availability Statement and in the correspondence with your editor.

For some data types, deposition in a public repository is mandatory as detailed below:

https://www.nature.com/nature-research/editorial-policies/reporting-standards#availability-of-data

Finally, please ensure that you retain unprocessed data and metadata files after publication, ideally archiving data in perpetuity, as these may be requested during the peer review and production process or after publication if any issues arise.

EXTENDED DATA FIGURES

When re-submitting your manuscript, please ensure that any supplementary figures and tables that are crucial to the manuscript's conclusions are converted into Extended Data figures and tables to increase visibility of these data. Extended

Data figures and tables are online-only (present in the online PDF and full-text HTML versions of the paper), peer-reviewed display items that provide essential background to the article but are not included in the main article due to space constraints. A maximum of ten Extended Data display items (figures and tables) is permitted.

While we encourage the use of color in preparing figures, please note that this will incur a charge to partially defray the cost of printing. Information about color charges can be found at http://www.nature.com/nsmb/authors/submit/index.html#costs

Once we receive this document and review it to ensure that all requested information is provided, we will proceed to send your paper for review. If you have questions or anticipate delays, please let me know as soon as possible.

You can use the link below to be taken directly to the site and submit your manuscript:

Link Redacted

Sincerely,

Katarzyna Ciazynska, PhD
(she/her)
Senior Editor
Nature Structural & Molecular Biology
https://orcid.org/0000-0002-9899-2428

Version 2:

Decision Letter:

Our ref: NSMB-RS50850B

22nd Jun 2026

Dear Dr. Selent,

Thank you for submitting your revised manuscript "Integrative Biophysical Illumination of the 3D GPCRome Dynamics" (NSMB-RS50850B). It has now been seen by the original referees and their comments are below. The reviewers find that the paper has improved in revision, and therefore we'll be happy in principle to publish it in Nature Structural & Molecular Biology, pending minor revisions to satisfy the referees' final requests and to comply with our editorial and formatting guidelines.

We ask that you clearly state limitations as outlined by the reviewers. We have changed the article format to Resource, as we believe it better represents the study. Please let us know is you have any concerns or questions about this.

We are now performing detailed checks on your paper and will send you a checklist detailing our editorial and formatting requirements in a couple of weeks. Please do not upload the final materials and make any revisions until you receive this additional information from us.

Thank you again for your interest in Nature Structural & Molecular Biology Please do not hesitate to contact me if you have any questions.

Sincerely,

Katarzyna Ciazynska, PhD
(she/her)
Senior Editor
Nature Structural & Molecular Biology
https://orcid.org/0000-0002-9899-2428

Reviewer #1 (Remarks to the Author):

I found the additional benchmarks quite useful
The new paper reads well and my questions were answered.

It's strength lies in the open source platform for others to use and contribute to.

Reviewer #2 (Remarks to the Author):

I thank the authors for their detailed rebuttal and for the substantial revisions made to the manuscript. The additional benchmark against experimental BMRB datasets, the removal of the circular-validation claim, the new quantitative analyses, and the expanded discussion have addressed the major concerns raised in my original review. In my opinion, the manuscript has improved significantly and is now suitable for publication after minor revision.

I have only two remaining points that should be addressed before acceptance:

1. Avoid claims of MD convergence based solely on agreement with experimental chemical shifts.

The revised manuscript appears to imply that agreement between predicted and experimental chemical shifts demonstrates that the underlying MD ensemble is well converged. I do not believe such a conclusion is warranted. Agreement with one or a limited set of observables can indicate consistency with experiment, but it does not by itself establish convergence of the ensemble. I recommend removing or softening any statements that suggest experimental agreement proves MD convergence, and instead describing the agreement as supportive evidence for the relevance of the sampled conformations.

2. Explicitly state the limited availability of experimental GPCR NMR datasets.

The authors have now performed a valuable exercise against all GPCR datasets currently available in the BMRB and report that only five GPCR cases were identified, yielding 679 chemical-shift measurements. This is an important contextual point that helps readers understand the limitations of experimental validation in this field. I recommend that the manuscript explicitly state, in the main text and/or discussion, that the benchmark was performed against all currently available GPCR entries in the BMRB (five cases, 679 chemical-shift values), rather than leaving this information only in the response to reviewers or supplementary material.

Subject to these minor revisions, I support acceptance of the manuscript.

Reviewer #3 (Remarks to the Author):

I have read the revised manuscript and the authors' responses to my previous comments.
My main concern in the first round was the reliability of the 1H chemical shift predictions. The authors have addressed this point satisfactorily. I appreciate that the authors also examined the available BMRB records for methyl protons and acknowledged transparently that the limited number of datapoints precludes a statistically meaningful correlation. I am satisfied that my comments have been resolved, and I have no further substantive concerns.

Reviewer #4 (Remarks to the Author):

This manuscript presents a comprehensive study bridging MD simulations and NMR analyses of GPCRs. The concerns raised in the previous round of review have been satisfactorily addressed, and the revised manuscript is now well developed. I have only a few minor comments:
1. The first letter of the Latin name for rat should be capitalized.
Page 4; or Line 137, Page 35 in the original version.
2. PDB 4IAQ is listed as a structure of 5-HT2BR on Pages 5 and 24; however, it corresponds to 5-HT1BR. Please correct this assignment.
3. For 5-HT2B, the key residue is referred to as F311^6x52 in the main text on Page 5, but as F331^6.52 in Figure 2D on Page 23 and on Page 24. Please verify the residue number and correct the Ballesteros–Weinstein numbering label accordingly.
4. Terminology should be made consistent throughout the manuscript.
For adrenergic receptors, different forms are used, such as “β_2 adrenergic receptor,” “β2 adrenergic receptor,” and “β2-adrenergic receptor.” Please use one consistent format.
For neurotensin receptor 1, both “NTSR1” and “NTR1” are used as abbreviations. Please standardize the abbreviation.
5. Please correct the use and formatting of Greek letters.
The notation for side-chain dihedral angles should use the lowercase Greek letter χ, rather than the Latin letter X, in Figure 2D.
The fonts of α, β, and γ in the right panel of Figure 2A, and α in Figure 2C, appear inconsistent with those used elsewhere in

the manuscript. Please standardize the formatting.

INTERNAL USE ONLY AIP modules required: ["Life Science"]

Version 3:

Decision Letter:

24th Aug 2026

Dear Dr. Selent,

We are now happy to accept your revised paper "Integrative Biophysical Illumination of the 3D GPCRome Dynamics" for publication as a Resource in Nature Structural & Molecular Biology.

Acceptance is conditional on the manuscript's not being published elsewhere and on there being no announcement of this work to the newspapers, magazines, radio or television until the publication date in Nature Structural & Molecular Biology.

Over the next few weeks, your paper will be copyedited to ensure that it conforms to Nature Structural & Molecular Biology style. Once your paper is typeset, you will receive an email with a link to choose the appropriate publishing options for your paper and our Author Services team will be in touch regarding any additional information that may be required.

After the grant of rights is completed, you will receive a link to your electronic proof via email with a request to make any corrections within 48 hours. If, when you receive your proof, you cannot meet this deadline, please inform us at rjsproduction@springernature.com immediately.

You will not receive your proofs until the publishing agreement has been received through our system.

Due to the importance of these deadlines, we ask that you please let us know now whether you will be difficult to contact over the next month. If this is the case, we ask you provide us with the contact information (email, phone and fax) of someone who will be able to check the proofs on your behalf, and who will be available to address any last-minute problems.

To assist our authors in disseminating their research to the broader community, our SharedIt initiative provides all co-authors with the ability to generate a unique shareable link that will allow anyone (with or without a subscription) to read the published article. Recipients of the link with a subscription will also be able to download and print the PDF.

As soon as your article is published, you can generate your shareable link by entering the DOI of your article here: http://authors.springernature.com/share. Corresponding authors will also receive an automated email with the shareable link

Note the policy of the journal on data deposition: http://www.nature.com/authors/policies/availability.html.

Your paper will be published online soon after we receive proof corrections and will appear in print in the next available issue. You can find out your date of online publication by contacting the production team shortly after sending your proof corrections.

You may wish to make your media relations office aware of your accepted publication, in case they consider it appropriate to organize some internal or external publicity. Once your paper has been scheduled you will receive an email confirming the publication details. This is normally 3-4 working days in advance of publication. If you need additional notice of the date and time of publication, please let the production team know when you receive the proof of your article to ensure there is sufficient time to coordinate. Further information on our embargo policies can be found here: https://www.nature.com/authors/policies/embargo.html

You can now use a single sign-on for all your accounts, view the status of all your manuscript submissions and reviews, access usage statistics for your published articles and download a record of your refereeing activity for the Nature journals.

If you have not already done so, we strongly recommend that you upload the step-by-step protocols used in this manuscript to the Protocol Exchange. Protocol Exchange is an open online resource that allows researchers to share their detailed experimental know-how. All uploaded protocols are made freely available, assigned DOIs for ease of citation and fully searchable through nature.com. Protocols can be linked to any publications in which they are used and will be linked to from your article. You can also establish a dedicated page to collect all your lab Protocols. By uploading your Protocols to Protocol Exchange, you are enabling researchers to more readily reproduce or adapt the methodology you use, as well as increasing the visibility of your protocols and papers. Upload your Protocols at www.nature.com/protocolexchange/. Further

information can be found at www.nature.com/protocolexchange/about.

An online order form for reprints of your paper is available at https://www.nature.com/reprints/author-reprints.html. Please let your coauthors and your institutions' public affairs office know that they are also welcome to order reprints by this method.

Authors may need to take specific actions to achieve compliance with funder and institutional open access mandates. If your research is supported by a funder that requires immediate open access (e.g. according to Plan S principles or the NIH public access policy) then you should select the gold OA route, and we will direct you to the compliant route where possible. Because authors warrant under our subscription licensing terms that they haven't committed to licensing any version of their article under a licence inconsistent with the terms of our agreement – including the applicable embargo period – publication under the subscription model isn't suitable for authors whose funders require no embargo.

In approximately 10 business days you will receive an email with a link to choose the appropriate publishing options for your paper and our Author Services team will be in touch regarding any additional information that may be required.

You will not receive your proofs until the publishing agreement has been received through our system.

If you have any questions about our publishing options, costs, Open Access requirements, or our legal forms, please contact ASJournals@springernature.com

Sincerely,

Katarzyna Ciazynska, PhD
(she/her)
Senior Editor
Nature Structural & Molecular Biology
https://orcid.org/0000-0002-9899-2428

Click here if you would like to recommend Nature Structural & Molecular Biology to your librarian:
http://www.nature.com/subscriptions/recommend.html#forms

Open Access This Peer Review File is licensed under a Creative Commons Attribution 4.0 International License, which permits use, sharing, adaptation, distribution and reproduction in any medium or format, as long as you give appropriate credit to the original author(s) and the source, provide a link to the Creative Commons license, and indicate if changes were made.
In cases where reviewers are anonymous, credit should be given to 'Anonymous Referee' and the source.
The images or other third party material in this Peer Review File are included in the article's Creative Commons license,

unless indicated otherwise in a credit line to the material. If material is not included in the article's Creative Commons license and your intended use is not permitted by statutory regulation or exceeds the permitted use, you will need to obtain permission directly from the copyright holder.
To view a copy of this license, visit https://creativecommons.org/licenses/by/4.0/

We thank the reviewers for their time, dedication, and constructive comments. We greatly appreciate the thoughtful feedback, which has helped us to improve the quality and clarity of the manuscript. All comments have been comprehensively addressed in the revised version.

Reviewer #1 (Remarks to the Author):

The authors provide an open-access platform to help predict or analyze NMR spectra from highly dynamic GPCR systems using MD simulations. The authors make the point that this framework can be used to validate spectroscopic data of dynamic ensembles and assign NMR spectra, plus predict sites for mutagenesis and subsequent detailed NMR/FRET/DEER studies. The work strikes me as very promising and useful but I'd love to see additional validation:

R1.1 There is a very long timescale MD simulation of A2AR (D'Amore et al., 2024, Chem 10, 3678–3698 December 12, 2024), An analysis of the frames would generate very interesting chemical shift data sets and the resulting chemical shift histograms would fall into the topic of averaging versus distinct chemical shift clusters (not averaged), since trajectory timescales span many milliseconds. It's also something not discussed (inhomogeneous versus homogeneous broadening) that the authors could explain using this data set.

We thank the reviewer for directing us to this very valuable data. We have now included this dataset in our analysis as suggested by the reviewer.

First, we show that the GPCRmd-NMR framework is able to detect partial inactivation transitions in the A2AR as described in D'Amore et al. Specifically, we demonstrate how a TM6 Hydrogen-Bond rearrangement induces alterations in CS values during the transition from active to preactive states (**Supplementary Figure 24**).

Second, we address the issue of "averaging versus distinct chemical shift clusters" in a completely new section entitled "Bridging the MD-NMR gap at distinct time scales". In this section, we describe strategies for translating computed chemical shifts into NMR observables, explicitly accounting for both slow- and fast-exchange regimes in NMR. This framework can also be applied to the A2AR MD simulations from D'Amore et al. Notably, we observe clear differential state signals (**Supplementary Figure 24E**), which would correspond to distinct signals in solid-state NMR experiments. We further compute the average chemical shifts (120.43 ppm), which corresponds to a population mixture of 30% active and 70% preactive states, as sampled during the 5-μs unbiased MD simulation (120.4 ppm).

Third, the line broadening is an important aspect. We thank the reviewer for bringing this to our attention. The NMR linebroadening/shape actually comes from both the stochastic ("relaxation") and coherent contributions. Therefore, the observed NMR lineshape may not be directly mapped to MD histograms. In the coherent part, homogeneous broadening is often caused by residual homonuclear (mostly ^1H-^1H) dipolar couplings (e.g., proteoliposome samples or cellular preparations for solid-state NMR), whereas inhomogeneous broadening arises, for instance, from chemical shift distribution. Nevertheless, at low temperatures, where the samples are frozen, the stochastic T_2 relaxation does not dominate the lineshape. The solid-state NMR signals of such frozen samples could reflect the CS distribution if the decoupling is sufficient. In this case, we do see a good match between the MD-derived CS histogram and real-world NMR spectra (see **Figure 2A** in the main manuscript). In solution NMR at room/physiologically relevant temperatures, the lineshape could be dominated by relaxation and the interference between the (stochastic) conformational dynamics and the (coherent) NMR interactions, e.g., chemical shift (differences). The latter case is now intensively discussed in the revised version thanks to the relevant advice from reviewers (see methionine conformational switching in the NTR1, **Figure 3**).

R1.2 There is also a recent publication in Science, https://www.science.org/doi/10.1126/science.adq9106, describing 81 methyl probes in a receptor. The authors have taken the trouble to assign all reporters and identify slowly exchanging states with a 4 state ensemble. This is a great milestone to be incorporated into the current GPCR-md, in the spirit of Figure 4

Thank you for bringing this important study to our attention. We have now incorporated this case as an additional benchmark for the chemical-shift predictors evaluated in our work. A comprehensive table summarizing all benchmarking datasets, including this new case (**experiment ID 9**), is provided in **Table 1**. The correlation results for all newly added benchmarks are reported in **Supplementary Figure S17**.

R1.3 I had trouble understanding Fig 1A and the supp figures. To me, all the frames and all the 2Ds looked identical. I couldn't get a sense of the effect of averaging

Thank you for this comment. **Figure 1A** illustrates the GPCRmd-NMR analysis pipeline. We suspect that the reviewer may instead be referring to **Figure 2A** and the corresponding **Supplementary Figure 1**.

To improve clarity, we have modified the color scheme in the bradykinin example to highlight only the chemical shift signals corresponding to the selected atoms in the proline. The same color coding has been applied consistently to the supplementary figures (**Supplementary Figure 1**).

R1.4 Supp Fig 2 Caption "C atom type". Are these C' (carbonyls)

Thank you for pointing this out. We have clarified the terminology in the main text and updated the figures to explicitly indicate that these correspond to C′ (carbonyl) atoms.

R1.5 Supp Figs 2-6. perhaps annotate the Figs with slope and R^2 and show an aggregate? Move caption to same page

Thank you for the suggestion. We have incorporated the correlation and the RMSE values directly on each plot in **Figure 2C**. The R^2 and the slope are now provided in **Supplementary Figures 2–16**, where a detailed table is added for each atom type summarizing all the replicates. Furthermore, we have included a comprehensive table (**Supplementary Table 1**) compiling all correlation metrics. For better accessibility, figure captions are now positioned on the same page as the corresponding plots, which have been redesigned for enhanced visual clarity.

R1.6 Supp Fig 7. Can you show the 13C shift predictions?

We thank the reviewer for this comment. We have removed the supplementary figure from the manuscript and added new benchmarking case studies derived from BMRB readouts to assess the overall accuracy of the ^{13}C shift predictions (**Table 1**). Our benchmark study confirms high predictive performance across different types of carbons within correlations ranging from 0.819 to 0.994 (**Supplementary Figure 17**).

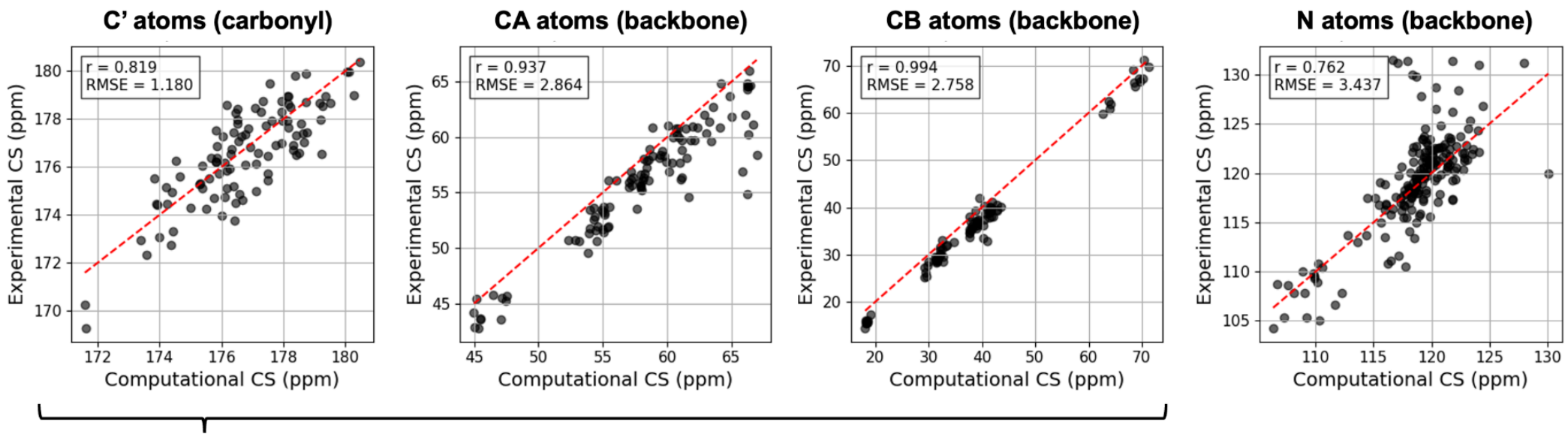

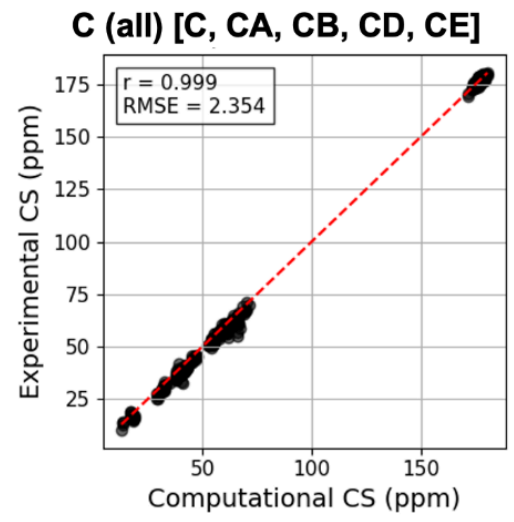

Atom type	Pearson r	RMSE	Exp. data points	Experiment IDs
C' (carbonyl)	0.819	1.180	101	12
CA	0.937	2.864	106	12
CB	0.994	2.758	86	12
N	0.762	3.437	188	9, 11, 12
H	0.321	0.780	188	9, 11, 12
C (all)	0.999	2.354	303	8, 9, 10, 11, 12

Supplementary Figure 17. Benchmarking of GPCRmd-NMR CS prediction against large-scale NMR experimental datasets. The table presents the benchmarking results of GPCRmd-NMR CS predictions against experimental data from diverse studies (IDs 8–12). All experimental datasets were combined into a single correlation matrix, and the performance of the CS predictor ShiftX2 was evaluated across different atom types. The reported number of experimental data points corresponds to the total CS signals obtained from studies with IDs 8–12. Detailed information about each receptor system included in these experiments (Experiment ID) is provided in Table 1.

Finally, we also individually examined the ^{13}C chemical shift distribution of methionine 250 as requested by the reviewer. The predicted mean ^{13}C chemical shift for M250 is 17.3 ppm, in close agreement with the experimental value of 17.7 ppm. However, despite this apparent agreement, we chose not to present or further discuss this signal because the overall prediction error for ^{13}C chemical shifts remains relatively high (RMSE = 2.35 ppm for all carbons; see Supplementary Figure 17) compared with the predicted ^{13}C chemical shift distribution (~0.5 ppm). In contrast, the predicted ^{1}H chemical shift distribution of this methyl group (> 1.0 ppm) is substantially larger than the global ^{1}H RMSE (0.8 ppm). We therefore focused on the ^{1}H dimension for the analysis presented here.

R1.7 Supp Fig 8-9. It would be helpful to give an example and show the output data

Thank you for this great suggestion. We have now added two examples showing the first lines of the downloadable output files in **Supplementary Figures 26 and 27**.

Additionally, we have included a few example files on GitHub (https://github.com/GPCRmd/GPCR_Chemical_Shift/tree/main/CS_computation/example).

R1.8 The authors may wish to contrast this work with that used to predict chemical shifts across trajectories of intrinsically disordered proteins

Thank you for highlighting this important application. We have incorporated a dedicated section to the manuscript entitled "**Exploring intrinsically disordered protein regions and interface contacts.**" In this section, we analyze MD trajectories of the β2-adrenergic receptor C-terminal region from Heng et al. (*Nat. Commun.*, 2023), demonstrating how CS predictors capture

order–disorder transitions and receptor-IDR (intrinsic disordered region) interactions through chemical shifts. These findings are presented in a new figure (**Figure 4**).

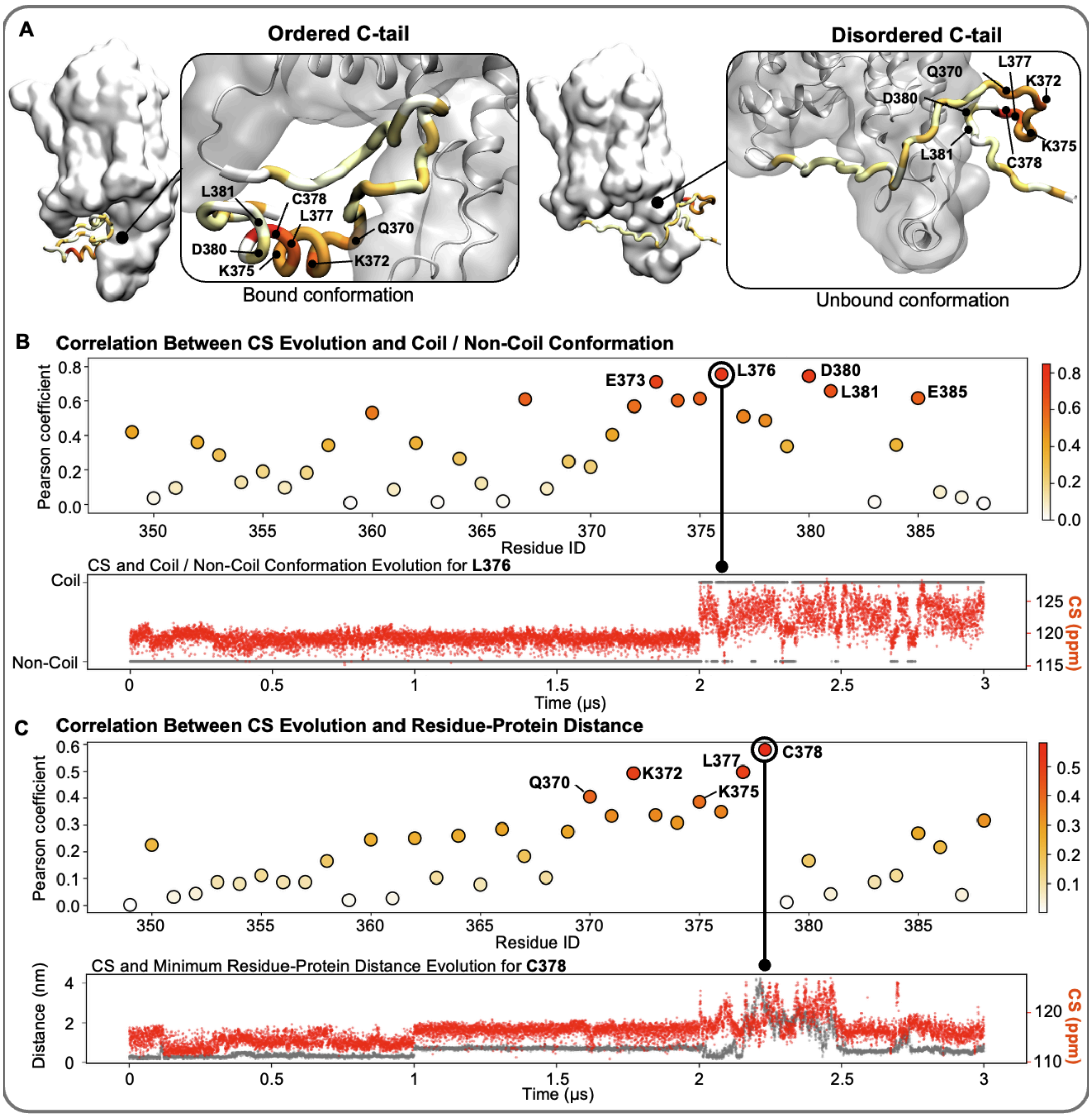

Figure 4. Chemical shift correlations reveal disorder, ordering, and transient receptor contacts in the β₂AR C-tail (CT). (**A**) Representative conformations of β₂AR CT MD simulations from Heng et al. for the ordered CT and the disordered CT. (**B**) Pearson correlation between the time-evolution of predicted NH chemical shifts (CS) for each CT residue and its coil versus non-coil classification. Residues within the helix-forming segment (368–378) show the strongest correlations (L376 shown as an example). (**C**) Correlation between NH CS evolution and residue–receptor proximity, highlighting residues whose CS values are strongly modulated by transient contacts with the intracellular receptor core (example shown for C378).

R1.9 There is no discussion of CamShift + PLUMED which predict shifts across MD trajectories
We thank the reviewer for bringing this point to our attention. We have added a note regarding additional chemical shift prediction tools, such as **CamShift** (now cited in the manuscript with reference 43), that can be integrated into the GPCRmd-NMR framework. As part of our ongoing efforts to expand the platform, we have already incorporated **UCBShift 2.0** (see Reviewer #4, point **R4.1** and Reviewer #2, point **R2.1.1**). We also plan to include **CamShift** in the next server release to broaden further the range of biophysical data that can be analyzed.

Reviewer #2 (Remarks to the Author):

Summary

In the manuscript “Integrative Biophysical Illumination of the 3D GPCRome Dynamics” by Medel-Lacruz et al, they presented GPCRmd-NMR, a novel webserver that maps conformations from molecular dynamics (MD) simulations to chemical shift (CS) predictions from available software such as ShiftX2 and SPARTA+ developed by others across the 3D proteome of GPCRs. This resulted in over 7 million in-silico GPCR conformations and over 20 billion associated in-silico chemical shift values. Both are purely in-silico values.

They then validated the ShiftX2 or SPARTA+ CS values generated on MD trajectories for B1R, NTSR1, 5-HT2B receptors to respective experimental NMR data, showing some alignments between predicted and experimental NMR data. They also outlined in Figure 3 and the discussion, potential applications such as NMR signal assignment, reporter site selection, and hypothesis generation for structural dynamics. The platform is positioned as scalable, open-access, and generalizable to other protein families and spectroscopic methods, providing pre-calculated datasets, interactive visualizations, and downloadable scripts to support broad user adoption.

However, several major concerns substantially undermine the strength of the claims.

Major Comments

R2.1 First and foremost, the entire framework seems to rely on pre-existing chemical shift machine-learning-based predictors (ShiftX2, SPARTA+) which themselves have known limitations, particularly in modeling proton shifts and dynamic ensembles, yet the authors present their results as if these limitations can be overcome with the mass of MD conformations, without providing large-scale benchmarking of their new integrative approach against available or original experimental NMR data except for B1R and NTS1. This small-scale meta-analysis approach raises concerns.

The study will be much more appreciated if purely in-silico values (MD and CS) were mapped (e.g. by Uniprot ID) to compare with experimental CS values compiled for instance on the biological magnetic resonance data bank (BMRB). Indeed, how many experimental data points had been cumulated in the GPCRmd-NMR webserver? What percentage of data on BMRB concerns GPCR?

We thank the reviewer for valuable suggestions that are addressed below:

R2.1.1 The entire framework seems to rely on pre-existing chemical shift machine-learning-based predictors (ShiftX2, SPARTA+)

We have incorporated the recently released **UCBShift 2.0 (2024)** as an additional CS predictor to the GPCRmd NMR framework. This new predictor demonstrates comparable performance to SPARTA+ and ShiftX2, as shown in **Supplementary Table 1**.

	Atom type C'			Atom type CA			Atom type CB			Atom type NH			Atom type HN		
**Replicate**	**SPARTA+**	**ShiftX2**	**UCBShift 2.0**	**SPARTA+**	**ShiftX2**	**UCBShift 2.0**	**SPARTA+**	**ShiftX2**	**UCBShift 2.0**	**SPARTA+**	**ShiftX2**	**UCBShift 2.0**	**SPARTA+**	**ShiftX2**	**UCBShift 2.0**
**1**	0.835	0.821	0.843	0.939	0.937	0.936	0.994	0.994	0.994	0.823	0.838	0.826	0.500	0.444	0.258
**2**	0.834	0.815	0.829	0.939	0.937	0.927	0.994	0.994	0.994	0.834	0.849	0.820	0.515	0.451	0.193
**3**	0.829	0.817	0.837	0.940	0.936	0.927	0.994	0.994	0.994	0.829	0.847	0.820	0.492	0.442	0.198
**4**	0.828	0.811	0.840	0.939	0.936	0.936	0.995	0.994	0.994	0.823	0.844	0.833	0.472	0.419	0.278
**5**	0.838	0.820	0.822	0.938	0.934	0.920	0.994	0.994	0.995	0.813	0.834	0.786	0.495	0.447	0.177
**6**	0.824	0.813	0.821	0.936	0.934	0.930	0.994	0.994	0.994	0.834	0.848	0.817	0.493	0.460	0.202
**7**	0.833	0.819	0.830	0.939	0.936	0.926	0.995	0.994	0.994	0.825	0.849	0.816	0.484	0.442	0.194
**8**	0.832	0.822	0.861	0.940	0.938	0.936	0.995	0.994	0.994	0.829	0.847	0.868	0.475	0.436	0.302
**9**	0.818	0.806	0.837	0.943	0.938	0.928	0.994	0.994	0.994	0.822	0.843	0.797	0.483	0.428	0.183
**10**	0.822	0.803	0.823	0.941	0.938	0.926	0.995	0.994	0.994	0.827	0.846	0.813	0.508	0.465	0.202
Mean	0.829	0.815	0.834	0.939	0.936	0.929	0.994	0.994	0.994	0.826	0.845	0.820	0.492	0.443	0.219

Supplementary Table 1. Comparison of the performance of CS predictors given as Pearson correlation for the Neurotensin Type 1 receptor (see **Exp ID 7 in Table 1** in the main manuscript and **R2.1.2**).

R2.1.2 The study will be much more appreciated if purely in-silico values (MD and CS) were mapped (e.g. by Uniprot ID) to compare with experimental CS values compiled for instance on the biological magnetic resonance data bank (BMRB). How many experimental data points had been cumulated in the GPCRmd-NMR webserver? What percentage of data on BMRB concerns GPCR?

To strengthen the GPCRmd-NMR framework, we compiled a list of all available GPCR NMR studies from the BMRB (see **Table 1**, example IDs 8–12). In total, 5 GPCR cases were found in the BMRB, resulting in 679 chemical shift values.

Exp ID	Application	Receptor	PDB	CS signal	Purpose	Year*
1	Conformational differences in side chain rotamers	Serotonin 2b receptor	4AIQ	Cb	Case study	
2	Capturing ligand peptide binding	Bradykinin 1A receptor	7EIB	2D 13C-13C double-quantum single-quantum (DQ-SQ) spectrum	Case study	
3	Entrance gateway into GPCRs and allosteric sites	Rhodopsin receptor	5DYS	Ca	Case study	
4	GPCR activation/inactivation	μ-opioid receptor	5C1M	NH	Case study	
5	Detection of intrinsically disordered regions & interphase contacts	Beta-2 adrenergic receptor	2RH1	Backbone NH HN	Case study	2023
6	Assigning methyl groups	Neurotensin receptor 1	6Z66	Methyl Methionine	Case study	2023
7	Benchmark different predictors (UCBShift, ShiftX2, Sparta+)	Neurotensin receptor 1	4BWB	C, Ca, Cb, NH, HN	Benchmark	2023
8	Correlation Matrix (BMRB 27292)	Beta-1 adrenergic receptor	4BVN	Methionine CE HE	Benchmark	2017
9	Correlation Matrix (BMRB 52783)	Beta-1 adrenergic receptor	4AMJ	Backbone NH HN	Benchmark	2025
10	Correlation Matrix (BMRB 27261)	Adenosine A_2A receptor	4EIY	Isoleucine CD HD	Benchmark	2017
11	Correlation Matrix (BMRB 51648)	Beta-2 adrenergic receptor	2RH1	Backbone NH HN	Benchmark	2023
12	Correlation Matrix (BMRB 51907)	Neurotensin receptor 1	4BWB	C', Ca, Cb, NH, HN	Benchmark	2023

An overview of their performance, including the number of experimental data points, is summarized in **Supplementary Figure 17**. Overall, the correlations are strong, with the anticipated exception of 1H chemical shift predictions.

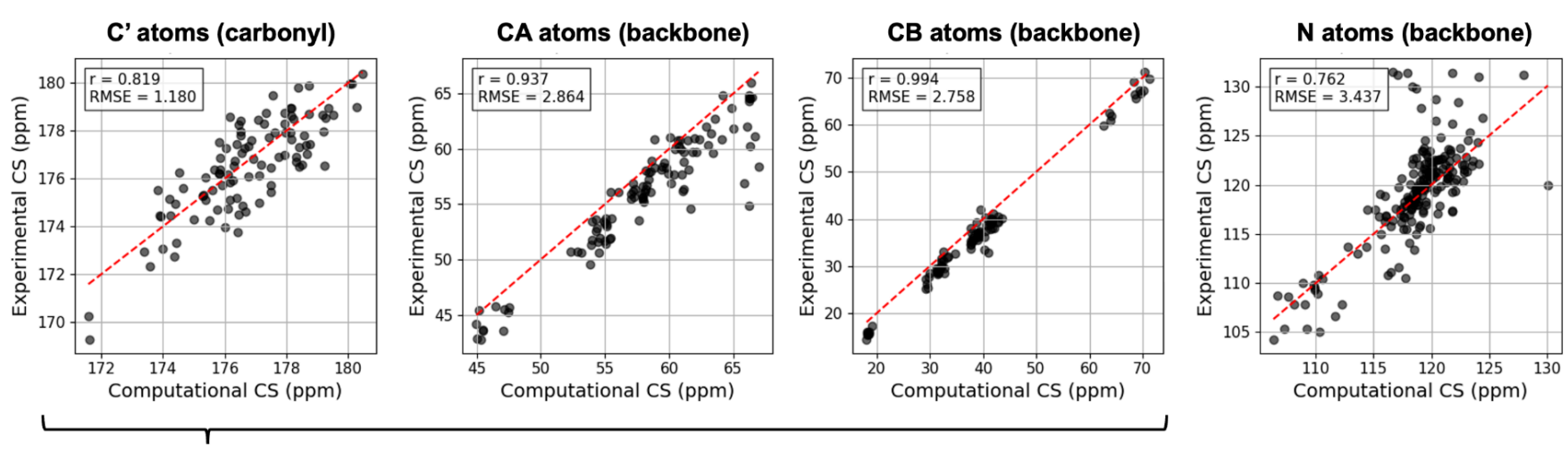

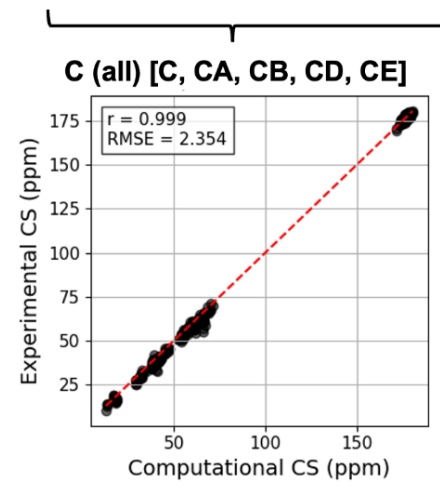

Atom type	Pearson r	RMSE	Exp. data points	Experiment IDs
C' (carbonyl)	0.819	1.180	101	12
CA	0.937	2.864	106	12
CB	0.994	2.758	86	12
N	0.762	3.437	188	9, 11, 12
H	0.321	0.780	188	9, 11, 12
C (all)	0.999	2.354	303	8, 9, 10, 11, 12

Supplementary Figure 17. Benchmarking of GPCRmd-NMR CS prediction against NMR experimental datasets. The table presents the benchmarking results of GPCRmd-NMR CS predictions against experimental data from diverse studies (IDs 8–12). All experimental datasets were combined into a single correlation matrix, and the performance of the CS predictor ShiftX2 was evaluated across different atom types. The reported number of experimental data points corresponds to the total CS signals obtained from studies with IDs 8–12. Detailed information about each receptor system included in these experiments (Experiment ID) is provided in Table 1.

R2.2 A follow-up comment. In Figure 3D, the authors suggest that machine learning scientists can "use the curated CS data for training/improving CS models." However, the chemical shift data provided are entirely in-silico predictions generated by machine-learning-based predictors (ShiftX2, SPARTA+). If one were to retrain or fine-tune models using these data, the result would be an optimization toward reproducing the outputs of ShiftX2 or SPARTA+, which are themselves sub-optimal approximations. This introduces a risk of circular validation.

We agree with the reviewer's concern regarding the risk of circular validation. In light of this, we have removed the statement suggesting that the curated CS data could be used to train or improve machine-learning models, in the previous **Figure 3D**, now in **Figure 7**.

R2.3 Second, most of the analysis provided is qualitative overlays with experimental data. For instance, the NTS1R methionine studies, while experimental 2D CS values demonstrated clear centering, the in-silico prediction with MD and ShiftX2/SPARTA+ is demonstrating a spread of distribution (the blue regarding M250, Fig 3B) where the modes of computed prediction does not correspond to the experimental mode, which is unimodal, this cannot be explained as mean of the experimental observation. These issues might pertain from weaknesses of the in-silico methods, which is the corpus of data presented in the current study, without further refinements; the issue of the in-silico CS predictor were not cured by integrating MD simulation.

We thank the reviewer for pointing out this discrepancy. We acknowledge that this section needed additional clarification.

Our MD and CS predictions suggest that the experimentally observed methyl chemical shift for M250 (**Figure 3A**, ~1.66 ppm) is an average value over diverse conformational subpopulations (**Figure 3B**) that interconvert rapidly (i.e., within the fast-exchange regime on the NMR timescale). To facilitate interpretation, we added the computed average CS values over the conformational population distribution for replicates 1–3 individually (**Figure 3B**), as well as the combined average across all three replicates (1.71 ppm) (**Figure 3A** in blue). The combined average closely matches the experimental NMR observable (~1.66 ppm; **Figure 3A**). Importantly, the averaged CS derived from our MD ensemble reflects the relative weighting of individual methyl populations. For example, the average CS shifts toward lower values when membrane-exposed conformations are more populated (e.g., replicate 2). Most importantly, the close agreement between the combined CS average and the experimental NMR signal also indicates that the MD-sampled ensemble is well converged and closely approximates the conformational distribution expected under experimental conditions.

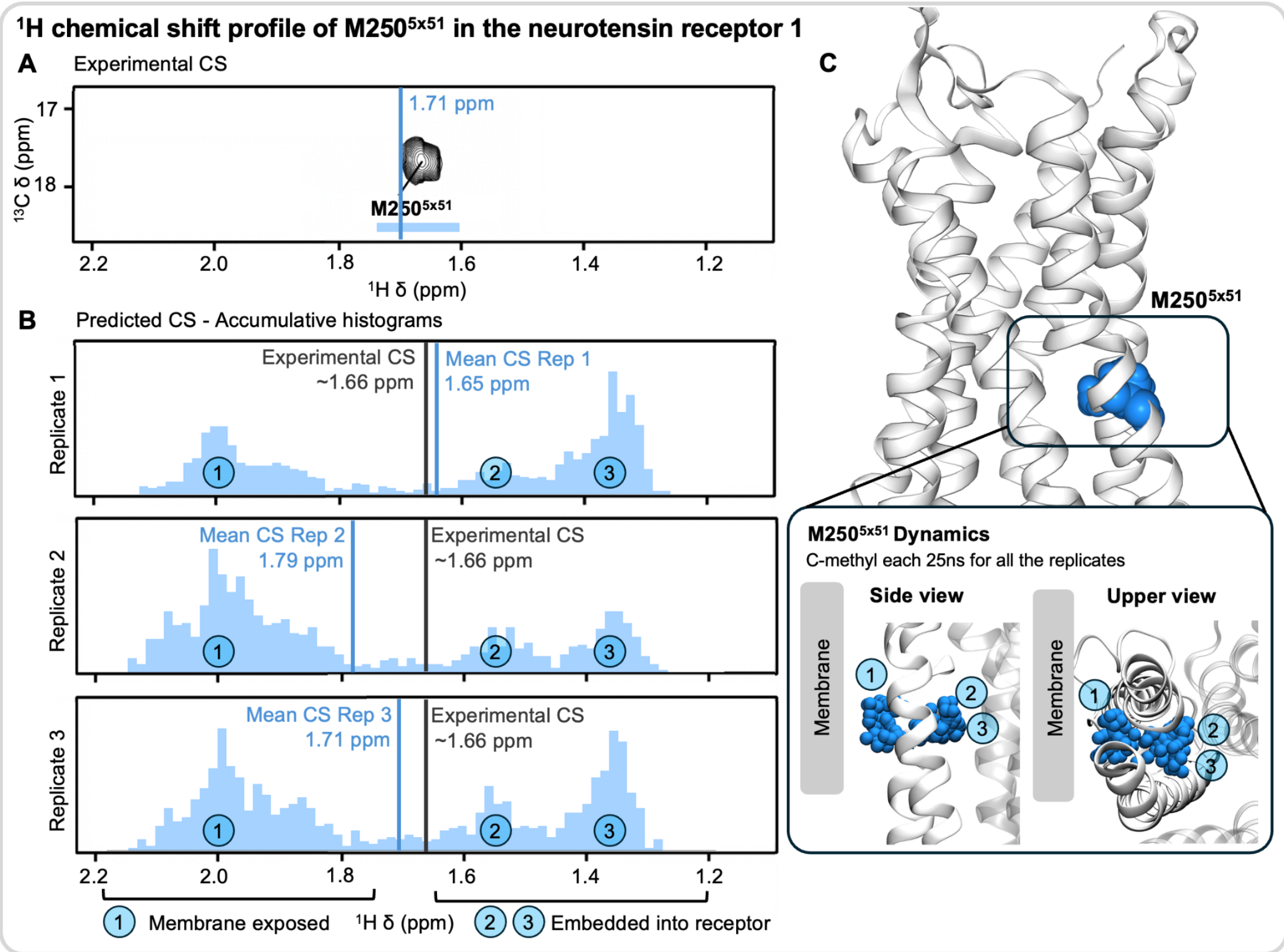

Figure 3. Linking conformational states and chemical shift variability of methionines in the NTR1 and GPCRmd-NMR potential applications (A) ^{1}H chemical shift profile of M250^{5x51} in the neurotensin receptor 1. Experimental CS data for M250^{5x51} extracted from Bumbak et al. [34], **Figure 2**). (**B**) Predicted CS data for M250^{5x51} over a distribution of different methyl conformations based on 3 x 1 μs simulations. The average predicted CS (1.71 ppm) matches the axis of the experimental CS (1.66 ppm). Such an averaging indicates the fast exchange of these sidechain conformations, in line with the MD findings. (**C**) Structural depiction of the neurotensin receptor 1 and the location of M250^{5x51} with different sidechain conformation.

Finally, this example highlights the unique value of our MD-based approach and its significant potential for method integration:

1. The MD approach reveals the explicit conformational space that generates the averaged spectroscopic reading. This allows for an explicit understanding of local structure plasticity and even microswitch states (see also **Figure 3**), bridging experimental biophysical data with receptor function and druggability.
2. This distribution is particularly relevant for low-temperature (LT) DNP solid-state NMR, which is a highly advanced emerging technique for mapping GPCR structures. In LT (low temperature, *ca.* 100 - 110 K) DNP solid-state NMR experiments, the protein samples are kept at a temperature similar to those used for cryo-EM. Therefore, the entire conformation distribution, rather than just the averaged value, can be observed (see **Figure 3B versus Figure 3A**).
3. Since this distribution is the "source" of the averaged CS value, our dataset provided new opportunities for integrating MD with an advanced NMR parameter sampling method developed in the NMR community (see also reply to Reviewer #2, point **R2.6.1**)

R2.4 In Fig 3C. Besides, the ‘membrane-exposed’ pose in MD versus ‘embedded-in-receptor’ pose in MD should be quantified (e.g. with distance/angles/exposed volume/exposed surface area) on top of the qualitative statement, if the bimodal behavior in predicted CS values were to be explained. Statistical tests e.g. Kolmogorov Smirnov test can be used to demonstrate that the two distributions from the binarized poses are distinct, if this is the claim that the author wants to express by right-shift due to poses. Further, it is unclear how Supplementary Fig S7C juxtaposes against experimental data as the plots shown are restricted to a specific MD trajectory, not an expectation from all available MD trajectories.

We thank the reviewer for suggesting this analysis. We performed conformational clustering over all MD frames and applied a Kolmogorov–Smirnov test, which yielded important new insights. We identified four statistically distinct conformational clusters of the M250 side chain (new Supplementary Figure 18) that do not directly correlate with the CS populations shown in Figure 3. For instance, we find one conformational cluster embedded in the receptor (cluster 3) but two CS populations at ~1.35 ppm and 1.55 ppm. This indicates that conformation alone does not always correspond to the observed chemical shift values.

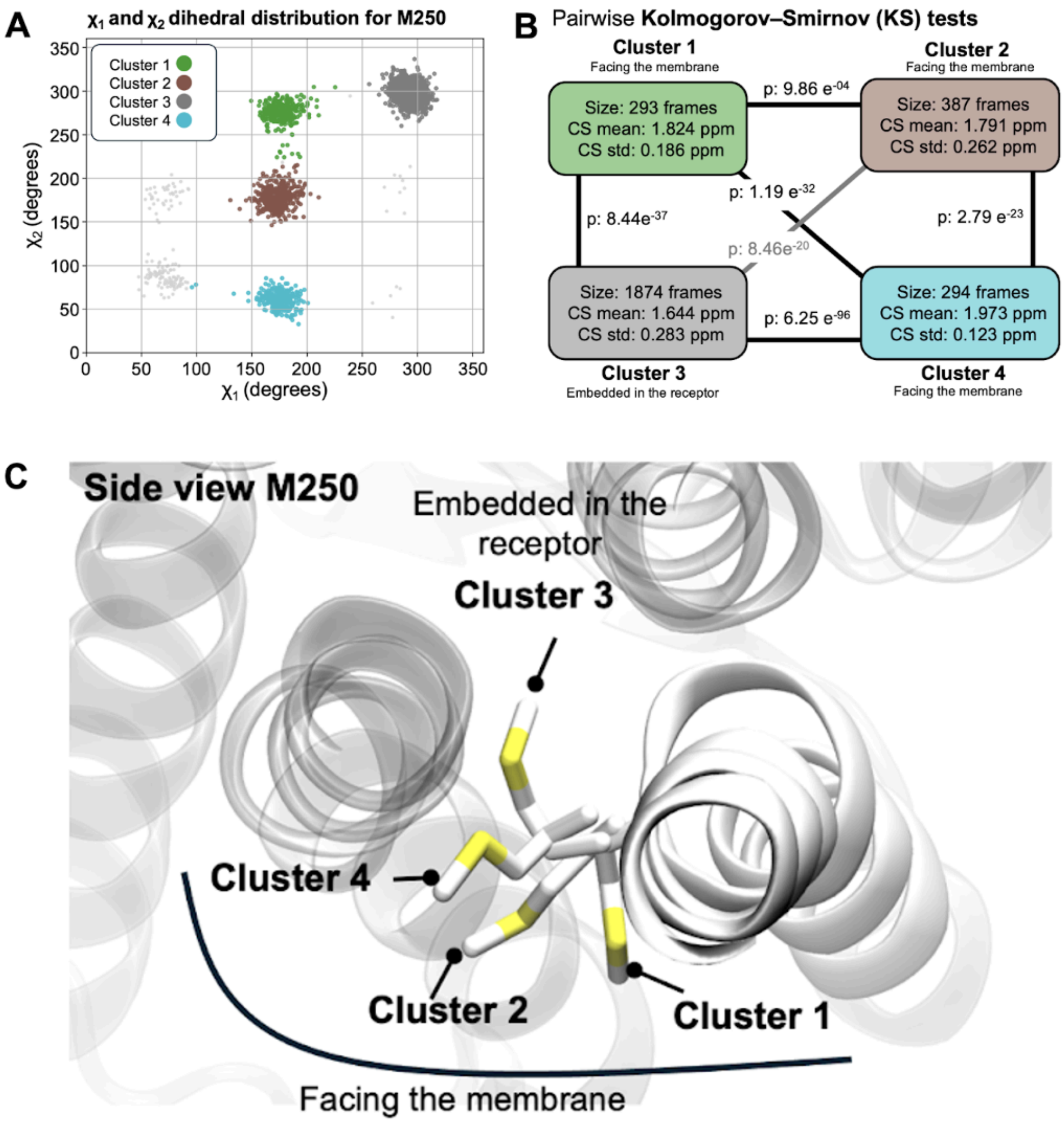

Supplementary Figure 18. Conformational landscape of M250 χ-dihedrals. (**A**) $\chi 1$–$\chi 2$ dihedral distribution for M250 across all three MD replicates. Each point represents one trajectory frame, colored by the four conformational clusters identified using an unsupervised

density-based algorithm (OPTICS): Cluster 1 (green), Cluster 2 (brown), Cluster 3 (grey) and Cluster 4 (cyan); unassigned points are shown in light grey. **(B)** Pairwise Kolmogorov–Smirnov tests comparing CS distributions between clusters. For each cluster, the number of frames, mean CS, and standard deviation are provided. Lines indicate p-values, demonstrating significant differences in CS between clusters. **(C)** Structural representation of M250 cluster centroids. Clusters 1, 2, and 4 point toward the membrane, while Cluster 3 is embedded in the receptor. Interestingly, the receptor-embedded M250 exhibits two distinct CS populations (see Main Text, Figure 3), yet only a single conformational cluster (cluster 3) is observed. This indicates that the CS signals are not determined solely by the M250 χ dihedral angles, but also reflect changes in the local environment.

We have now included this interesting finding into the main manuscript as follows:
"An interesting observation is that the observed CS populations arise from a heterogeneous set of M250$^{5\times51}$ sidechain conformations. For example, although the membrane-embedded M250$^{5\times51}$ displays two distinct CS populations (Figure 3), only a single conformational cluster (cluster 3; Supplementary Figure 18) is detected. This finding indicates that chemical shifts and conventional conformational descriptors do not exhibit a strict one-to-one correspondence, underscoring their complementary nature in capturing receptor microstate heterogeneity."

R2.5. I downloaded some of the PDB and XTC files, corresponding to several figures in this article, from GPCRmd ID: 85 for 5HT and GPCRmd ID: 872 for rhodopsin. E.g. https://gpcrmd.org/dynadb/files/Dynamics/17954_dyn_872.pdb To my surprise, in the model PDB files, the 7TM while is embedded in the lipid, there is a thick layer of "man-in-the-middle" water blocking the 7TM's access to interact with the lipid. It seems that the system assembly may have some glitches. While due to the large size of the XTC files, I can only check into one of the trajectories, the thick layer of "man-in-the-middle" water is not present, but it still makes me wonder if the glitch is cured in all other trajectories presented?

We appreciate the reviewer's careful examination of the PDB and XTC files. The observed water molecules are initially placed throughout the system during the system-building step (see the initial system snapshot below). Their positions are subsequently adjusted during our equilibration protocol within a few nanoseconds. As the reviewer can verify, no water molecules are present in the starting frame of the production simulations for any of the replicates. We have now added a brief note to the download section on the server to clarify this point.

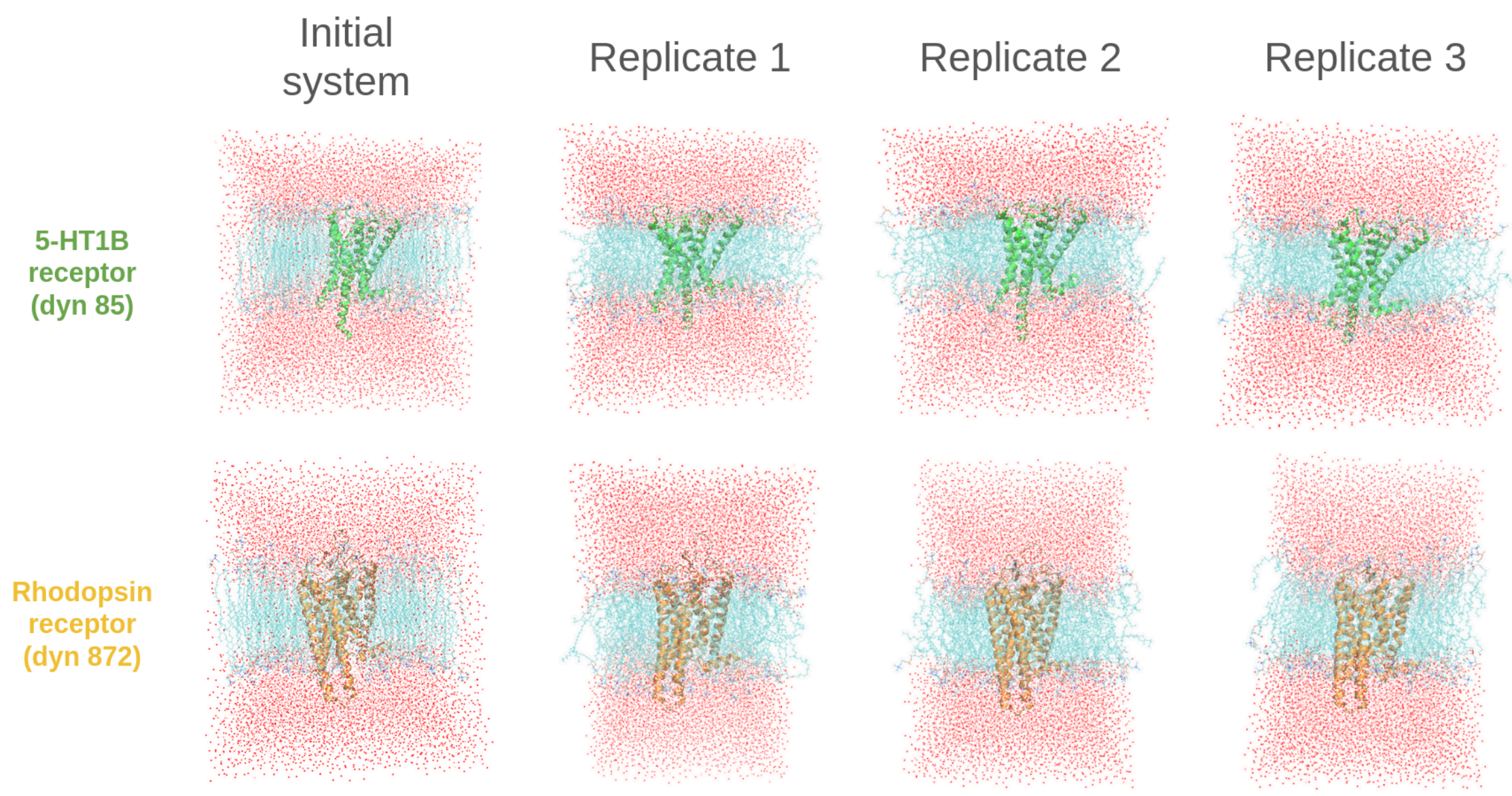

Figure for the reviewer. Removal of initially placed water molecules during equilibration. Snapshots of the initial system (left column) and three independent production replicates for the 5-HT1B receptor (GPCRmd ID: 85, top row) and rhodopsin (GPCRmd ID: 872, bottom row) embedded in a lipid bilayer.

R2.6 Indeed, it is questionable whether the types of mechanistic insights claimed e.g. microswitch transitions, hidden ligand entry pathways, or functionally relevant conformational states, can be robustly captured by standard MD simulations limited to a few microseconds per trajectory. Without the use of enhanced sampling techniques or statistical frameworks like Markov state models, the explored conformational space is likely insufficient to draw reliable mechanistic conclusions, particularly for complex systems like GPCRs known for their slow and rare transitions.

R2.6.1 Timescale issue

We fully agree with the reviewer that this issue was not sufficiently addressed in our previous manuscript. We have now added a new section to the main manuscript, along with a corresponding figure, specifically dedicated to addressing this challenge.

"Bridging the MD-NMR gap at distinct time scales

It is well known that the time scale of conformation interconversion of proteins determines the NMR observable: when exchange occurs at the slow time scale, NMR spectra present individual signals for each state, whereas fast exchange results in a single population-weighted averaged signal …"

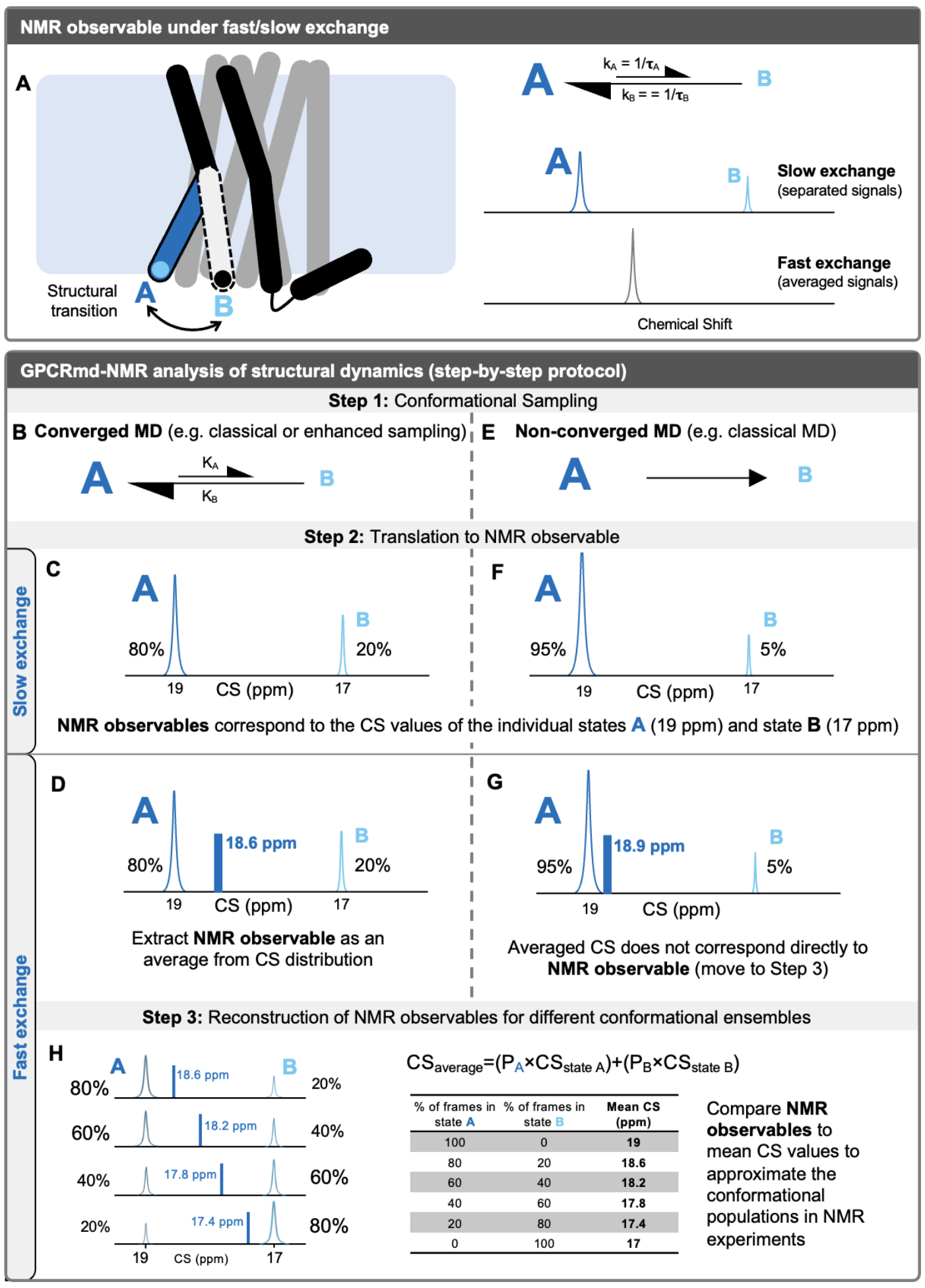

% of frames in state A	% of frames in state B	Mean CS (ppm)
100	0	19
80	20	18.6
60	40	18.2
40	60	17.8
20	80	17.4
0	100	17

Figure 6. Bridging MD simulations and NMR observables across different timescales. (**A**) The NMR signal depends on the rate of conformational exchange: slow exchange produces separate peaks for each state, whereas fast exchange yields a single

population-weighted averaged signal. (**B**) Converged MD achieves equilibrium sampling between conformational states A and B. (**C**) NMR detects separate peaks corresponding to state-specific chemical shifts. (**D**) NMR observable approximated by averaging CS values across the equilibrated ensemble. (**E**) Non-converged MD between states A and B. (**F**) Distinct NMR peaks still reflect individual state CS values. (**G**): Averaged CS does not match the experimental NMR observable. (**H**): Population-weighted reconstruction of NMR observables using state-specific CS values and estimated conformational populations.

R2.6.2 While the future is wide-open, it remains unclear how the current GPCRmd-NMR dataset has meaningfully addressed these limitations or enables deeper mechanistic understanding.

We thank the reviewer for prompting us to clarify and better highlight the value of the GPCRmd-NMR dataset in this respect. In the current work, we address the current limitations at two complementary levels: (**1**) through a general integrative framework that links MD simulations and NMR observables at distinct timescales, and (**2**) through a curated, large-scale dataset that enables systematic comparative analyses across GPCRs.

1. An integrative approach to provide mechanistic insights at distinct timescales. GPCRmd-NMR is intended as an integrative link between experimentally measured NMR observables and explicit, MD-derived structural insights at distinct timescales. By associating predicted CS patterns with specific conformational microstates and transitions, the framework supports the interpretation of NMR signals in terms of underlying conformational heterogeneity and local conformational exchange processes. Importantly, these insights have demonstrated significant potential to advance our mechanistic understanding of receptor function, including: **(A)** detection of entrance gateways, **(B)** characterization of binding site plasticity, **(C)** study of overall receptor activation, (**D**) involvement of specific microswitches, and **(E)** the role of intrinsically disordered C-tail regulation.

A. **Visual rhodopsin: lateral gateway dynamics.** Lipid tail insertion between TM5/TM6 correlates with a strong shift in a label-friendly **V204 ^{1}Hγ** signal as the cavity opens. *Mechanistically, this establishes a direct spectroscopic readout of transient membrane-coupled gateways that regulate ligand and lipid access to the receptor core.*
B. ***5-HT2B: orthosteric pocket plasticity.*** *We uncover multi-state dynamics of the conserved pocket residue* ***F6.52****, where rapid rotameric switching dynamically reshapes the orthosteric cavity. In conventional NMR, such fast exchange would likely appear as a single averaged signal, obscuring the underlying microstates. Mechanistically, this structural plasticity provides a basis for ligand recognition and signaling efficacy.*
C. **μOR: activation reporters from state-resolved ensembles.** Separation of active-like and inactive-like ensembles reveals strong backbone ^{15}N CS changes in canonical microswitch regions (TM6, ICL3, NPxxY) and previously underappreciated interior sites (e.g., Y326^{7x42}). *Mechanistically, these residues define a distributed activation network that can serve as spectroscopic reporters to dissect how different ligand classes fine-tune receptor conformational equilibria.*
D. ***NTR1: fast side-chain exchange near the TM5 activation hinge.*** *The methyl dynamics of* ***M250***^{5x51}*, adjacent to the conserved TM5 proline kink, reveal rapid conformational exchange. While the experimental NMR spectrum reports a*

single averaged peak (Bumbak et al), GPCRmd-NMR resolves the underlying ensemble of interconverting states. Mechanistically, this residue acts as a local reporter of the TM5 hinge that propagates ligand-induced motions toward the intracellular activation machinery.

E. **β2AR: intrinsically disordered C-tail regulation.** We have now also extended GPCRmd-NMR to probe **IDR dynamics** in the receptor C-terminus, resolving disorder-to-order transitions and transient contacts with the receptor core. *Mechanistically, these dynamic interactions suggest a regulatory role for the C-tail in modulating G protein coupling and intracellular signaling interfaces.*

2. Potential of the current GPCRmd-NMR dataset to support studies of GPCR functional mechanisms

Mechanistic studies across the GPCRome. The examples discussed above illustrate the potential of the GPCRmd-NMR framework to reveal mechanistic insights using only a small fraction of the current database. A key strength of the existing GPCRmd-NMR dataset is its consistently curated structural and dynamical information, which allows researchers to examine functionally relevant features within a unified and highly accessible framework across the GPCRome (see entire dataset in Figure 1 main text). Therefore, the GPCRmd-NMR dataset in combination with targeted NMR validation experiments supports systematic investigation of, for instance, ligand gateways characterization of binding sites plasticity with a strong implication for ligand recognition, receptor activation, and the involvement of specific microswitches or even the involvement of intrinsically disordered C-tail regulation across diverse GPCR types. This comparative framework facilitates hypothesis generation on how evolutionary sequence variations correlate with differences in dynamic behavior and functional properties.

Study of frequent and rare conformational transition events (fast and slow conformational exchange) implicated in GPCR function. Importantly, the dynamic coverage of the GPCRome in the current GPCRmd-NMR dataset provides a basis for relating NMR observables to heterogeneous conformational populations, thereby contributing to a better mechanistic understanding. This approach is directly applicable to fast conformational exchange processes, such as side-chain rotamer switching across GPCRs in the dataset, but also to slower transitions, including receptor activation and inactivation events. This is possible, as many GPCR systems in the current dataset sample diverse conformations along activation pathways (see dataset description in Aranda et al., *Nat. Commun.*, 2025). Leveraging these structural insights together with NMR-guided reconstruction enables us to approximate conformational heterogeneity across different scenarios (see new section “Bridging the MD–NMR gap at distinct time scales”). This represents a major strength of the GPCRmd–NMR dataset for addressing mechanistic questions of GPCR function.

Ultimately, considering the constant growth of the GPCRmd database (see figure below), our GPCRMD-NMR will keep expanding the open NMR/data access to these functionally relevant aspects.

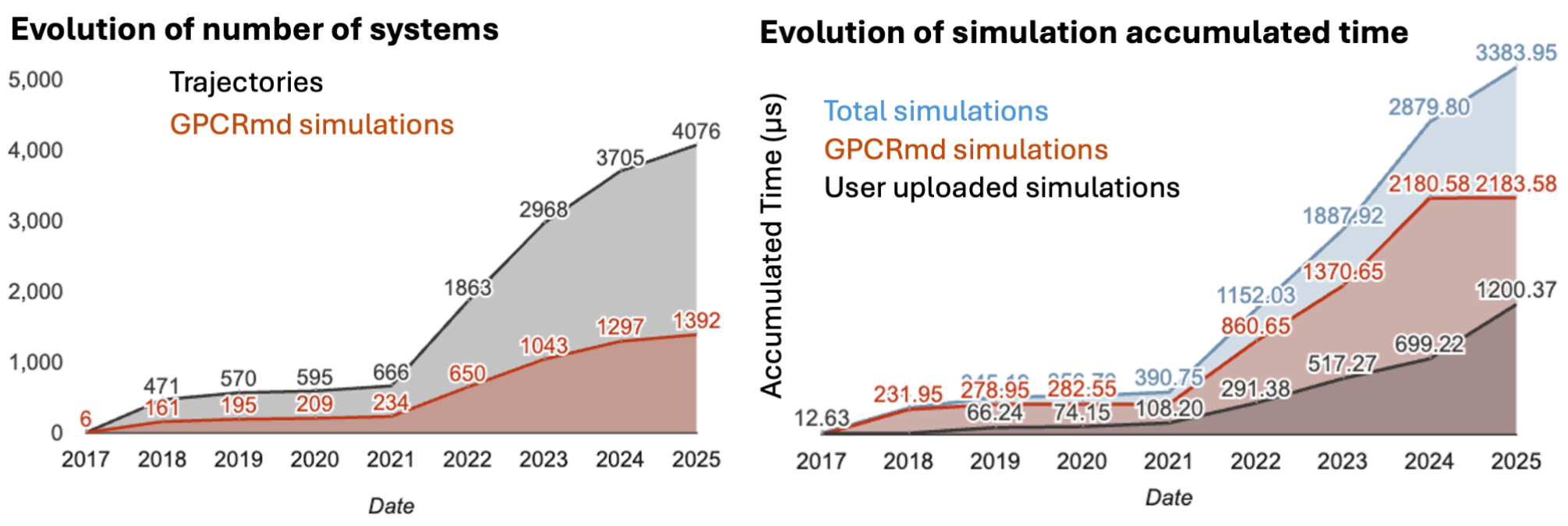

Evolution of GPCRmd data. Left: Evolution of the number of systems available in GPCRmd up to 2025. Grey indicates the total number of trajectory files available up to 2025, while orange represents the total number of independent GPCR systems. Right: Evolution of simulation accumulated time up to 2025 (in microseconds). Blue shows simulation time from user-submitted MD trajectories, orange corresponds to GPCRmd community simulations, and black represents the combined total.

We have now clarified this better in the discussion section.

*"Beyond establishing an overall MD–NMR integrative framework, the generated GPCRmd–NMR dataset and its broad coverage of different GPCR types (**Figure 1B**) provides researchers with a unique opportunity to interrogate specific mechanistic aspects of their GPCR of interest. As shown in this study, this includes the identification of transient ligand and lipid entry gateways and their coupling to spectroscopic reporters, quantification of orthosteric pocket plasticity and its impact on ligand recognition. The framework further enables characterization of fast sidechain exchange processes at key activation hinges, dissection of microswitch coupling during receptor (in)activation, and analysis of disorder-to-order transitions in intrinsically disordered regions, such as C-terminal tails that regulate signaling interfaces. The exploration of conformational events linked to other functional aspects can be facilitated by our open-accessible protocol templates provided at GitHub (https://github.com/GPCRmd/GPCR_Chemical_Shift). Importantly, the broad coverage of the GPCRome in the current dataset also enables systematic investigation of how sequence variation shapes receptor dynamics and signaling mechanisms, providing comparative insights into the principles governing GPCR function. Beyond the current dataset, GPCRmd–NMR dataset is constantly growing and will rapidly expand the open and transparent NMR/data access to multiple functionally relevant aspects, encouraging novel integrative biophysical studies of these important receptors."*

Minor Comments

R2.7. Fig 2A. The left-hand-side color code does not fully correspond with the right-hand-side figure. For example, what are cyan and purple? Similarly in the SI figures.

We have updated **Figure 2A** and the corresponding **SI figures** so that only the signals associated with the highlighted atoms are colored, while all other signals are shown in light grey.

R2.8. Fig 2D, Tables should be taken out of the figure as a separate table. It is unclear visually what the numbers are.

We have removed the table from **Figure 2D** and created a comprehensive table for each replicate in **Supplementary Figures 2-16**, as well as in **Supplementary Table 1**, which

presents all information across replicates and predictors. Additionally, each graph now includes the correlation coefficient (r) and RMSE, and the plots have been redesigned.

R2.9. On page 11, NTS1 and NST1 were used interchangeably for the neurotensin receptor. The acronym is complicated by additional use of NTSR1 in main text. So, which acronym is chosen?

We have standardized the terminology throughout the manuscript; the acronym NTR1 is now used consistently to refer to the neurotensin receptor.

R2.10. On page 11, Citation for Prime Maestro should be provided.

We have included the citation for Prime Maestro.

R2.11. On page 12, 4 femtoseconds intervals were used in the MD simulation but the author did not justify the use of this larger timestep than the commonly used 2 femtoseconds interval. Were techniques in accelerated MD were actually used? Please state in text. Besides, it is not clear what barostat/thermostat were used in the NPT and NVT simulations.

Thank you for highlighting this point. We have now added a justification in the main manuscript for the use of a 4 fs timestep. For the larger timesteps, we employed the hydrogen mass repartitioning (HMR) technique, which allows for larger timesteps while maintaining system stability. We have also provided specific details regarding the thermostat and barostat used for the NVT and NPT ensembles in the Methods section.

Reviewer #2 (Remarks on code availability):

R2.12 It seems that the authors only provided codes to plot graphs from csv data, but not the source code where MD trajectories are analyzed and are used to compute the CS values per conformation.

Thank you for this comment. We have incorporated into the GitHub repository (https://github.com/GPCRmd/GPCR_Chemical_Shift/tree/main) the full collection of scripts used to compute chemical shifts from MD trajectories and integrate them into GPCRmd-NMR. The repository also includes links and instructions to reproduce all the case studies presented in this publication.

Reviewer #3 (Remarks to the Author):

This manuscript introduces GPCRmd-NMR, a platform integrating molecular dynamics (MD) simulations with nuclear magnetic resonance (NMR) spectroscopy data for G protein-coupled receptors (GPCRs). The work addresses the challenge of obtaining high-resolution information on protein dynamics.

The platform contains a comprehensive dataset of predicted NMR signals mapped to GPCR conformational snapshots, enabling structure-resolved analysis at scale. The authors provide analysis tools that help interpret protein dynamics that are difficult to capture through either experimental or computational methods alone. Several case studies demonstrate the platform's utility, and the resource appears valuable for data mining and hypothesis generation.

The integration of protein dynamics and data science is timely and very relevant to current structural biology research. By providing open access to biophysical data through a web-based framework, the authors create a resource potentially useful to researchers across disciplines.

The manuscript is clearly written, methodologically sound, and contains informative and well-designed figures. The approach can extend to other protein families and biophysical data types, suggesting broader relevance beyond GPCRs.

Still, there is one point that I think needs further clarification.

Major comments

R3.1. The authors note in the text and Figure 2C/2D that predictions for HN atoms (amide protons) show relatively low correlations with experimental values (R^2 = 0.42 to 0.46),and explain that "1H chemical shifts are more sensitive to subtle inaccuracies in structural or electronic modeling." However, in subsequent analyses, the authors rely on different types of hydrogen chemical shifts - specifically methyl hydrogen shifts (HE) in Figure 3A/B for methionine side chains and valine methyl hydrogen shifts (Hγ) in Figure 4I.

We thank the reviewer for raising this point. We agree that we did not comment clearly on this issue and why we used the methyl proton.

Polar protons (e.g., ^{1}HN) are typically associated with higher errors compared to non-polar protons (e.g., ^{1}HC) (PMID: 26583557 39531038). This is because amide proton shifts are highly sensitive to hydrogen bonding geometry, solvent exposure, and electrostatics, making them challenging to predict accurately with empirical or ML-based predictors. In contrast, methyl protons are not directly involved in hydrogen bonding and undergo rapid rotational averaging, which reduces sensitivity to local structural noise. Our case study serves as a clear proof of concept that methyl protons (^{1}HC) can effectively describe fast conformational transitions, as demonstrated in **Figure 3**. The computed average CS aligns well with the experimental NMR observables.

We have now improved the text and the justification as follows:
"*In addition, this case highlights that methyl protons can serve as reliable predictors of fast sidechain exchange. This observation is consistent with previous reports showing that protons not influenced by electronic shielding carry less predictive errors in contrast to donor protons (e.g., NH) Zhu et al. 2013; Ptaszek et al. 2024*."

R3.2. The authors do not seem to clarify whether the reliability limitations observed for amide protons also apply to these other types of hydrogen atoms. The authors could address this point by either providing correlation statistics specifically for methyl hydrogen predictions if available, by explaining why methyl hydrogen predictions might be more reliable than amide hydrogen predictions, or by acknowledging any potential limitations when drawing conclusions from these hydrogen shift analyses.

We thank the reviewer for pointing out this very intriguing observation. We have compiled a large list of 13C chemical shift benchmarks for GPCRs in this revision (see also **R2.1.2**). Regarding the methyl protons, there are only very few records in the literature/BMRB database (BMRB: 27292, 27261, totalling 10 datapoints included in **Supplementary Figure 18**). Therefore, a statistically meaningful correlation could not be provided. Following the reviewer's further suggestions, we have then focused on examining the reliability of methyl vs. amide ^{1}H chemical shift prediction. The clear difference in their performance has already been addressed well in the literature (both QM calculations and empirical benchmarking). The details can be found in **R3.1** and the revised main text.

Minor comments

R3.4. In "Supplementary Note 1," the text states: "Figure 3C illustrates the 'Two-Dimensional Chemical Shift' mode, where D79(2.50) has slightly greater fluctuation in CS compared to D192(45x51) for the Cα and Nitrogen atoms." This appears to be an error and should reference Figure 4C instead.

Thanks for pointing this out. We have corrected it.

R3.5. The supplementary material also contains citation inconsistencies. For example, in "Supplementary Note 1," the text mentions: "A snapshot from GPCRmd highlighting the main features of both modes is presented in Supplementary Figures 4-5." The authors should verify all supplementary figure citations for accuracy.

Thanks for this comment. We have revised the labelling for each figure.

Reviewer #4 (Remarks to the Author):

The authors used chemical shift prediction software to generate tremendous amount of computed NMR chemical shift signals for frames in GPCR trajectories in GPCRmd, and try to align these computed chemical shifts with experimental data, and eventually use these computed chemical shifts to generate hypotheses and guide experimental designs. I have big concerns on both the methods and results of this manuscript.

R4.1 SHIFTX2 and SPARTA+ are methods developed around 15 years ago. The training data mainly covers small soluble proteins. By then, NMR data for large membrane proteins such as GPCRs were extremely limited, so this kind of data should be scarce in the training data of the software. I assume the performance of SHIFTX2 and SPARTA+ on GPCRs may be not that reliable due to this reason.

We thank the reviewer for raising this important point. We agree that SHIFTX2 and SPARTA+ were primarily trained on small soluble proteins, and experimental NMR data for large membrane proteins, such as GPCRs, were limited at that time.

In this respect, our study serves as a great benchmark study showing that overall residues are well predicted by SHIFTX2 and SPARTA+ when compared to experimental NMR data (**Supplementary Figure 2 to 11).**

For membrane-facing residues, we aimed to validate the computed CS values against experimental measurements derived from BMRB. However, the lack of high-quality experimental CS data for membrane-facing residues in GPCRs makes this validation currently unfeasible for us. We agree that the user should be aware of this limitation. To address this, we are now including a note in the GPCRmd-NMR interface that flags queried residues as membrane-facing, advising users to interpret these predictions with caution.

Implemented note on the GPCRmd-NMR interface:
"*CS predictors may be less accurate for membrane-facing residues. Please interpret such CS values with caution.*"

Additionally, we have integrated UCBShift 2.0 (PMID: 39531038), one of the most recent chemical shift predictors from 2024, into the GPCRmd-NMR framework (Correlation scatter plots in **Supplementary Figures 12-16**). Our benchmarking, summarized in **Supplementary**

Table 1, shows comparable correlation coefficients across all CS predictors for heavy atoms. As a final note, the GPCRmd-NMR framework is designed to easily implement any future state-of-the-art CS predictors as they become available.

R4.2 The authors used a few GPCR cases (a few residues in B1R and NTSR1) to "benchmark" the accuracy of predicted chemical shifts. I think this is not enough. There are accumulated GPCR NMR data in recent years, thanks to the efforts of several research groups, such as Ichio Shimada, Stephan Grzesiek, R. Scott Prosser, and Kurt Wuthrich. I suggest the authors compare their computed chemical shifts with reports/results from these labs' NMR research on various GPCRs. Besides, the authors did not carefully analyze why HN chemical shifts cannot be accurately predicted.

We thank the reviewer for this important suggestion. We fully agree that benchmarking against a broader set of GPCR NMR datasets is essential. In response, we compiled all accessible GPCR NMR datasets available in the BMRB database, thereby substantially expanding our benchmarking analysis. This expanded dataset includes five additional GPCR NMR studies (BMRB: 27292, 52783, 27261, 51648, 51907), summarized in **Table 1** (Experiments 8–12), comprising a total of 669 new experimental CS values used for validation. The aggregated benchmarking results are shown in **Supplementary Figure S17**. Overall, these expanded benchmarks confirm the trends observed in our original analysis, including strong performance for heavy atom chemical shifts and lower performance for proton chemical shifts.

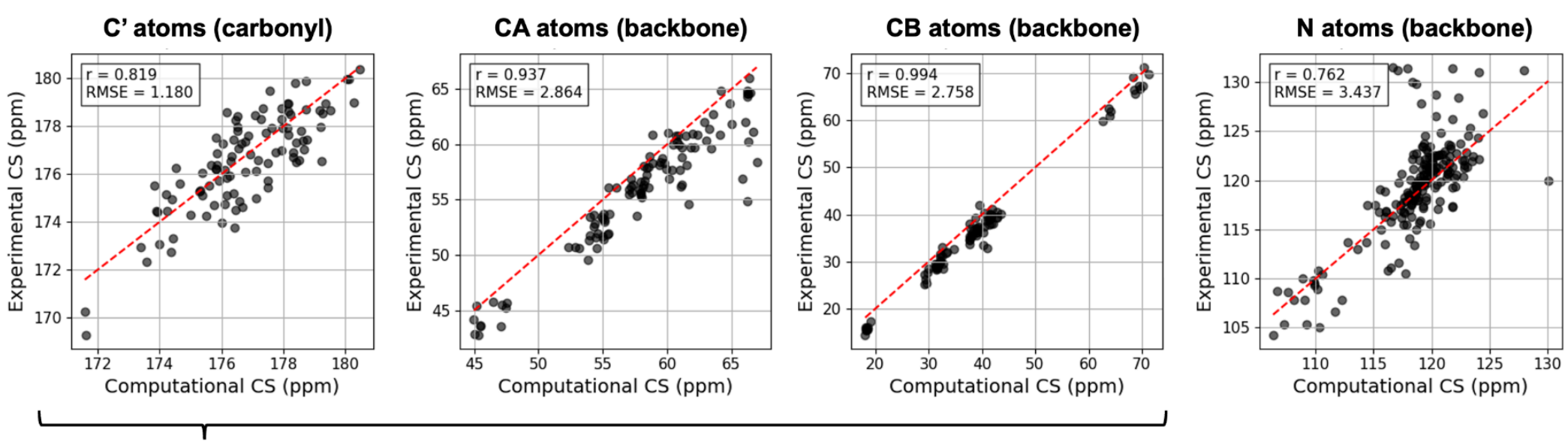

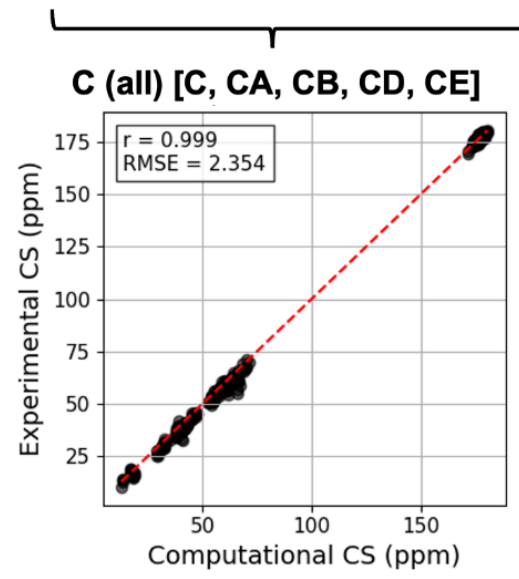

Atom type	Pearson r	RMSE	Exp. data points	Experiment IDs
C' (carbonyl)	0.819	1.180	101	12
CA	0.937	2.864	106	12
CB	0.994	2.758	86	12
N	0.762	3.437	188	9, 11, 12
H	0.321	0.780	188	9, 11, 12
C (all)	0.999	2.354	303	8, 9, 10, 11, 12

Supplementary Figure 17. *Benchmarking of GPCRmd-NMR CS prediction against NMR experimental datasets. The table presents the benchmarking results of GPCRmd-NMR CS predictions against experimental data from diverse studies (IDs 8–12). All experimental datasets were combined into a single correlation matrix, and the performance of the CS predictor ShiftX2 was evaluated across different atom types. The reported number of experimental data points corresponds to the total CS signals obtained from studies with IDs 8–12. Detailed information about each receptor system included in these experiments (Experiment ID) is provided in Table 1.*

R4.3 The timescale of MD simulation and NMR chemical shifts are so different that they barely overlap with each other. The timescale of MD simulation is usually at nanoseconds to microseconds, while the timescale of chemical shifts in NMR is at microseconds to milliseconds. Even though the authors calculated huge number of chemical shifts, in experiments, it's very probably only averages of these chemical shifts can be observed. Thus, it's very difficult to align

chemical shifts from the two methods, people must be very careful to not make mistakes in hypothesis generation based on computed chemical shifts.

We thank the reviewer for highlighting this important aspect, which was also noted by the other reviewers. We have now comprehensively addressed this issue in a new section entitled “Bridging the MD–NMR gap at distinct time scales” in the main text. Please also refer to our response to Reviewer 2, point **R2.6.1.**

R2.6.1 Timescale issue

We fully agree with the reviewer that this issue was not sufficiently addressed in our previous manuscript. We have now added a new section to the main manuscript, along with a corresponding figure, specifically dedicated to addressing this challenge.

“Bridging the MD-NMR gap at distinct time scales

It is well known that the time scale of conformation interconversion of proteins determines the NMR observable: when exchange occurs at the slow time scale, NMR spectra present individual signals for each state, whereas fast exchange results in a single population-weighted averaged signal …”

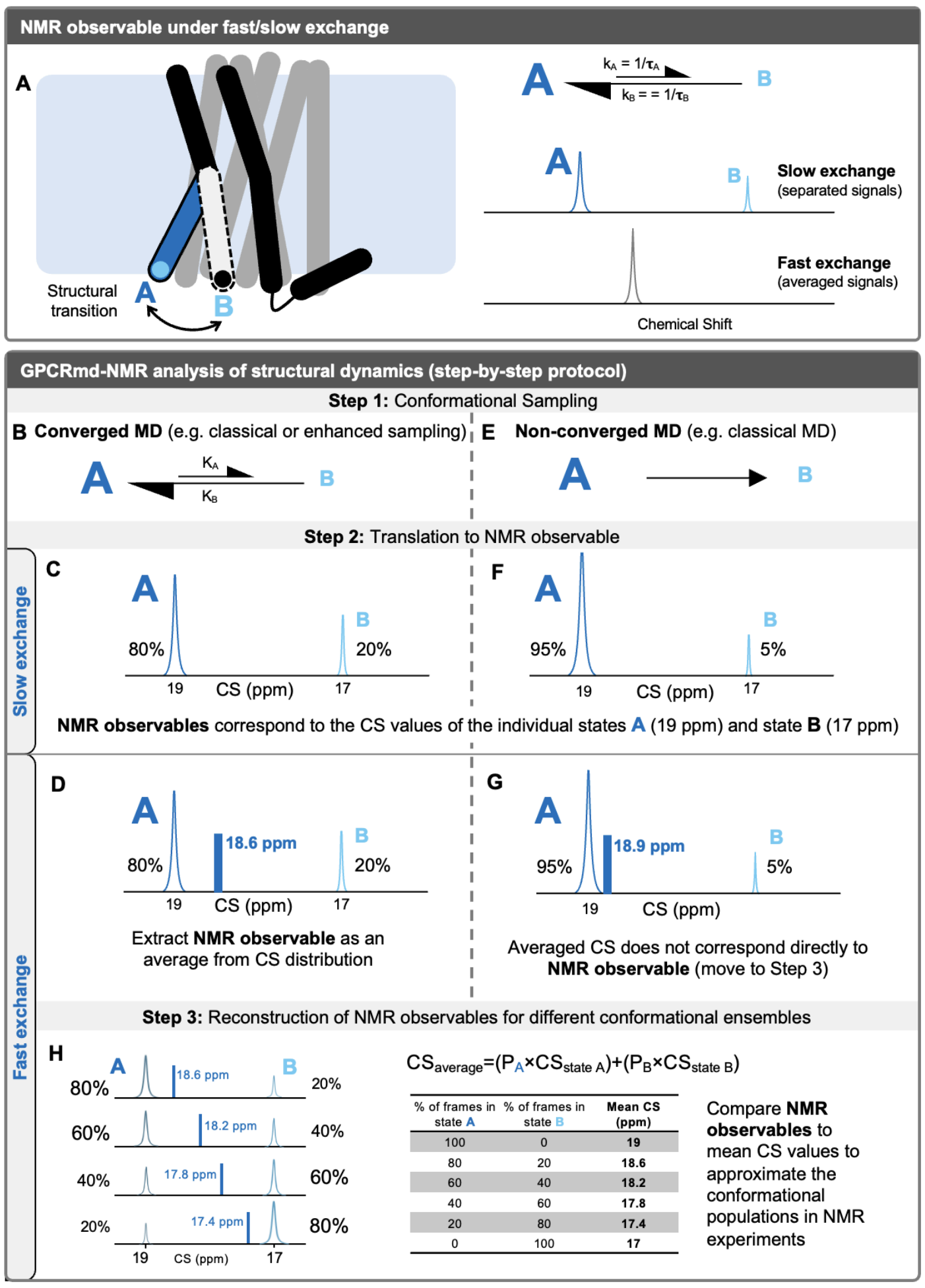

% of frames in state A	% of frames in state B	Mean CS (ppm)
100	0	19
80	20	18.6
60	40	18.2
40	60	17.8
20	80	17.4
0	100	17

Figure 6. Bridging MD simulations and NMR observables across different timescales. (**A**) The NMR signal depends on the rate of conformational exchange: slow exchange produces separate peaks for each state, whereas fast exchange yields a single population-weighted averaged signal. (**B**) Converged MD achieves equilibrium sampling between conformational states A and B. (**C**) NMR detects separate peaks corresponding to state-specific chemical shifts. (**D**) NMR observable approximated by averaging CS values across the equilibrated ensemble. (**E**) Non-converged MD

between states A and B. (**F**) Distinct NMR peaks still reflect individual state CS values. (**G**): Averaged CS does not match the experimental NMR observable. (**H**): Population-weighted reconstruction of NMR observables using state-specific CS values and estimated conformational populations.

R4.4 Many MD trajectories contain "broken" conformation due to the imperfectness of this method, using multiple repeats is one solution but it cannot rule out such scenarios. Given the authors do not filter these conformations out before calculating chemical shifts, wrong dynamics will be used to generate hypothesis.

We thank the reviewer for raising this important concern. We are fully aware of these potential issues and have implemented a rigorous protocol to minimize such artifacts. All MD simulations in our dataset undergo systematic curation and quality control. This includes verifying thermodynamic stability (pressure and volume), proper protein geometry enforced by the force field (bond lengths, dihedral angles/torsions, van der Waals and electrostatic interactions), absence of structural clashes or ring penetrations, accurate ligand parameterization, correct protein–membrane alignment, appropriate amino acid protonation states, and terminal capping. Any system that fails to meet these criteria or exhibits instability or structural artifacts is excluded from the dataset during the curation process.
We are confident that these measures provide a well-curated dataset. Moreover, our benchmarking analyses show strong correlations with the experimental data, indicating that potential simulation artifacts do not represent a significant concern.

R4.5 GPCR functional conformational change (such as activation) usually happens at microseconds to milliseconds timescale. MD simulations at microsecond-timescale cannot catch such meaning conformational changes, thus chemical shifts calculated from MD simulations will lean to missing important conformational change, thus they cannot cover full energy landscape.

We have now comprehensively addressed this issue in a new section entitled "Bridging the MD–NMR gap at distinct time scales" and a corresponding Figure 6 in the main text. In this new section, we highlight how GPCRmd-NMR can capture and present such rare but fast conformational events. We have also provided a step-by-step protocol for implementing diverse approaches (e.g., converged vs. non-converged MD, advanced sampling, NMR data-guided conformational space reconstruction) for resolving this important aspect. For additional details, please also refer to our response to Reviewer 2, point **R2.6.1.**

Dear Editor,

We thank all the reviewers for their positive feedback. In the revised manuscript, we have addressed all remaining minor comments and suggestions below.

We hope that the manuscript is now suitable for acceptance.

Best regards,
The Authors

Reviewer #1

R1.1 I found the additional benchmarks quite useful. The new paper reads well and my questions were answered. It's strength lies in the open source platform for others to use and contribute to.

We thank the reviewer for the positive assessment of our work and for recognizing the value of the additional benchmarking analyses and the open-source nature of the platform.

Reviewer #2

I thank the authors for their detailed rebuttal and for the substantial revisions made to the manuscript. The additional benchmark against experimental BMRB datasets, the removal of the circular-validation claim, the new quantitative analyses, and the expanded discussion have addressed the major concerns raised in my original review. In my opinion, the manuscript has improved significantly and is now suitable for publication after minor revision. I have only two remaining points that should be addressed before acceptance:

R2.1. Avoid claims of MD convergence based solely on agreement with experimental chemical shifts. The revised manuscript appears to imply that agreement between predicted and experimental chemical shifts demonstrates that the underlying MD ensemble is well converged. I do not believe such a conclusion is warranted. Agreement with one or a limited set of observables can indicate consistency with experiment, but it does not by itself establish convergence of the ensemble. I recommend removing or softening any statements that suggest experimental agreement proves MD convergence, and instead describing the agreement as supportive evidence for the relevance of the sampled conformations.

We thank the reviewer for this comment and agree that agreement between predicted and experimental chemical shifts alone does not demonstrate convergence of the underlying MD ensemble. Accordingly, we have removed the statements that linked agreement with experimental data to MD convergence.

"Importantly, the close agreement of the average CS across the different CS populations with the experimental NMR signal supports the relevance of the sampled conformational space."

R2.2 Explicitly state the limited availability of experimental GPCR NMR datasets. The authors have now performed a valuable exercise against all GPCR datasets currently available in the BMRB and report that only five GPCR cases were identified, yielding 679 chemical-shift measurements. This is an important contextual point that helps readers understand the limitations of experimental validation in this field. I recommend that the manuscript explicitly state, in the main text and/or discussion, that the benchmark was performed against all currently available GPCR entries in the BMRB (five cases, 679 chemical-shift values), rather than leaving this information only in the response to reviewers or supplementary material.

We thank the reviewer for this suggestion. We agree that this information provides important context for interpreting the scope of the validation. Accordingly, we have revised the manuscript to explicitly state in both the Results and Discussion sections that our benchmark includes all currently available GPCR entries in the BMRB.

In the Result Section:
"*These five datasets represent all currently available GPCR CS entries deposited in the BMRB, comprising a total of 679 experimental chemical-shift measurements.*"

In Discussion Section:
"*This approach was benchmarked against all available experimental NMR datasets from the BMRB, including chemical-shift datasets comprising 679 measurements.*"

"*A current limitation of experimental validation is the limited availability of GPCR NMR datasets. At present, five GPCR backbone chemical-shift datasets (679 measurements) are publicly available in the BMRB, all of which are included in our validation study.*"

Subject to these minor revisions, I support acceptance of the manuscript.

Reviewer #3

I have read the revised manuscript and the authors' responses to my previous comments.
My main concern in the first round was the reliability of the 1H chemical shift predictions. The authors have addressed this point satisfactorily. I appreciate that the authors also examined the available BMRB records for methyl protons and acknowledged transparently that the limited number of datapoints precludes a statistically meaningful correlation. I am satisfied that my comments have been resolved, and I have no further substantive concerns.

We sincerely thank the reviewer for their careful evaluation of the revised manuscript and for the positive assessment. We appreciate their recognition that our revisions have satisfactorily addressed their concerns.

Reviewer #4

This manuscript presents a comprehensive study bridging MD simulations and NMR analyses of GPCRs. The concerns raised in the previous round of review have been satisfactorily addressed, and the revised manuscript is now well developed.

We thank the reviewer for the constructive and positive feedback.

I have only a few minor comments:

R4.1 The first letter of the Latin name for rat should be capitalized.
Page 4; or Line 137, Page 35 in the original version.

The Latin name has been corrected throughout the manuscript.

R4.2 PDB 4IAQ is listed as a structure of 5-HT2BR on Pages 5 and 24; however, it corresponds to 5-HT1BR. Please correct this assignment.

The receptor assignment has been corrected throughout the manuscript.

R4.3 For 5-HT2B, the key residue is referred to as F311^6x52 in the main text on Page 5, but as F331^6.52 in Figure 2D on Page 23 and on Page 24. Please verify the residue number and correct the Ballesteros–Weinstein numbering label accordingly.

The residue has been corrected to F331^6×52 throughout the manuscript.

R4.4 Terminology should be made consistent throughout the manuscript.
For adrenergic receptors, different forms are used, such as “β_2 adrenergic receptor,” “β2 adrenergic receptor,” and “β2-adrenergic receptor.” Please use one consistent format.
For neurotensin receptor 1, both “NTSR1” and “NTR1” are used as abbreviations. Please standardize the abbreviation.

We have standardized the terminology throughout the manuscript. Adrenergic receptor nomenclature has been made consistent: β_2-adrenergic receptor (β_2AR), and for neurotensin receptor 1 we have adopted **NTS_1** receptor (**NTS_1R)** according to the IUPHAR nomenclature.

R4.5 Please correct the use and formatting of Greek letters.
The notation for side-chain dihedral angles should use the lowercase Greek letter χ, rather than the Latin letter X, in Figure 2D.
The fonts of α, β, and γ in the right panel of Figure 2A, and α in Figure 2C, appear inconsistent with those used elsewhere in the manuscript. Please standardize the formatting.

We have corrected the font for the side-chain dihedral angles in Figure 2C.
